# *DeePhys:* A machine learning–assisted platform for electrophysiological phenotyping of human neuronal networks

**DOI:** 10.1016/j.stemcr.2023.12.008

**Published:** 2024-01-25

**Authors:** Philipp Hornauer, Gustavo Prack, Nadia Anastasi, Silvia Ronchi, Taehoon Kim, Christian Donner, Michele Fiscella, Karsten Borgwardt, Verdon Taylor, Ravi Jagasia, Damian Roqueiro, Andreas Hierlemann, Manuel Schröter

**Affiliations:** 1 Department of Biosystems Science and Engineering, ETH Zürich, 4056 Basel, Switzerland; 2 Roche Pharma Research and Early Development, Neuroscience and Rare Diseases, Roche Innovation Center Basel, F. Hoffmann-La Roche, 4070 Basel, Switzerland; 3 Swiss Data Science Center, ETH Zürich, 8092 Zürich, Switzerland; 4 MaxWell Biosystems AG, 8047 Zürich, Switzerland; 5 Swiss Institute of Bioinformatics, 1015 Lausanne, Switzerland; 6 Department of Biomedicine, University of Basel, 4031 Basel, Switzerland

**Keywords:** electrophysiology, HD-MEA, toolbox, Parkinson's disease, alpha-synuclein

## Abstract

Reproducible functional assays to study *in vitro* neuronal networks represent an important cornerstone in the quest to develop physiologically relevant cellular models of human diseases. Here, we introduce *DeePhys*, a MATLAB-based analysis tool for data-driven functional phenotyping of *in vitro* neuronal cultures recorded by high-density microelectrode arrays. *DeePhys* is a modular workflow that offers a range of techniques to extract features from spike-sorted data, allowing for the examination of functional phenotypes both at the individual cell and network levels, as well as across development. In addition, *DeePhys* incorporates the capability to integrate novel features and to use machine-learning-assisted approaches, which facilitates a comprehensive evaluation of pharmacological interventions. To illustrate its practical application, we apply *DeePhys* to human induced pluripotent stem cell–derived dopaminergic neurons obtained from both patients and healthy individuals and showcase how *DeePhys* enables phenotypic screenings.

## Highlights

• *DeePhys* is a tool for electrophysiological phenotyping of *in vitro* neuronal cultures
• *DeePhys* allows for phenotypic screenings at the cellular and network levels
• *DeePhys* uncovers reliable features to predict healthy and mutant neuron lines
• *DeePhys* enables comprehensive evaluations of pharmacological interventions

## Teaser

Hornauer and colleagues introduce *DeePhys*, a novel analysis workbench for functional phenotyping of human neurons using high-density microelectrode array data. The authors apply *DeePhys* to iPSC-derived neuronal cultures obtained from healthy controls and patients. They demonstrate that *DeePhys* uncovers reliable features to predict healthy and mutant neuron lines. Moreover, they show how *DeePhys* enables a detailed characterization of pharmacological interventions.

## Introduction

Neurological disorders are a leading cause of disability in aging societies (Feigin et al., 2020). However, despite considerable efforts, our understanding of fundamental pathomechanisms has remained incomplete. Moreover, many therapeutic approaches failed in clinical trials, and treatment options for patients remain limited. To close the apparent translational gap, there is an urgent need for new preclinical assays that enable researchers to investigate disease mechanisms in human tissue at scale and to evaluate drug candidates more effectively.

With the advent of human induced pluripotent stem cell (iPSC) technology, it is now possible to generate neural cells in a reproducible and scalable manner from any patient or healthy individual. Human iPSCs hold great promise to uncover some of the mechanisms and developmental pathways that give rise to neurological disorders *in vitro* (Dolmetsch and Geschwind, 2011). However, in contrast to genomic or proteomic analysis readouts, there is currently no established framework for a standardized functional characterization of human neurons.

Functional assays obtained with high-density microelectrode arrays (HD-MEAs) have recently gained momentum (Abbott et al., 2020; Müller et al., 2015), since they allow for the capture of the electrical activity of neurons at scale (several hundreds of neurons per HD-MEA), across extended development time (several months), and at high temporal (10–20 kHz sampling rate) and spatial resolution (subcellular details). Moreover, new multiwell HD-MEA system designs provide the throughput needed for high-content screenings.

Although some large-scale electrophysiological systems to screen human neurons *in vitro* have become available (e.g., the 48-well plates by Axion Biosystems), suitable tools to analyze the rich extracellular data of today’s HD-MEAs (e.g., the 6-well plates by MaxWell Biosystems) have lagged behind. Most currently available toolboxes provide means to study either individual cells (Lee et al., 2021; Petersen et al., 2021) or network activity (Mahmud and Vassanelli, 2019), but they are often limited to certain aspects of the MEA data; for a review on currently available toolboxes, see Unakafova and Gail (2019).

Here, we introduce *DeePhys*, a novel analysis workbench that provides an integrated approach to extract and compare single-neuron and network-level features for functional phenotyping of human neurons. We outline the different processing modules of *DeePhys* and apply those in proof-of-concept experiments to iPSC-derived neuronal cultures obtained from healthy controls and patients with Parkinson disease (PD). While the primary application example of *DeePhys* is the functional phenotyping of iPSC-derived human dopaminergic (DA) neuron cultures harboring the A53T point mutation, we also probe how results can be generalized to other lines.

We first demonstrate that *DeePhys* uncovers reliable features to predict healthy and mutant DA neuron lines and their developmental stage. Moreover, our results indicate that the high-dimensional feature space allows for evaluating treatment interventions—here, exemplified by the application of locked nucleic acid (LNA)–mediated downregulation of α-syn expression. Finally, we show how *DeePhys* can be used to identify the phenotypes of more heterogeneous neuronal cultures and how acute drug challenges can be used to refine these results. We expect that the obtained insights will add an important layer to the characterization of human cellular models of neurological diseases *in vitro*.

## Results

### *DeePhys* processing modules

**Figure 1:**
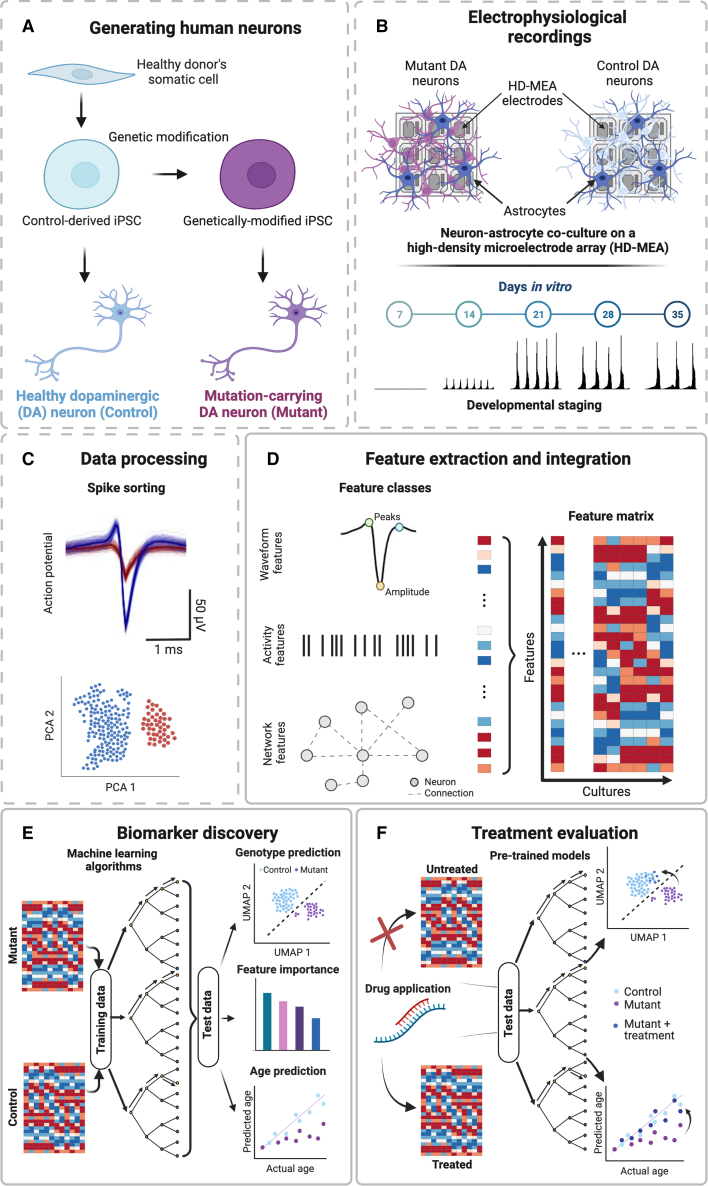
Schematic of the *DeePhys* analysis pipeline (A and B) Schematic outlining the generation of neurons from human iPSCs (A), and the plating and recording of human neurons on HD-MEAs (B). (C–F) The *DeePhys* pipeline starts with data that has been spike sorted (C) and consists of several modules: the HD-MEA feature extraction and integration (D), the feature evaluation (E), and the assessment of treatment interventions (F).

**Figure 2:**
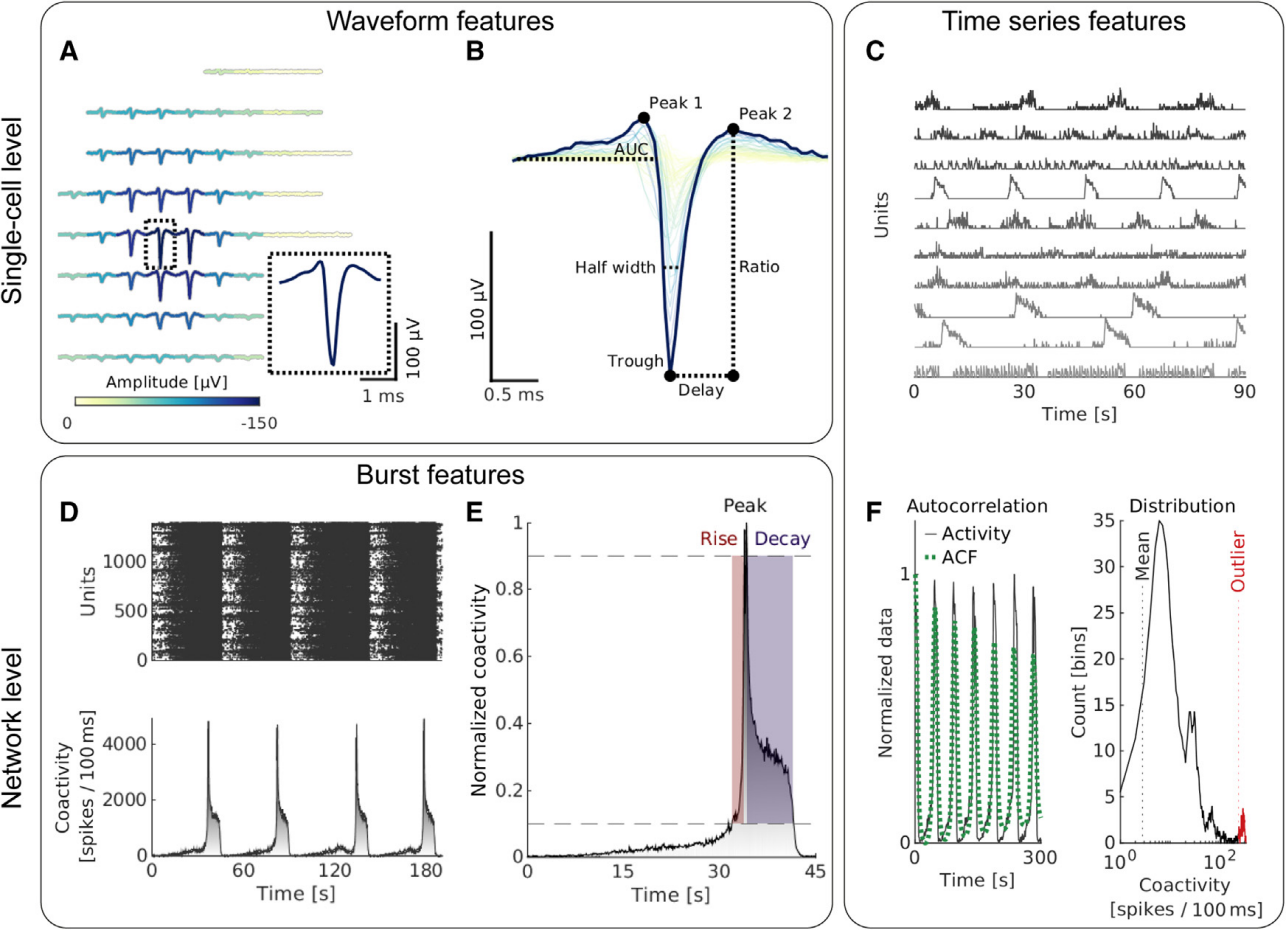
Quantifying neuronal activity on HD-MEAs Example features describing neuronal activity at the single-cell and network level. (A) The spike-triggered electrical footprint (EF) of a single unit on the HD-MEA. (B) The largest negative amplitude waveform of an EF was used to infer waveform features. The coloring indicates the respective trough amplitudes. (C) Binned spike trains of individual units were used for the time series feature extraction. (D) Raster plot with activity recorded from a DA neuronal culture at week 5. Dots represent spike-sorted action potentials. (Bottom) The corresponding binned coactivity of all of the units. (E) Magnification of a single network burst, highlighting its rise and decay phase. (F) Examples of time series features, such as the autocorrelation function (ACF) and the distribution of the binned coactivity, as inferred from single-cell (C) and network (D) activities.

**Figure 3:**
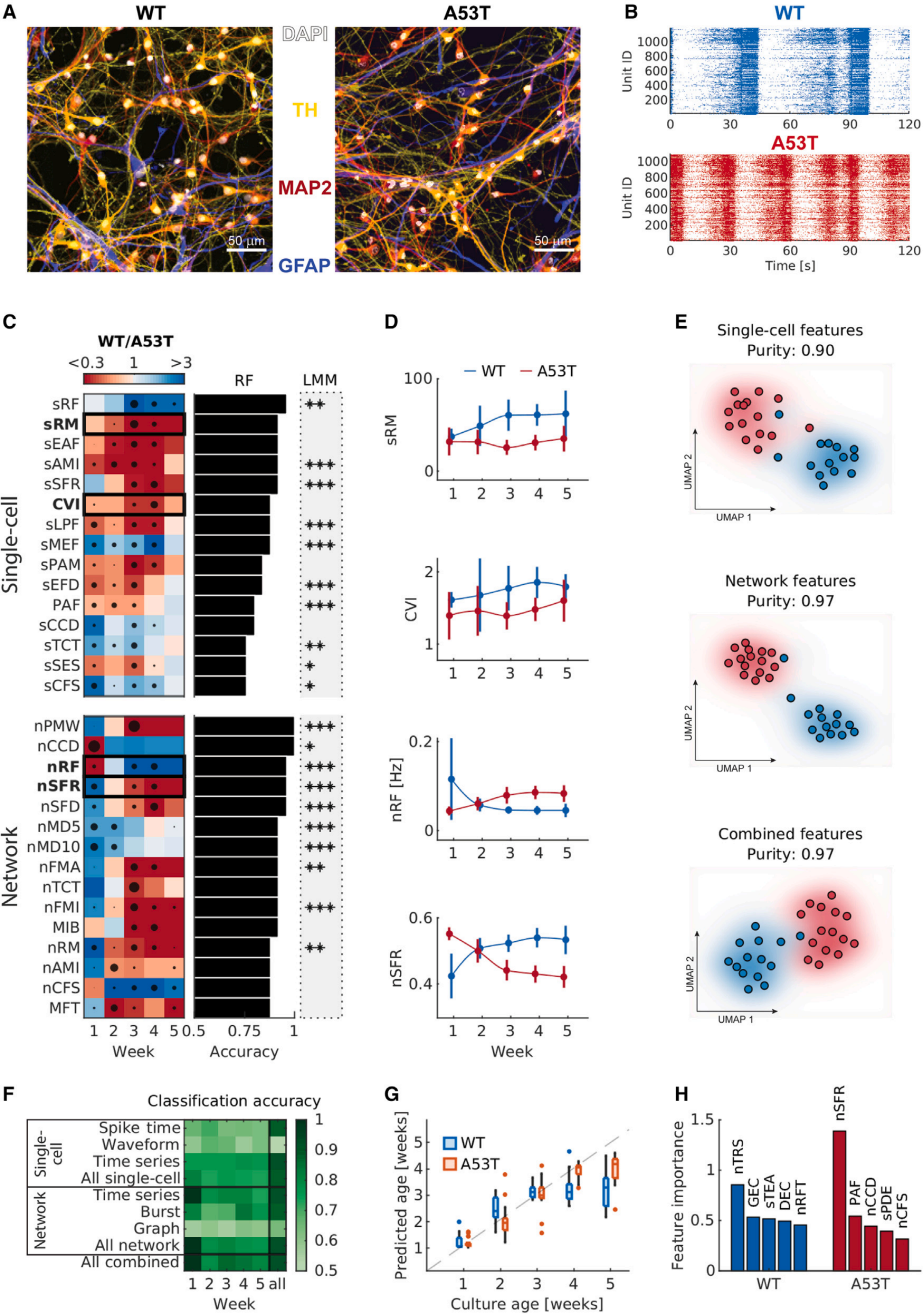
A53T DA neuron cultures exhibit age-dependent alterations at the single-cell and network level (A) ICC staining of WT (left) and A53T (right) human iPSC-derived DA neurons, cocultured with human astrocytes at week 3. See also Figure S1. (B) Example raster plot with activity recorded from a WT and an A53T DA neuronal culture at week 5. (C) Heatmaps indicating differences between genotypes at a given recording time point (top: single-cell features, bottom: network features). The horizontal bar plots display the accuracies of RF classification models, trained with the respective features as input. The size of the black dots within each panel of the heatmap indicates the relative predictor importance at the respective time point. Asterisks indicate the significance levels of the corresponding LMMs (^∗^p < 0.05, ^∗∗^p < 0.01, ^∗∗∗^p < 0.001; N_WT_ = 18, N_A53T_ = 19 cultures). See Table 1 for more detailed feature descriptions. (D) Example developmental trajectories of 4 highly predictive features (mean ± SD values; N_WT_ = 18, N_A53T_ = 19 cultures). (E) UMAP dimensionality reduction, based on either single-cell or network features, or a combination of both feature classes, demonstrates the separability of WT and A53T cultures. The cluster purity values quantify the result of k-means clustering analyses (k = 2). (F) Heatmap depicting the RF classification accuracy by culture age and input feature group (N_WT_ = 14, N_A53T_ = 15 cultures). (G) Age prediction using RF regression analysis (boxes show the median, lower, and upper quartiles; whiskers indicate the nonoutlier minimum and maximum values; dots indicate outliers). (H) Bar graph depicting the features with the highest predictor importance inferred from the RF age prediction. See Table 1 for more detailed feature descriptions.

**Figure 4:**
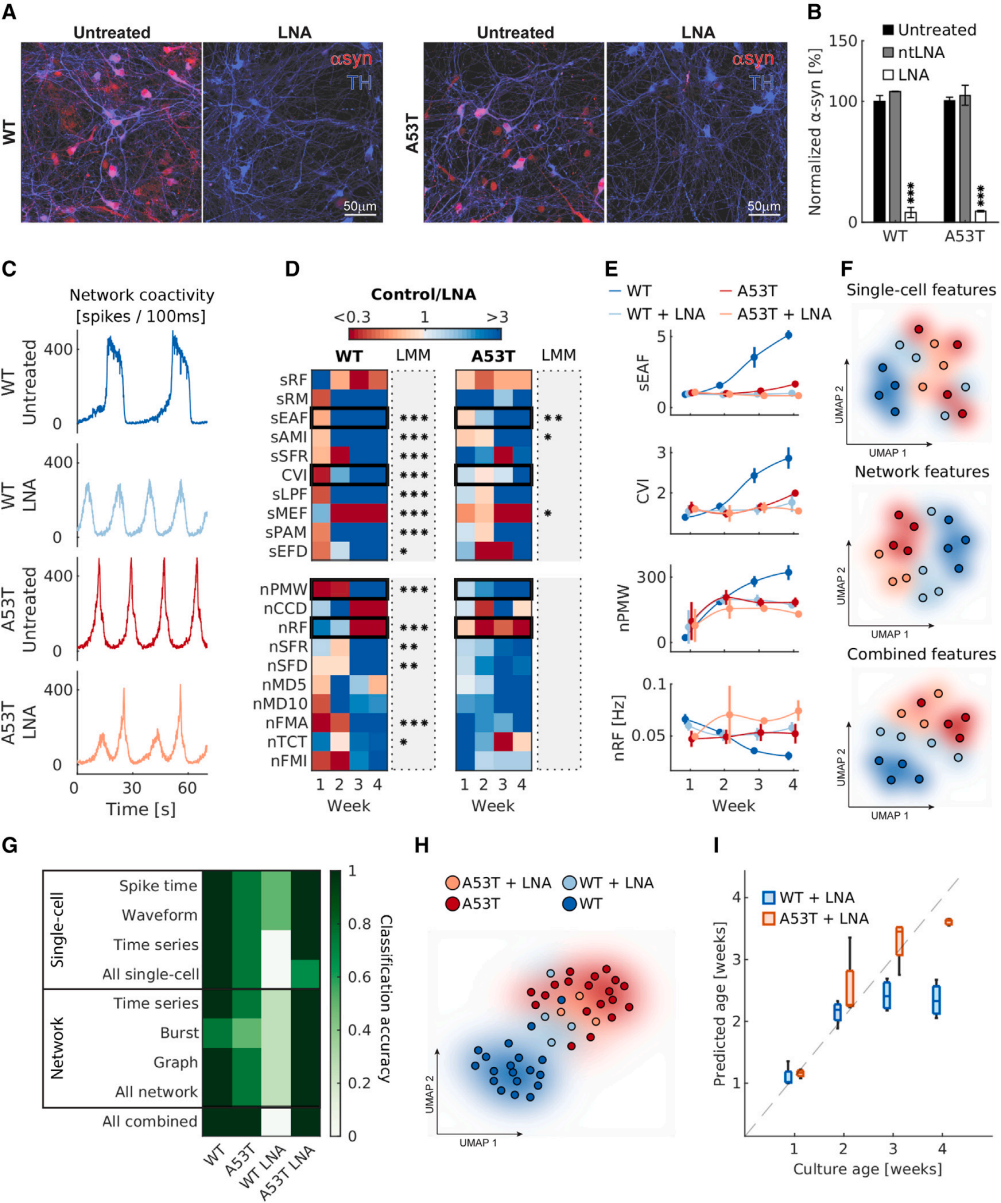
Downregulation of α-syn alters electrophysiological phenotypes and culture development (A) Representative staining for α-syn and TH of WT/A53T DA neuronal cultures at week 3. (B) Quantification of α-syn levels using an HTRF assay. Values are normalized by untreated WT levels (Tukey-Kramer test, ^∗∗∗^p < 0.001; N = 3 cultures with 3 technical replicates). (C) Representative coactivity plot of LNA-treated and untreated WT/A53T DA neuronal cultures at week 5. (D) Heatmaps depicting the relative feature differences of the 10 most predictive single-cell (top) and network features (bottom) as shown in Figure 3C. Asterisks indicate the significance of the respective LMMs (^∗^p < 0.05, ^∗∗^p < 0.01, ^∗∗∗^p < 0.001; N = 20 cultures). (E) Exemplary developmental trajectories of 4 highly predictive features (mean ± SD values). (F) UMAP dimensionality reduction, based on either single-cell or network features, or a combination of both feature classes. (G) Heatmap depicting the RF classification accuracy of control cultures (N_WT_ = 4, N_A53T_ = 5) and LNA-treated cultures (N_WT_ = 4, N_A53T_ = 3) by input feature group. The RF classifier was trained on all of the cultures of the experiment displayed in Figure 3. (H) Projection of LNA-treated cultures into the UMAP space of untreated cultures. (I) Age prediction of LNA-treated cultures using an RF regression model trained on untreated cultures (boxes visualize the median, lower, and upper quartiles; whiskers indicate nonoutlier minimum and maximum values; dots indicate outliers).

**Figure 5:**
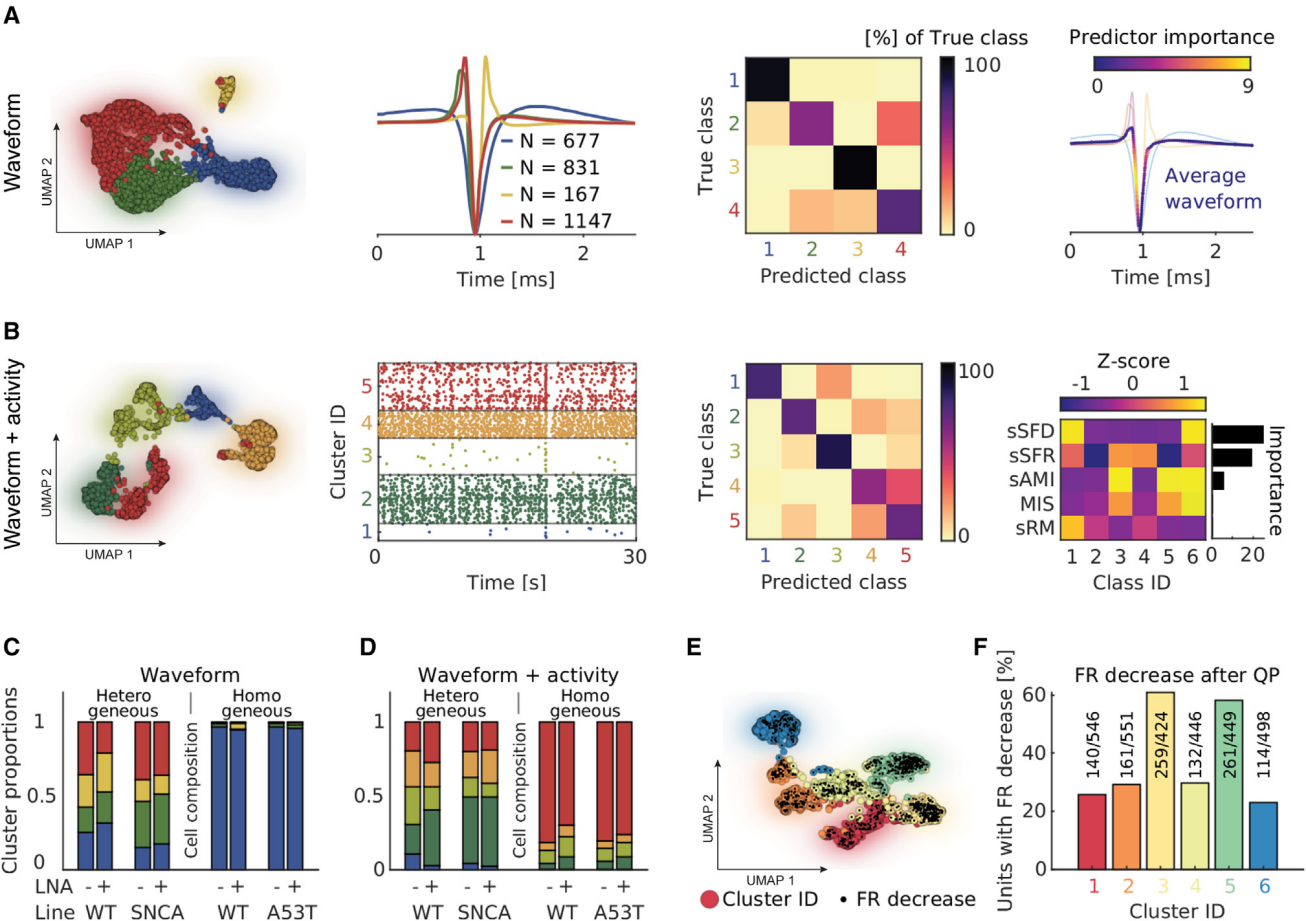
Characterizing electrophysiological phenotypes at the single-cell level Inferring reliable single-cell clusters in heterogeneous cultures (N = 5 cultures) based on action potential waveforms (A) or a combination of action potential waveforms and spike activity (B). From left to right: the UMAP embeddings (colors indicate the cluster ID from Louvain clustering); the average waveforms (A) and representative spike trains (B) of the corresponding clusters; the confusion matrices of RF classifiers trained on the inferred clusters; and the predictor importance values of waveform- and activity-based classifiers. (C) Predicted cluster compositions using the classifier from (A). (D) Predicted cluster compositions using the classifier from (B). (E) UMAP plot of the single-cell baseline activity; colors indicate the cluster ID from Louvain clustering. Black dots indicate a unit with a reduction in FR upon QP addition (N = 2,914 units from 5 cultures). (F) Ratio of units that display a FR decrease upon QP addition.

*DeePhys* is composed of four analysis modules. First is a preprocessing module, which allows for quality control of the spike-sorted input data and the extraction of a large range of extracellular features (Figure 2). Second is a feature-integration and phenotyping module that combines the inferred extracellular features at individual time points or across development to derive robust electrophysiological phenotypes (Figure 3). This module relies on machine learning algorithms to probe the reliability of the inferred phenotypes and identifies the most predictive features, which can be used to find potential biomarkers. The third module provides means to assess the impact of acute or chronic pharmacological interventions on the inferred phenotypes (Figure 4). Finally, the fourth module enables feature-based single-cell clustering for analyses at the level of putative cell types (Figure 5). A schematic overview of the *DeePhys* pipeline is given in Figure 1, and examples of HD-MEA inferred single-neuron and network-level features are shown in Figure 2 (see Table S1 for a full list). The main functions are listed in Table S2. The MATLAB code to run *DeePhys* is open source and available on a public code repository (see Experimental procedures), where we also provide online tutorials and test data.

### Inferring extracellular electrophysiological features

We previously demonstrated the feasibility of recording from human neurons using HD-MEAs and of profiling them using standard spike-train statistics (Ronchi et al., 2021). Here, we significantly expanded the range of features and derived >50 quantitative measures from spike-sorted HD-MEA recordings. Following a quality control step to assess the spike-sorting results, we inferred two main classes of features: single-cell metrics and network-level metrics (Figure 2). Briefly, the single-cell metrics consisted of eight action potential waveform metrics (later referred to as waveform features; Figures 2A and 2B) and four metrics that describe spike-train dynamics (spike time features). There is a large body of evidence that waveform and spike time metrics are indicative of putative cell types (Trainito et al., 2019) and their laminar location *in vivo* (Lee et al., 2021).

At the network level, we quantified functional-connectivity features (graph features) and the intrinsic network-burst activity (burst features; Figures 2D and 2E). Previous studies have linked differences in these features to synaptic functioning and, more generally, network maturation (Wagenaar et al., 2006). Finally, we also derived time series features, which were inferred from the binned spiking activity of individual units as well as the whole network (Figures 2C and 2F). These features were obtained in a previous study (Lubba et al., 2019) and have been used for time series classification tasks (Fulcher and Jones, 2017). We hypothesized that applying and combining single-cell and network-level features would facilitate the functional phenotyping of neuronal networks.

### Characterizing human iPSC-derived midbrain DA neuronal networks

As a proof of principle for the *DeePhys* toolbox, we used two human cell lines of commercially available, purified, and fully differentiated midbrain DA neurons. The protocol to differentiate these DA neurons from iPSCs was based on work by the Studer lab (Kriks et al., 2011). The two lines comprised a healthy control line and an isogenic mutant line, with the A53T site-specific mutation introduced into the *SNCA* gene. The A53T variant has one of the strongest effects on the initiation and spreading of α-syn aggregations (Flagmeier et al., 2016) and results in an autosomal dominant form of familial PD (Polymeropoulos et al., 1997). Both lines were cocultured with human iPSC-derived astrocytes and plated on HD-MEAs, as described previously (Ronchi et al., 2021). Weekly HD-MEA recordings of the emerging neuronal activity started 7 days after the plating. Figure 2B shows representative network-activity plots for both WT and A53T DA neuron cultures.

Using immunocytochemical (ICC) analysis (N = 3 cultures per cell line), we confirmed the presence of DA neurons and astrocytes in control experiments by staining for tyrosine hydroxylase (TH), microtubule-associated protein 2 (MAP2), and glial fibrillary acidic protein (GFAP) at day *in vitro* 21 (Figures 2A and S1). Both wild-type (WT) and A53T cultures showed robust outgrowth and neuritic arborization of TH^+^ neurons and a wide coverage of GFAP^+^ astrocytic processes. Quantifications of TH^+^ and MAP2^+^ nuclei revealed subtle differences in the composition of both lines (Table S3). A comparison of α-syn levels between genotypes is provided in the section Assessment of pharmacological perturbations.

### *DeePhys* allows accurate predictions of DA neuron culture type and age

Following the quality control and feature extraction steps, we trained random forest (RF) classifiers for each metric. The resulting matrix (N_cultures_ × N_recordings_) was then normalized and used as input to predict the culture type (WT versus A53T); leave-one-out cross-validation (CV) was applied to obtain accuracy values for each feature. In addition, we calculated the permutation predictor importance to infer the relative predictor importance at each recording time point (i.e., the week *in vitro*). Statistical differences in the development of features were assessed by linear mixed-effects models (LMMs). Table 1 provides an overview of the features used throughout the paper.

**Table 1:** Feature names and descriptions

Feature	Feature class	Description
MIB	Burst	Mean interburst interval
MFT	Burst	Mean time from the peak to the end of a burst
GEC	Graph	Global efficiency calculated on cross-correlogram (CCG)-inferred connectivity graph
DEC	Graph	Network density based on CCG graph
CVI	Spike time	Coefficient of variation of the interspike interval
MIS	Spike time	Mean interspike interval
RF	Time series	Peak frequency of Fourier power spectrum
RM	Time series	Magnitude of the corresponding RF feature
EAF	Time series	First 1/e crossing of autocorrelation function
AMI	Time series	Automutual information
SFR	Time series	Proportion of slower timescale fluctuations that scale with linearly rescaled range fits
LPF	Time series	Total power in the lowest fifth of frequencies in the Fourier power spectrum
MEF	Time series	Mean error from a rolling 3-sample mean forecasting
PAM	Time series	Longest period of consecutive values above the mean
EFD	Time series	Exponential fit to successive distances in two-dimensional embedding space
CCD	Time series	Change in correlation length after iterative differencing
TCT	Time series	Trace of covariance of transition matrix
SES	Time series	Shannon entropy
CFS	Time series	Centroid of the Fourier power spectrum
SFD	Time series	Proportion of slower timescale fluctuations that scale with detrended fluctuation analysis
MD5	Time series	Mode of *Z* scored distribution (5-bin histogram)
MD10	Time series	Mode of *Z* scored distribution (10-bin histogram)
FMA	Time series	First minimum of autocorrelation function
FMI	Time series	First minimum of the automutual information function
TRS	Time series	Time-reversibility statistic
TEA	Time series	Time intervals between successive extreme events above the mean
RFT	Time series	Exponential fit of successive peak frequencies in the Fourier power spectrum
PDE	Time series	Proportion of successive differences exceeding 0.04 SD

Subset of *DeePhys* features and their respective abbreviations as used throughout the paper. Time series features that were inferred at the single-cell or the network level are denoted by a leading s or n, respectively (e.g., sRM refers to the single-cell regularity magnitude). See Table S1 for a complete list of features.

We found that WT and A53T DA neuron cultures showed marked differences across single-cell and network-level metrics (Figure 3). Single-cell features differed consistently between genotypes at the activity level, particularly metrics that describe the temporal regularity of DA neuron firing. The single-cell regularity frequency (accuracy: 0.96, p < 0.01, LMM) and regularity magnitude (accuracy: 0.92, p > 0.05, LMM) were the two most predictive features among the time series feature group (Figure 3D). Spike time features, such as the coefficient of variation of the interspike interval (CVI, accuracy: 0.92, p > 0.05, LMM), were also highly predictive (Figure 3D). Waveform features were less predictive for the classification of cell lines (maximum accuracy value of 0.68).

Network-level features also showed clearly distinct phenotypic profiles for WT and A53T cultures (Figure 3C, lower panel), which became apparent early during development. Although A53T cultures featured high levels of correlated spontaneous activity 1 week after plating, no bursts were detected in WT cultures at that time. The time series feature group was most predictive at the network level (e.g., periodicity measure, accuracy: 1.00, p < 0.01, LMM). Various network-level burst metrics were also highly predictive, reaching accuracy values of up to 0.92 (e.g., the mean interburst interval [MIB], p > 0.05, LMM). The waveform and graph-feature groups showed the lowest accuracy across development (accuracy <0.58 and <0.60; Figure 3F). The network-feature group displayed a reduction in accuracy in week 2, consistent with a crossover of some developmental feature trajectories around that time. The aggregation of features across development improved the classification performance (Figure 3F).

Uniform manifold approximation and projection (UMAP) analyses of single-cell or network-level features, or a combination of both feature classes, also indicated robust differences between WT and A53T cultures (Figure 3E). Visual inspection allowed a clear distinction of both lines in the two-dimensional (2D) UMAP space. This result was corroborated by high cluster purity values (separability of clusters) for both network-level (0.97) and single-cell features (0.90); combining network and single-cell features did not further improve the separability (0.97).

Finally, we used RF regression models to probe whether features inferred by the *DeePhys* pipeline would let us predict the age of DA neuron cultures (Figure 3G). We found that age predictions remained accurate until week 3 for WT cultures (mean average error [MAE] = 2.4 days). After 3 weeks *in vitro*, the predicted age plateaued (MAE = 9.0 days). The age of A53T cultures was accurately predicted until week 4 (MAE = 2.3 days), but then underestimated at week 5 (MAE = 4.3 days). We found that the features driving the age predictions differed significantly between WT and A53T cultures (Figure 3H). Together, our results show that the *DeePhys* pipeline allowed for robust functional phenotyping of WT/A53T DA cultures based on features extracted from spike-sorted HD-MEA recordings.

### Assessment of pharmacological perturbations

Next, we used *DeePhys* to map out the effects of a pharmacological intervention on WT/A53T functional phenotypes. Specifically, we studied the alterations following chronic application of a LNA to reduce α-syn expression. Although reducing α-syn has been discussed as a treatment option for PD (Fields et al., 2019), the main goal of the current experiment was to monitor the effect of such a reduction on the electrophysiological profiles obtained by *DeePhys*.

We confirmed the efficacy of the LNA construct by measuring α-syn levels in WT/A53T cultures that were either untreated, treated with a nontargeted LNA (ntLNA), or treated with an *SNCA*-targeting LNA. This control experiment was performed on coverslips at week 3 using a homogeneous time resolved fluorescence (HTRF) assay (N = 3 cultures per condition; Table S4). Results indicated a significant effect of the LNA treatment on total α-syn levels (two-factor ANOVA, p < 0.001), but no significant difference between genotypes (p = 0.820). Post hoc multiple comparisons tests (Tukey-Kramer test) showed a significant decrease in α-syn in LNA-treated WT and A53T cultures compared to untreated controls (both p < 0.001; Figure 4B); α-syn levels in cultures treated with ntLNA did not differ significantly from levels obtained in untreated controls (p > 0.999). ICC analysis (N = 3 cultures per condition) of somatic α-syn levels confirmed these results (two-factor ANOVA, treatment: p < 0.001, genotype: p = 0.116; Table S5). Phosphorylated α-syn (*p*-syn) levels, however, were significantly increased in A53T DA neurons (two-factor ANOVA, p = 0.001; Table S6).

We then applied the *DeePhys* pipeline and asked whether downregulating α-syn via LNA treatment altered the electrical activity of DA cultures (Figure 4C). We focused our analysis on the 10 single-cell and network-level features with the highest predictive power in distinguishing between WT and A53T (see Figure 3C). For this analysis, we pooled untreated and ntLNA-treated cultures to increase statistical power (see Figure S2 for a comparison of ntLNA-treated and untreated culture profiles). Our results indicate that LNA-mediated α-syn downregulation led to distinct alterations, such as shorter but more frequent bursts. Moreover, while the network regularity frequency (nRF) typically decreased throughout development in WT cultures, LNA treatment reversed this trend (Figure 4E, lowest panel). A53T cultures exhibited a similar development, as LNA treatment increased the nRF metric. Most metrics showed similar trends in both lines, but LNA-induced differences were predominantly significant in WT cultures (Figure 4D). UMAP analyses visualized the robustness of the LNA effect because LNA-treated WT cultures mostly colocalized with A53T cultures across feature classes (Figure 4F).

We then applied the RF classifier trained on the cultures of our previous experiment (Figure 3) to the cultures of the LNA experiment. Results for predicting the phenotype of untreated WT and A53T cultures indicated that most feature groups generalized very well (Figure 4G), and even perfect classification was achieved by combining all of the feature classes (“all combined”). This validation allowed us to assess the effect of the LNA treatment by calculating the classification accuracy of LNA-treated WT and A53T cultures. The accuracy of the prediction of LNA-treated WT cultures was low across all of the feature groups, but remained high for LNA-treated A53T cultures (Figure 4G, WT LNA+A53T LNA). This observation is consistent with the UMAP results (Figure 4H) because LNA-treated WT cultures colocalized visually with untreated A53T cultures from the first experiment (see results from Figure 3).

Finally, we assessed the impact of α-syn downregulation on overall culture development by predicting the age of LNA-treated cultures using the RF regression models trained on their respective controls. Results indicated that the development of LNA-treated WT cultures plateaued earlier (after 2 weeks *in vitro*), whereas A53T cultures remained largely unaffected by the LNA treatment (Figure 4I).

### Applying *DeePhys* to heterogeneous human DA neuron cultures

Many of the current neuron differentiation protocols give rise to heterogeneous cultures (i.e., a diverse set of neuron types per culture), which may increase the variability between cell lines and affect functional phenotyping (Volpato et al., 2018).

Here, we set out to probe whether *DeePhys* could be used to identify electrophysiological phenotypes at the cellular level and to infer putative cell clusters. We therefore recorded from two heterogeneous lines obtained from differentiated iPSC-derived midbrain floor plate progenitors: a cell line obtained from a healthy control subject and another from a PD patient with the *SNCA* triplication (SNCA). Cultures were prepared using a previously published protocol with a proportion of DA neurons of approximately 8% at week 3 (Fedele et al., 2017).

Results obtained with *DeePhys* indicated clear differences between homogeneous and heterogeneous cell lines, as well as between healthy controls and PD-associated mutations (A53T, SNCA; Figure S3). Given these differences, we next asked whether single-cell HD-MEA features could be used to parse out the different cellular compositions of homogeneous or heterogeneous cultures. We performed single-cell clustering on heterogeneous control cultures (Louvain clustering), using either action potential waveforms (Figure 5A) or a combination of waveforms and activity features (Figure 5B). An RF classifier was then trained on these clusters and used to quantify the importance of individual features (Figures 5A and 5B, panels at right) and to infer putative cell type compositions of the cultures (Figures 5C and 5D). We found that all of the clusters were present among the spike-sorted units of the heterogeneous cell lines, and one dominating cluster consistently made up >70% of all of the units in the homogeneous lines, irrespective of the genotype or treatment condition. These results indicate that *DeePhys* enabled reliable cell-cluster assignments despite pronounced differences at the network level (Figures 4C and S3).

Finally, we probed whether an acute pharmacological perturbation could be used to further refine the single-cell clustering. Previous research has demonstrated that the dopamine receptor D2 agonist quinpirole (QP) reduces DA neuron firing rates (FRs), so we expected cell cluster–specific responses after QP addition (Dagra et al., 2021). We therefore performed Louvain clustering on the UMAP graph generated from heterogeneous, untreated WT baseline recordings and mapped the FR change after QP (10 μM) treatment (Figure 5E; an FR decrease is marked with a black dot). Units in clusters 3 and 5 predominantly decreased their FR (60% of the units in these clusters), whereas in all of the other clusters such a decrease was only observed for approximately 30% of the units (Figure 5F). Some of these clusters persisted when analyzing the QP response, indicating a correlation between baseline activity and QP response (Figure S4). Moreover, results indicated that the obtained QP response clusters may facilitate the phenotyping of heterogeneous cell lines (Figure S4).

## Discussion

The main goal of this study was to introduce and validate *DeePhys*, a new open source analysis pipeline to extract multiparametric information from spike-sorted electrophysiological recordings. The *DeePhys* pipeline is modular and easily scalable, it requires only minimal manual intervention, and it can be used as a screening tool to investigate different human cellular models. We applied *DeePhys* to purified and heterogeneous human iPSC-derived DA neuronal cultures, maintained over several weeks on HD-MEAs, and demonstrated its utility in systematically studying neuronal maturation and pharmacological perturbations.

For the first experiment, we used the *DeePhys* pipeline to identify descriptive phenotypic differences between A53T mutant and isogenic control DA neuron cultures across development. We found that both cell lines can be reliably classified according to their electrophysiological phenotypes, with network-level metrics representing the more informative features. Moreover, changes related to the regularity of neuronal activity were reliably detected in both single-cell and network features. A53T cultures showed an earlier onset of spontaneous synchronized activity and smaller bursts at a higher rate later in development. The differences in burst dynamics could provide a link to a previous study, which found that mutant α-syn interferes with vesicle recycling and the regulation of the recycling pool (Xu et al., 2016). Both processes are essential in maintaining synaptic activity over prolonged periods. Alterations in the functionality of α-syn due to the A53T mutation may therefore alter the duration and frequency of network bursts. Also, mitochondrial defects and bioenergetic deficits, caused by the A53T mutation, may reduce the length of high-activity periods because the energy demand of prolonged spiking cannot be met (Ryan et al., 2013; Zambon et al., 2019).

Next, we showed how *DeePhys* can be used to assess the impact of chronic pharmacological interventions on the functional phenotypes. The strongly decreased α-syn levels in DA neurons after LNA treatment resulted in an electrophysiological phenotype similar to that of A53T mutant cultures. This finding was confirmed by applying a pretrained RF model on LNA-treated cultures, which classified all LNA-treated cultures as A53T. However, most phenotypical features, such as an increased burst rate, were even more pronounced for the LNA condition. This result proved true regardless of the genotype, because WT and A53T cultures were affected similarly by the LNA-induced reduction in α-syn levels. α-Syn has been reported to be involved in the maintenance of the synaptic vesicle (SV) pool size through vesicle recycling and inhibition of inter-SV trafficking (Scott and Roy, 2012; Sun et al., 2019). In addition, α-syn was shown to cluster SVs and to attenuate the release of neurotransmitters (Wang et al., 2014). A reduction in α-syn levels may, therefore, restrict the SV pool size before bursts and the recycling rate during network bursts, which could result in shorter, low-frequency bursts.

Furthermore, we demonstrated how *DeePhys* can be used to bridge the gap between single-cell and network phenotypes by extracting patterns from the action potential waveform shape and activity of individual cells. We found that distinct unit clusters could be detected for iPSC-derived heterogeneous neuronal lines (WT, *SNCA* triplication), whereas one cluster was consistently dominant for the DA neuron–enriched homogeneous lines. Interestingly, the putative cell cluster composition was consistent across cell lines and treatment conditions, despite pronounced differences in their network activity. Although this finding may indicate that the predominant cluster corresponds to the DA neuron population of the more homogeneous cultures, this will have to be probed in future studies with ground-truth data. Such validation seems necessary, as the spike waveform of a unit varies not only by its cell type but also by the location and the distance and angle of the cell to the respective HD-MEA electrodes.

Lastly, we showed how *DeePhys* can be used in combination with acute pharmacological perturbations (QP) to detect differential responses of individual unit clusters. In line with previous research (Dagra et al., 2021), we found clusters that predominantly reduced in FR after QP application. In addition, we found that baseline activity clusters could be partially mapped to the respective QP responses, and that the resulting clusters could be used to characterize the cell line–specific QP response across the whole culture. In future studies, this approach could be expanded by incorporating more perturbation responses or other modalities, such as RNA sequencing data, to further validate the inferred clusters.

Our results show that *DeePhys* provides an easy-to-use, scalable, quantitative analysis platform for functional phenotype screening and for addressing important biomedical questions in the development of new treatments. Its compatibility with SpikeInterface (Buccino et al., 2020) allows *DeePhys* to be used with most popular spike-sorting algorithms, and its modular organization facilitates the integration of new input formats and the addition of other feature groups (e.g., local field potential data). We are confident that *DeePhys* and the analysis approach presented here have great potential to add to a better functional characterization of a wide range of cellular models of neurological diseases.

## Experimental procedures

### Resource availability

#### Corresponding author

Further information and requests for resources and reagents should be directed to and will be fulfilled by the corresponding author, Philipp Hornauer (philipp.hornauer@bsse.ethz.ch).

#### Materials availability

This study did not generate new unique reagents.

#### Data and code availability

The code to run *DeePhys* and to reproduce the figures is available at https://github.com/hornauerp/DeePhys.git. The raw datasets have not been deposited in a public repository due to the excessive file size (>5 TB) but are available from the corresponding author upon reasonable request. The preprocessed datasets are available at https://doi.org/10.5281/zenodo.7876371.

### Cell lines

#### Homogeneous cultures

Fully differentiated human iPSC-derived DA neurons carrying a heterozygous A53T mutation (C1112, FUJIFILM Cellular Dynamics International, Madison, WI) and an isogenic control line (C1087) were cocultured with astrocytes (R1092) as previously described (Ronchi et al., 2021).

#### Heterogeneous cultures

Human iPSC lines from apparently healthy controls and PD patients carrying a triplication of the *SNCA* gene were differentiated using a recently established protocol (Fedele et al., 2017). On day 20 of differentiation, cultures were dissociated, and 120,000 cells were plated onto each HD-MEA.

All medium formulations and methods for generation, plating, and culture of iPSC-derived DA neurons, ICC (see Table S9 for a list of antibodies), and image analysis are detailed in the supplemental experimental procedures.

### HD-MEA recordings

Neuronal cultures were recorded for 15 min weekly, as previously described (Ronchi et al., 2021), using the HD-MEAs MaxOne and MaxTwo (MaxWell Biosystems, Zurich, Switzerland). The used HD-MEAs feature 26,400 electrodes with a 17.5-μm pitch, 1,024 readout channels, and a 20-kHz (MaxOne)/10-kHz (MaxTwo) sampling rate (Müller et al., 2015). Electrode selection was performed based on an activity scan, and the electrodes with the highest FR were selected for the network scan. Spike sorting was performed using Kilosort 2.5 (Stringer et al., 2019) using parameters tailored to the dataset at hand (Table S7). The spike-sorted data underwent quality control, considering the overall activity of units (>0.01 Hz), the refractory period violations (<2%), and irregularities in the waveform shape. The experiments involving LNA and QP are detailed in the supplemental experimental procedures.

### Feature extraction

#### Single-cell features

Single-cell features were extracted from all of the units and then averaged to obtain one representative value for each culture (Figures 3 and 4). The action-potential waveform features were derived from the spike-triggered multielectrode waveform signal (template), as generated during the spike sorting (Figure 2A). Single-cell waveform features were extracted from the electrode with the largest negative waveform amplitude (Figure 2B).

The activity features included spike time features, which were inferred from the spike times of individual units. Time series features (Figure 2C) were inferred from the binned activity (bin size: 100 ms). Most time series features were adapted from a recent publication on time series classification (*catch22*, version 0.4.0; Lubba et al., 2019).

#### Network features

The network features describe the activity of the entire network after aggregating the activity of all of the spike-sorted units of a culture (Figures 2D and 2E).

To infer burst features, we detected network activity bursts using a previously introduced method (Bakkum et al., 2014). Since cultures were tracked across development, we adapted the algorithm to account for changes in overall activity.

Graph features were inferred from functional connectivity graphs that were calculated using either a cross-correlogram-based approach (English et al., 2017) or the spike time tiling coefficient (Cutts and Eglen, 2014).

Network time series features were calculated from the binned activity (bin size: 100 ms) across all of the spike-sorted units.

### Statistical analysis

We applied LMMs models of the form Y ∼ 1 + Genotype × Time + (1|Subject) to compute statistical significance in the developmental trajectories of individual features. We concatenated all of the feature values across recording time points for each culture. The restricted maximum likelihood (REML) estimation was used as a fitting method (MATLAB function: *lme = fitlme(input_table, formula, 'FitMethod','REML', 'DummyVarCoding', 'effects')*). The Satterthwaite approximation was applied to compute approximate degrees of freedom (MATLAB function: *anova(lme,'DFMethod','satterthwaite')*). The resulting p values were adjusted for the number of comparisons/features using the Bonferroni correction.

### Machine learning methods

The input matrix for the RF classifier was obtained by concatenating individual features (N_cultures_ × N_recordings_) or all of the features of a feature group across the selected recording time points (N_cultures_ × (N_recordings_ × N_features_)). The training data for the classification was batchwise transformed into *Z* scores to minimize interbatch variability; test data were transformed using parameters derived only from the training data. The model was implemented using the MATLAB function *fitcensemble and templateTree* learners. Accuracy values were obtained using a leave-one-out CV for network predictions (Figures 3 and 4) and a 5-fold CV for single-cell predictions (Figure 5). Hyperparameter optimization was performed on the training set using a 5-fold nested CV and 100 iterations of Bayesian optimization (Table S8). RF was selected for the analysis because it provides the option to infer predictor importance values (MATLAB function: *oobPermutedPredictorImportance*). The implementations of the clustering and the age regression analysis are detailed in the supplemental experimental procedures.

### HTRF assay

The HTRF assay (6FNSYPEG, Cisbio Bioassays, Codolet, France) was performed according to the manufacturer’s instructions; a detailed protocol is provided in the supplemental experimental procedures.

## Supplemental information

Document S1. Supplemental experimental procedures, Figures S1–S4, and Tables S1–S9

Document S2. Article plus supplemental information
