# Supplementary material for: DeePhys: A machine learning–assisted platform for electrophysiological phenotyping of human neuronal networks
Source: Stem Cell Reports. 2024 Jan 25;19(2):285–98. doi: 10.1016/j.stemcr.2023.12.008 (PMC10874850; doi:10.1016/j.stemcr.2023.12.008)
Supplement: Document S1. Supplemental experimental procedures, Figures S1–S4, and Tables S1–S9 [file mmc1.pdf]

Stem Cell Reports, Volume *19*

Supplemental Information

DeePhys: A machine learning–assisted platform for electrophysiological phenotyping of human neuronal networks

Philipp Hornauer, Gustavo Prack, Nadia Anastasi, Silvia Ronchi, Taehoon Kim, Christian Donner, Michele Fiscella, Karsten Borgwardt, Verdon Taylor, Ravi Jagasia, Damian Roqueiro, Andreas Hierlemann, and Manuel Schröter

Supplemental items

Supplemental figures

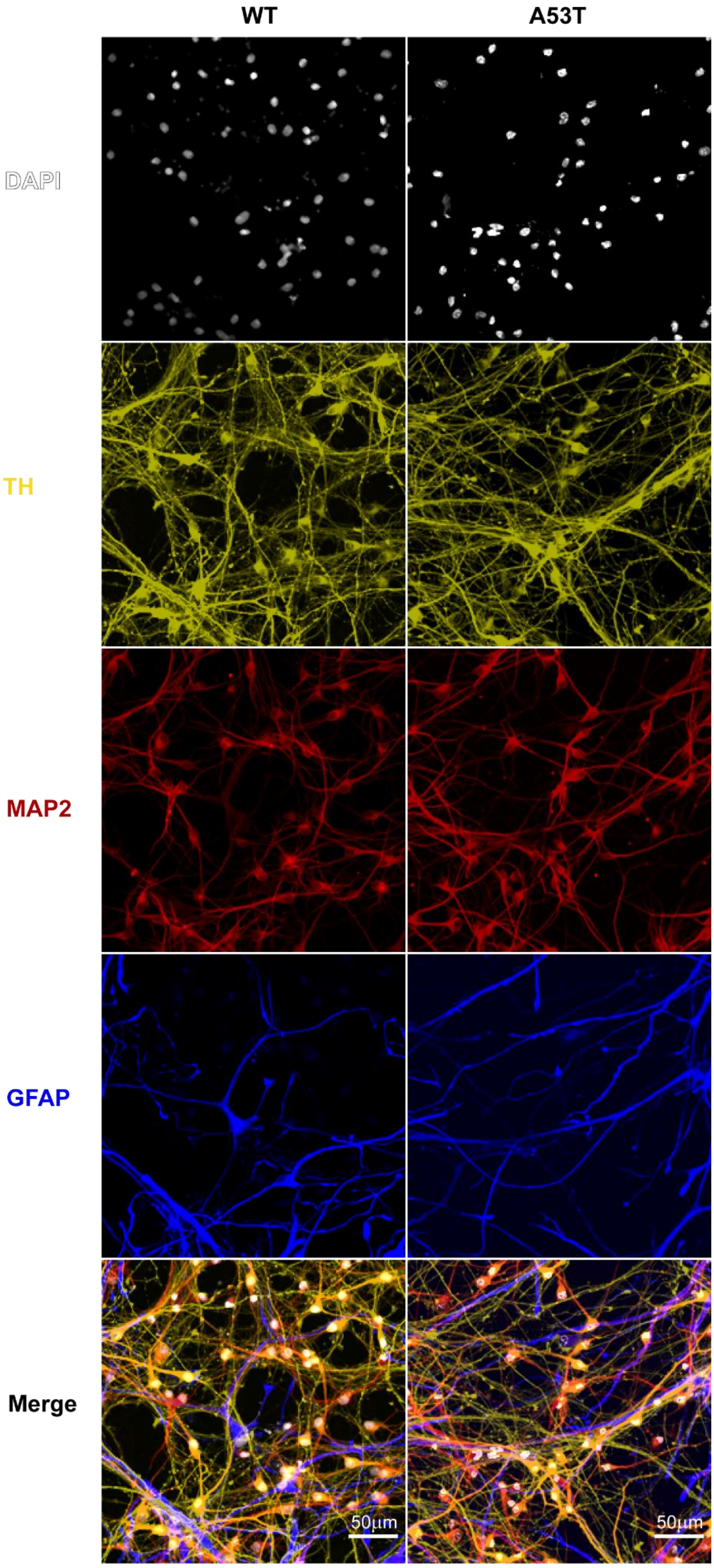

Figure S1. Immuno-cytochemical stainings of WT and A53T co-cultures. DA neuron-astrocyte co-cultures of both genotypes expressed MAP2+ (red) and TH+ (yellow) and formed networks in a similar manner (DIV 21). Stainings with GFAP (blue) indicated a successful integration of astrocytes into the culture. Related to **Figure 3A**.

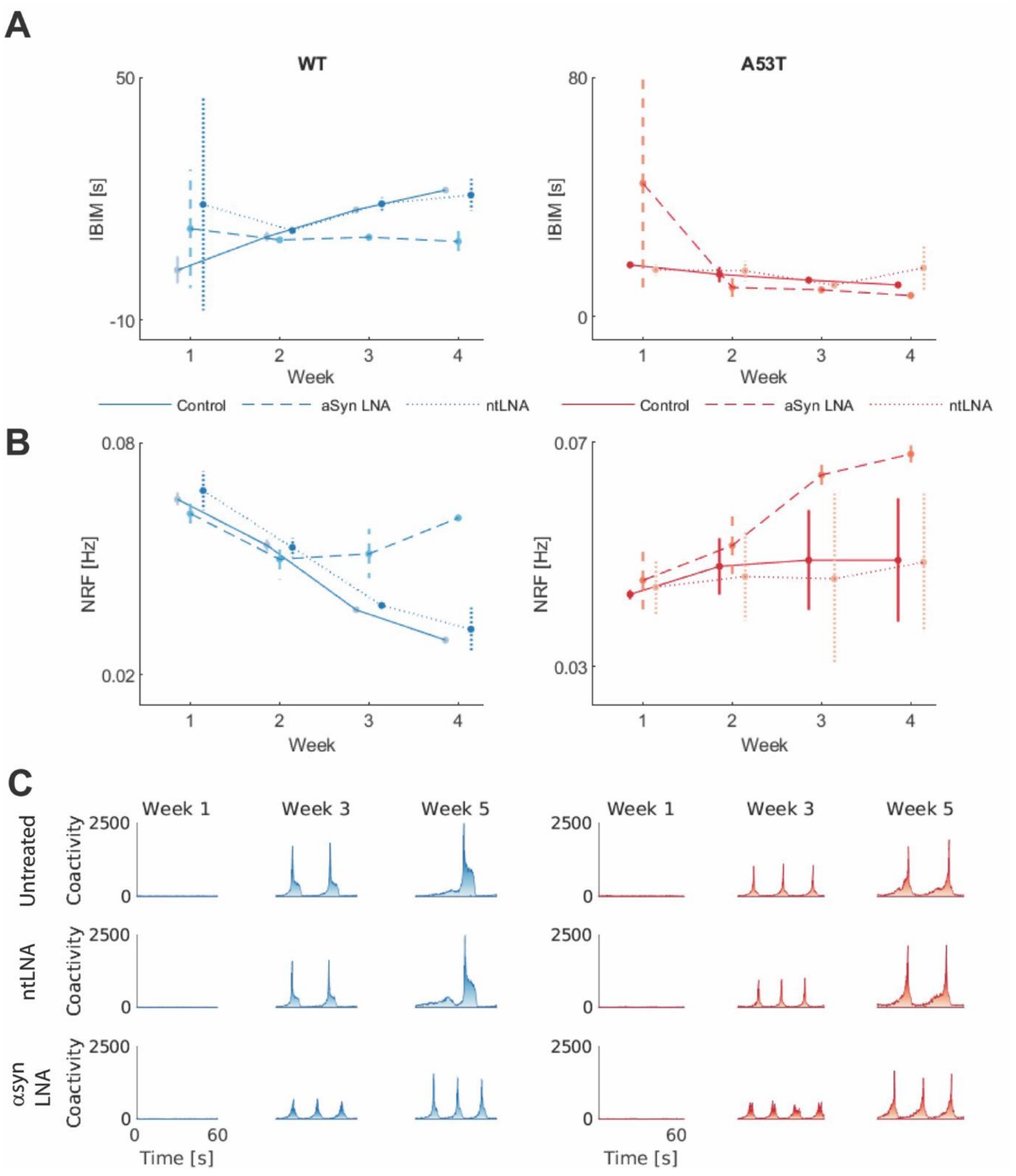

Figure S2. Similar developmental trajectories of untreated and non-targeted LNA-treated cultures. We found similar developmental trajectories in cultures treated with non-targeted LNA (ntLNA; dotted lines) and untreated control cultures (solid lines). Panels **(A)** and **(B)** underline this similarity and show two of the most distinctive features for the two genotypes - the mean interburst interval (IBIM; in panel **A**) and the network regularity frequency (NRF, in panel **B**). The anti-α-synuclein LNA (asyn LNA; dashed lines) treatment, however, had a clear effect on spontaneous activity (e.g., longer interburst intervals). The network coactivity plots in panel **(C)** further underscore this observation: While burst shapes and time intervals were very similar in untreated and ntLNA-treated cultures, αsyn LNA-treated cultures displayed shorter bursts with, on average, smaller amplitudes. Related to **Figure 4**.

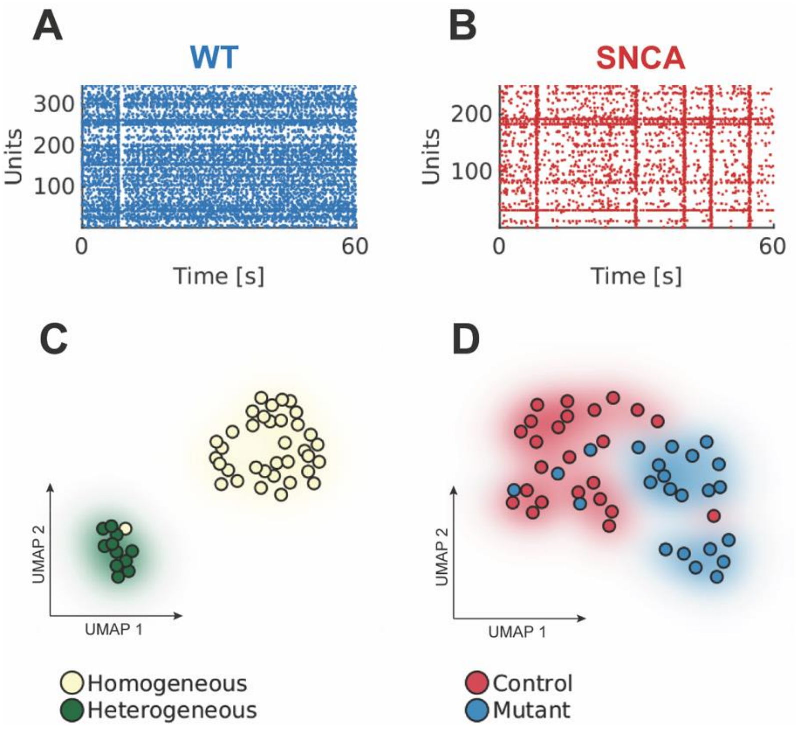

Figure S3. Robust differences between cell lines and cultures with different cellular compositions.

Representative spike raster plots of a heterogeneous midbrain DA WT/control culture **(A)** and a heterogeneous SNCA/mutant culture **(B)**. **(C)** Clustering analysis allowed for a clear separation into homogeneous and heterogeneous midbrain DA cultures (N=36 heterogeneous cultures and N=11 homogeneous cultures). **(D)** Control and mutant lines, here pooled across heterogeneous and homogenous cultures, can also be successfully separated in different clusters (N=24 control and N=23 mutant networks/cultures; the input data was normalized for homogeneous and heterogeneous cultures separately for this analysis). Related to **Figure 5**.

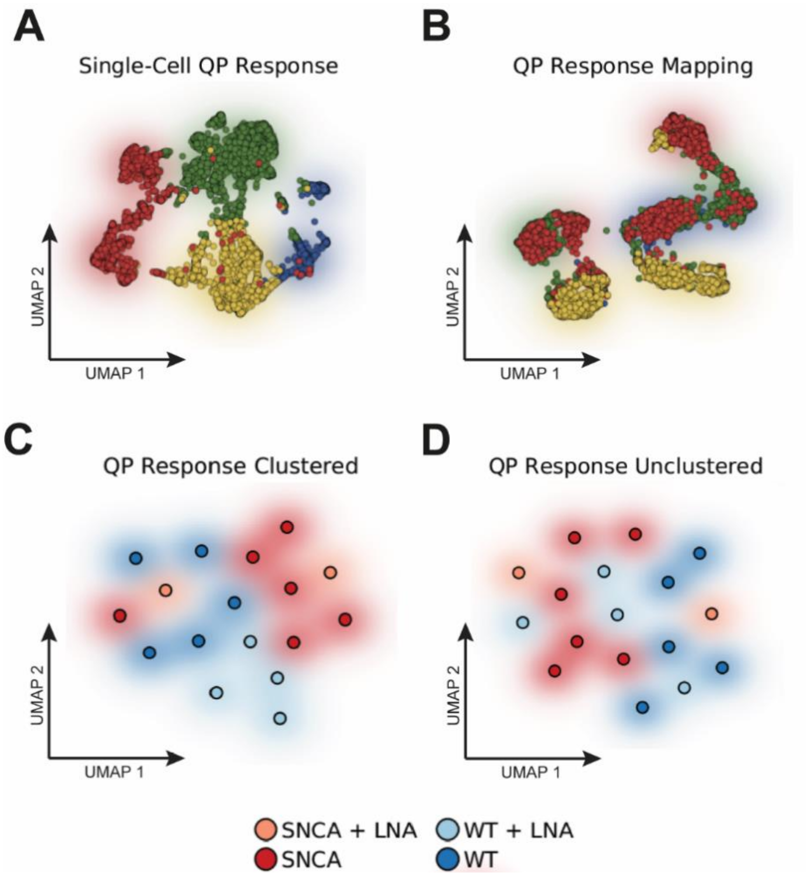

Figure S4. Integration of Quinpirole response modes facilitates phenotyping of heterogeneous cell lines.

(A) Results of a UMAP embedding performed on the single-cell responses after application of the selective D_2 and D_3 receptor agonist Quinpirole (QP). Each dot indicates one cell/response obtained from heterogeneous DA midbrain cultures (in total N=4932 units). Following the UMAP reduction, Louvain clustering was performed: each color (red, green, blue, and yellow) indicates a separate response cluster. **(B)** UMAP embedding based on the baseline activity; colors according to the clusters inferred in **(A)**. **(C)** UMAP embedding of the QP response after inferring single-cell features for each of the single-cell clusters from **(A)** separately (N=17 cultures). **(D)** UMAP embedding of the same QP response without using single-cell clustering, i.e., single-cell features were averaged across the whole network. Related to **Figure 5E-F**.

Supplemental tables

Table S1. Full feature list inferred by *DeePhys*.

Feature name	Feature description
**Single-cell features**	**Inferred from individual spike-sorted units**
**Waveform features**	**Features inferred from the action potential waveform shape**
AUCP1	Calculates the area under the curve of the action potential waveform peak 1
AUCP2	Calculates the area under the curve of the action potential waveform peak 2
AUCT	Infers the area under the curve of the action potential waveform trough
RISE	Calculates the slope from the action potential waveform trough to peak 2
DECAY	Calculates the slope from the action potential waveform peak 2 back to baseline
ASYM	Calculates the waveform asymmetry, i.e., the ratio of peak 2 and peak 1
T2PR	Calculates the trough-to-peak 2 ratio of the action potential waveform
T2PD	Calculates the trough-to-peak 2 delay of the action potential waveform
**Spike-time features**	**Features inferred from the spike-times of individual units**
MIS	Calculates the mean interspike interval of a spike train
VIS	Calculates the variance of the interspike interval of a spike train
CVI	Calculates the coefficient of variation of the interspike interval
PAF	Infers the partial autocorrelation function of a spike train
**Network features**	**Features inferred across the neuronal network**
**Burst features**	**Inferred from the network burst activity**
MIB	Infers the mean interburst interval, i.e., the interval times between bursts
VIB	Estimates the variance of the interburst intervals
MBD	Calculates the mean burst duration, i.e., the time from the start to the end of bursts
VBD	Measures the variance of the burst durations
MRT	Calculates the mean burst rise time, i.e., the time from 10% to 90% of the maximum coactivity (peak) of a burst
MFT	Calculates the mean burst fall time, i.e., the time from 90% to 10% of the maximum coactivity (peak) of a burst
MRV	Measures the mean burst rise velocity, i.e., the slope from 10% to 90% of the maximum coactivity (peak) of a burst
MFV	Calculates the mean fall velocity, i.e., the slope from 90% to 10% of the maximum coactivity (peak) of a burst
INTRABF	Gives the intraburst firing rate
INTERBF	Infers the interburst firing rate
**Graph features**	**Features inferred from functional connectivity**
DENS	Estimates the graph density, i.e., a measure of how many edges are present compared to the total number of possible edges
RE	Calculates the Rent exponent, i.e., a parameter indicating the scaling relationship between the size of a network and its average degree
ASRT	Estimates the assortativity, i.e., the degree to which nodes tend to be connected to nodes with similar degrees
GE	Calculates the global efficiency; the average inverse shortest path length in a network, it measures how efficiently information can flow in a graph

**Time-series features**	**Inferred from the binned activity of individual units or the whole network**
RF	Calculates the regularity frequency, i.e., the peak frequency of the Fourier power spectrum of a signal
RM	Calculates the regularity magnitude as the magnitude of the RF
RFIT	Estimates the regularity fit, i.e., the exponential fit of consecutive peaks in the Fourier power spectrum
EAF	Calculates the first 1 / e (Euler's number) crossing of the autocorrelation function
AMI	Estimates the auto-mutual information, i.e., the mutual information between a signal and its time-delayed version
SFR	Calculates the proportion of slower timescale fluctuations that scale with linearly rescaled range fits
SFD	Calculates the proportion of slower timescale fluctuations that scale with the Detrended Fluctuation Analysis
LPF	Estimates the total power in the lowest fifth of the frequencies in the Fourier power spectrum
MEF	Infers the mean error from a rolling 3-sample mean forecasting
PAM	Measures the longest period of consecutive values above the mean
EFD	Returns the exponential fit on the sequence of successive Euclidean distances between points in a 2D time-delay embedding space
CCD	Measures the change in correlation length, i.e., the distance over which the time series is correlated with itself, after iterative differencing
TCT	Calculates the trace of covariance of the transition matrix, i.e., the transition probabilities between different states in the time series
SES	Infers the Shannon entropy of two successive local motifs
CFS	Calculates the centroid of the Fourier power spectrum
PMW	Calculates the periodicity measure of [1]
MD5	Calculates the mode of *z*-scored distribution (5-bin histogram)
MD10	Calculates the mode of *z*-scored distribution (10-bin histogram)
FMA	Infers the first minimum of the autocorrelation function
FMI	Gives the first minimum of the automutual information function
TRS	Measures the time-reversal asymmetry, i.e., it quantifies whether the time series behaves differently when its direction is reversed in time
TEA	Gives the average of time intervals between successive extreme events above the mean
TEB	Gives the average of time intervals between successive extreme events below the mean
PDE	Calculates the proportion of successive differences exceeding 0.04 SD [2]
LSD	Infers the longest period of successive incremental decreases

Descriptions of the catch22 time series features (indicated in italics) were adapted from the original papers, which also contain a more detailed explanation [3, 4].

Table S2. List of main functions of *DeePhys*.

Module/Function	Description
**Preprocessing**	
generate_sorting_path_list	Generates a list of paths containing the sorting results (only works if all spike sortings follow the same path logic)
remove_low_unit_recording	Removes the MEArecording objects with too few units after QC
split_sortings	Splits sorting by time and generates new sorting files (useful for analyzing concatenated recordings)
recording_array_from_single_files	Loads individual MEArecording objects into one array
**Feature extraction and Quality control**	
generate_MEArecordings_from_sorting_list	Generates MEArecording objects from a spike sorting list
Class Unit	Instantiates an object that contains information about a single unit and a reference to its corresponding MEArecording
.inferActivityFeatures	Calculates activity features of individual units
Class MEArecording	Instantiates an object that contains all metadata and network feature values
.returnDefaultParams	Returns a structure containing the default parameter values
.performAnalyses	Runs quality control and feature extraction based on the *params* structure provided
.aggregateSingleCellFeatures	Returns a table containing the specified unit features (averages) across the culture
.calculateClusterSingleCellFeatures	Calculates the unit features for each single-cell cluster
.concatenateClusteredFeatures	Returns a table containing the unit features averages across single-cell clusters
.getRecordingFeatures	Returns a table containing the features representative of the whole culture (unit and network features)
.getUnitFeatures	Returns a table containing the unit features for each individual unit
.PlotNetworkScatter	Plots a scatter plot of the network activity
.PlotNetworkScatterHistogram	Same as PlotNetworkScatter, but with a corresponding histogram
.PlotBurstCheck	Plots network coactivity and burst starts and ends as inferred by the burst detection algorithm
.PlotCCG	Plots the crosscorrelogram between two units
.PlotCommunity	Plots connectivity matrix of an inferred graph after maximizing the modularity
.PlotUnitClusterActivity	Plots scatter plots sorted by unit cluster IDs
**Feature integration and phenotyping**	
Class RecordingGroup	Instantiates an object that contains analyses of a group of MEArecordings
.runMLM	Runs linear mixed-effects models to assess differences between conditions statistically
.aggregateSparseFeatureTable	Returns a table containing the features of cultures across development (allows missing values)
.aggregateCultureFeatureTables	Returns a table containing the features of cultures across development
.prepareInputMatrix	Returns input table for machine learning methods

.reduceDimensionality	Performs the selected dimensionality reduction method on the single-cell or network level
.predictAge	Performs random forest regression to predict the age of a culture
.classifyByFeatureGroups	Performs classification between conditions based on the selected feature groups
.assessClassifier	Assesses the performance of the classifier
.classifyByFeatureGroupsAndGroupingVar	Trains classifiers for each feature group and each condition individually
.regressionByFeatureGroups	Performs random forest regression to predict any numerical metadata information (e.g., drug concentration)
.combineMetadataIndices	Returns group indices corresponding to the selected metadata information
.returnFeatureNames	Returns names of features that have been extracted
.clusterByFeatures	Cluster cultures or units using the selected clustering algorithm
.calculateClusterPurity	Calculates the cluster purity of the selected clustering result (requires ground truth)
.plot_feature_trajectories	Plots a line plot of feature values of distinct conditions (e.g., cell lines) across development
.plot_feature_heatmap	Plots a heatmap color coding the relative feature values between two conditions
.plot_regression_results	Plots box plot of regression results
**Assessment of (pharmacological) interventions**	
.applyClassifier	Applies a pretrained classifier to a new group of recordings
.assessAppliedClassifier	Assesses the performance of the pretrained classifier on the new test data
**Single-cell analyses**	
.assignUnitClusterIdx	Permanently assigns a cluster ID to a unit (required for subsequent single-cell analyses)
.removeUnitsByCluster	Removes units belonging to a selected cluster
.classifyClusteredUnits	Trains a classifier based on the results of a single-cell clustering
.applyClusteredUnitClassifier	Applies pretrained classifier on other cultures
.plot_true_clusters	Plots single-cell dimensionality reduction colored by the selected metadata information (e.g., cell line)
.plot_cluster_outlines	Plots dimensionality reduction scatter plot and displays cluster outlines
.plot_single_cluster	Plots dimensionality reduction of a single selected cluster
.plot_cluster_waveforms	Plots waveforms of units belonging to the inferred clusters
.plot_cluster_densities	Plots a heatmap of single-cell cluster densities after dimensionality reduction
.plot_cluster_shifts	Plots differences between cluster densities of different conditions (e.g., cell lines)
.plot_cluster_proportions	Plots a cumulative bar plot of the cluster proportions in distinct conditions (e.g., cell lines)
.plot_unit_cluster_features	Plots a line plot of mean feature values of single-cell clusters
.plot_unit_cluster_heatmap	Plots a heatmap coding the relative feature values of clusters

Table S3. Ratio of TH+/MAP2+ neurons.

Genotype	Treatment	TH+/MAP2+ ratio [%]												Mean ± SD
WT	Untreated	36.9	64.1	57.0	33.6	56.6	62.7	36.5	53.4	62.0	40.6	59.0	70.5	49.16 ± 13.94
		34.8	41.3	58.9	21.3	30.7	41.2	30.1	65.9	60.8	33.8	70.1	57.9	
		41.5	70.8	57.8	42.4	64.9	65.9	41.1	51.9	40.7	32.8	45.7	33.7	
	ntLNA	47.8	51.2	35.2	61.2	55.0	42.8	53.9	57.4	36.0	66.7	61.1	49.3	49.42 ± 9.62
		57.8	42.2	58.8	50.0	47.7	43.2	39.5	56.6	59.7	30.3	58.5	63.0	
		32.8	56.8	52.7	37.6	58.3	43.3	42.0	52.1	45.1	33.6	45.1	55.0	
	LNA	27.2	59.0	54.1	38.7	62.9	58.5	32.9	50.8	58.0	30.1	56.5	54.9	43.81 ± 13.29
		34.6	49.8	56.7	23.4	34.4	40.6	22.6	41.6	34.1	30.6	68.0	58.8	
		51.4	46.8	43.3	40.0	51.1	51.1	30.7	54.0	56.1	18.5	30.9	25.1	
A53T	Untreated	39.3	27.6	28.0	54.0	27.6	28.0	59.4	40.7	38.2	47.4	50.2	39.8	43.93 ± 11.33
		51.9	44.4	52.3	58.0	69.4	52.9	38.9	30.7	33.8	50.2	37.3	27.3	
		51.4	41.8	26.4	53.3	61.8	38.2	56.6	40.8	38.5	59.1	45.6	40.6	
	ntLNA	59.9	42.6	23.3	50.0	44.2	25.0	45.8	42.9	38.6	48.7	58.2	39.0	42.10 ± 13.91
		54.8	56.2	38.6	41.6	47.2	42.3	33.5	28.3	4.44	35.1	39.3	23.1	
		50.5	34.2	23.1	62.2	58.8	24.2	53.5	57.3	27.5	64.6	59.5	37.4	
	LNA	30.4	16.1	21.6	37.9	19.2	35.4	29.8	27.6	31.7	40.5	29.6	37.0	31.65 ± 8.99
		32.6	25.6	17.6	15.3	24.3	22.0	31.3	28.2	21.7	40.5	33.3	34.7	
		39.1	46.6	30.9	54.1	38.7	31.4	31.3	49.8	36.2	38.5	34.8	24.1	

ANOVA table	SS	DF	MS	F (DFn, DFd)	p value
Interaction	0.046	2	0.023	F (2, 210) = 1.6	p=0.208
Mutation	0.366	1	0.366	F (1, 210) = 25.4	p<0.001
Treatment	0.343	2	0.171	F (2, 210) = 11.9	p<0.001
Residual	3.031	210	0.014		

Tukey-Kramer test	Adjusted p value	Tukey-Kramer test	Adjusted p value
WT:Untreated vs. WT:ntLNA	>0.999	WT:ntLNA vs. A53T:LNA	<0.001
WT:Untreated vs. WT:LNA	0.607	WT:LNA7 vs. A53T:Untreated	>0.999
WT:Untreated vs. A53T:Untreated	0.643	WT:LNA vs. A53T:ntLNA	0.999
WT:Untreated vs. A53T:ntLNA	0.184	WT:LNA vs. A53T:LNA	<0.001
WT:Untreated vs. A53T:LNA	<0.001	A53T:Untreated vs. A53T:ntLNA	0.999
WT:ntLNA vs. WT:LNA	0.530	A53T:Untreated vs. A53T:LNA	<0.001
WT:ntLNA vs. A53T:Untreated	0.566	A53T:ntLNA vs. A53T:LNA	0.004
WT:ntLNA vs. A53T:ntLNA	0.146		

Ratio of TH$^+$/MAP2$^+$ cells per imaged field. For each condition, six cultures were analyzed by quantifying the intensity of six fields, each consisting of 3x3 images. Related to **Figure 2A**.

Table S4. Quantification of total α-synuclein levels by Homogeneous Time Resolved Fluorescence (HTRF) assay.

Genotype	Treatment	HTRF [intensity]									Mean ± SD
WT	Untreated	63.8	64.6	65.7	70.2	70.1	70.4	64.2	65.3	65.5	66.6 ± 2.8
	ntLNA	74.3	74.9	73.4	70.7	71.1	70.3	71.1	71.2	71.2	72.0 ± 1.7
	LNA	5.4	5.6	5.5	5.5	5.6	5.5	5.2	5.3	5.1	5.4 ± 0.2
A53T	Untreated	74.3	72.9	72.6	63.9	66	60.8	63.7	63.8	64.5	66.9 ± 4.9
	ntLNA	71.3	71.9	72.5	67.9	67.2	68.7	39.8*	39.5*	40.0*	69.9 ± 2.3
	LNA	5.5	5.9	5.8	6.5	6.3	6.4	6.5	6.7	6.4	6.2 ± 0.4

ANOVA table	SS	DF	MS	F (DFn, DFd)	p value
Interaction	6.2	2	3.1	F (2, 11) = 0.4	p=0.705
Mutation	0.5	1	0.5	F (1, 11) = 0.05	p=0.820
Treatment	15282.7	2	7641.3	F (2, 11) = 885.3	p<0.001
Residual	94.9	11	8.6		

Tukey-Kramer test	Adjusted p value	Tukey-Kramer test	Adjusted p value
WT:Untreated vs. WT:ntLNA	0.293	WT:ntLNA vs. A53T:LNA	<0.001
WT:Untreated vs. WT:LNA	<0.001	WT:LNA7 vs. A53T:Untreated	<0.001
WT:Untreated vs. A53T:Untreated	>0.999	WT:LNA vs. A53T:ntLNA	<0.001
WT:Untreated vs. A53T:ntLNA	0.819	WT:LNA vs. A53T:LNA	0.999
WT:Untreated vs. A53T:LNA	<0.001	A53T:Untreated vs. A53T:ntLNA	0.868
WT:ntLNA vs. WT:LNA	<0.001	A53T:Untreated vs. A53T:LNA	<0.001
WT:ntLNA vs. A53T:Untreated	0.345	A53T:ntLNA vs. A53T:LNA	<0.001
WT:ntLNA vs. A53T:ntLNA	0.964		

Quantification of total α-synuclein levels by a Homogeneous Time Resolved Fluorescence (HTRF) assay. For each condition, three biological replicates (different cultures), each with three technical replicates were analyzed. The samples indicated with asterisks were considered outliers and excluded from the analysis. Related to **Figure 4B**.

Table S5. Quantification of somatic α-synuclein levels by immunocytochemical staining analysis.

Genotype	Treatment	Somatic α-synuclein levels [intensity]									Mean ± SD
WT	Untreated	817.6	674.8	683.3	744.5	719.4	701.7	665	698.9	667.7	721.4 ± 57.6
		802	726.2	754	660.3	621.8	691.5	808.7	753.2	794	
	ntLNA	791.9	712.4	824.3	584.2	648.6	615.6	635.1	734.2	683.4	723.1 ± 91.3
		731.6	713.4	702.7	600.6	637.7	559.5	821	830.7	792.4	
	LNA	271	258.1	273.2	256	296.3	272.9	264.2	276	256.6	272.4 ± 13.9
		284.6	299.8	265.9	270.5	273.2	249.6	269.6	272	293	
A53T	Untreated	781.4	786.2	720	690.3	806.5	859.1	689.8	733.6	690.9	721.5 ± 75.6
		791.9	712.4	824.3	584.2	648.6	615.6	635.1	734.2	683.4	
	ntLNA	878.2	869.9	892.5	872.8	801.6	793.7	734.9	767.6	684	775.1 ± 66.9
		733.4	764.7	738.6	701.3	675.2	721.6	793.7	756.1	771.1	
	LNA	291.5	252.7	257.6	273.9	298.6	283.8	269.3	288.6	287.4	275.9 ± 12.6
		274.4	281.3	282	271.3	258.3	274.9	267.9	265.9	286.1	

ANOVA table	SS	DF	MS	F (DFn, DFd)	p value
Interaction	15148.8	2	7574.3	F (2, 102) = 2.0	p=0.134
Mutation	9292.6	1	9292.6	F (1, 102) = 2.5	p=0.116
Treatment	5117295.0	2	2558647.5	F (2, 102) = 691.9	p<0.001
Residual	377216.7	102	3698.2		

Tukey-Kramer test	Adjusted p value	Tukey-Kramer test	Adjusted p value
WT:Untreated vs. WT:ntLNA	>0.999	WT:ntLNA vs. A53T:LNA	<0.001
WT:Untreated vs. WT:LNA	<0.001	WT:LNA7 vs. A53T:Untreated	<0.001
WT:Untreated vs. A53T:Untreated	>0.999	WT:LNA vs. A53T:ntLNA	<0.001
WT:Untreated vs. A53T:ntLNA	0.132	WT:LNA vs. A53T:LNA	>0.999
WT:Untreated vs. A53T:LNA	<0.001	A53T:Untreated vs. A53T:ntLNA	0.134
WT:ntLNA vs. WT:LNA	<0.001	A53T:Untreated vs. A53T:LNA	<0.001
WT:ntLNA vs. A53T:Untreated	>0.999	A53T:ntLNA vs. A53T:LNA	<0.001
WT:ntLNA vs. A53T:ntLNA	0.163		

Quantification of somatic α-synuclein levels by immunocytochemical stainings. For each condition, three cultures were analyzed by quantifying the intensity of six fields, each consisting of 3x3 images. Related to **Figure 4**.

Table S6. Quantification of somatic phosphorylated α-synuclein levels by immunocytochemical stainings.

Genotype	Treatment	Somatic phosphorylated α-synuclein levels [intensity]									Mean ± SD
WT	Untreated	281.3	197.1	210.3	258.5	231.5	216.1	197	207.9	201.7	225 ± 22.9
		218.6	227.7	217.6	225.7	215.6	208.2	244.9	254.4	238.1	
	ntLNA	215.9	234.7	239.9	211.5	233.8	284	213	213.3	246.3	226.9 ±21.4
		211.5	203.5	248.1	201.5	196	230.3	238.9	238.9	222.4	
	LNA	156.1	157.1	155.8	161.4	160.6	160.9	160.3	157.8	157.1	159.6 ± 2.4
		163.7	161.1	160.7	160.6	157.2	157.7	161.2	164	159.6	
A53T	Untreated	239.6	247.8	265.4	233.2	245.9	290	207.7	207.5	271.3	235.1 ± 24.7
		224.5	211.1	256.9	210.3	201.9	244	227.7	219.7	226.6	
	ntLNA	234.4	238.9	260.6	249.5	226.2	293.2	249	216	275.6	246.8 ± 20.4
		238	232.4	245.3	227.2	227.5	236.3	253.5	275.5	264.1	
	LNA	159.1	175	160.4	163.2	171.1	166.2	163.9	167.3	163.8	165.2 ± 3.7
		162.9	165.7	166.9	162.6	164.5	165.4	167.1	162.6	166.5	

ANOVA table	SS	DF	MS	F (DFn, DFd)	p value
Interaction	976.7	2	488.3	F (2, 102) = 1.4	p=0.241
Mutation	3791.4	1	3791.4	F (1, 102) = 11.2	p=0.001
Treatment	121986.8	2	60993.4	F (2, 102) = 180.1	p<0.001
Residual	34547.8	102	338.7		

Tukey-Kramer test	Adjusted p value	Tukey-Kramer test	Adjusted p value
WT:Untreated vs. WT:ntLNA	0.999	WT:ntLNA vs. A53T:LNA	<0.001
WT:Untreated vs. WT:LNA	<0.001	WT:LNA7 vs. A53T:Untreated	<0.001
WT:Untreated vs. A53T:Untreated	0.587	WT:LNA vs. A53T:ntLNA	<0.001
WT:Untreated vs. A53T:ntLNA	0.008	WT:LNA vs. A53T:LNA	0.941
WT:Untreated vs. A53T:LNA	<0.001	A53T:Untreated vs. A53T:ntLNA	0.396
WT:ntLNA vs. WT:LNA	<0.001	A53T:Untreated vs. A53T:LNA	<0.001
WT:ntLNA vs. A53T:Untreated	0.764	A53T:ntLNA vs. A53T:LNA	<0.001
WT:ntLNA vs. A53T:ntLNA	0.018		

Quantification of somatic phosphorylated α-synuclein levels by immunocytochemical stainings. For each condition, three cultures were analyzed by quantifying the intensity of six fields, each consisting of 3x3 images. Related to **Figure 4**.

Table S7. Spike-sorting parameters for Kilosort 2.5.

detect_threshold	5.5	projection_threshold	[10 4]
preclust_threshold	8	car	True
minFR	0.01	minfr_goodchannels	0.01
nblocks	5	sig	20
freq_min	150	sigmaMask	30
nPCs	3	ntbuff	64
nfilt_factor	4	NT	None
do_correction	False	wave_length	61
keep_good_only	False	skip_kilosort_preprocessing	False
scaleproc	None	save_rez_to_mat	False
delete_tmp_files	True	delete_recording_dat	False
n_jobs	-1	chunk_duration	1s

Table S8. Random Forest hyperparameter optimization parameters.

Hyperparameter	Search range
MinLeafSize	range(1,max(2, floor(NumObservations/2)))
MaxNumSplits	range(1, max(2,NumObservations-1))
NumLearningCycles	range(10, 500)
SplitCriterion	gdi, deviance, twoing
NumVariablesToSample	range(1, max(2,NumPredictors))

Further information about the hyperparameters can be found in the official Matlab documentation.

Table S9. List of primary and secondary antibodies.

**Primary antibody**	**Dilution**	**Catalogue number**
mouse anti-TH	1:500	#MAB318, Sigma-Aldrich
chicken anti-MAP2	1:1000	#CH22103, Neuromics (Edina, MN, USA)
rabbit anti-GFAP	1:500	#Z0334, Agilent (Santa Clara, CA, USA)
rabbit anti-phospho-α-synuclein	1:100	Britschgi lab, Roche (Basel, Switzerland)
rabbit anti-α-synuclein	1:100	#2628, Cell Signaling Technology (Danvers, MS, USA)
**Secondary antibody**		
donkey anti-mouse 488	1:250	#A-21202, ThermoFisher
goat anti-chicken 647	1:500	#A-32933, ThermoFisher
donkey anti-rabbit 568	1:250	#A-A10042, ThermoFisher

Supplemental experimental procedures

High-density microelectrode array recordings

Electrophysiological recordings were obtained using two types of complementary-metal-oxide-semiconductor (CMOS)-based high-density microelectrode arrays (HD-MEA) by MaxWell Biosystems (MaxWell Biosystems, Zurich, Switzerland): the single-well HD-MEA "MaxOne" and the 6-well plate HD-MEA "MaxTwo". The wells of both types of HD-MEAs feature a total of 26'400 electrodes in a 120 x 220 electrode grid with a microelectrode center-to-center spacing of 17.5 µm, an overall sensing area of 3.85 × 2.10 mm^2, and allows for simultaneous recordings from up to 1024 electrodes at a sampling rate of 20 kHz (MaxOne) or 10 kHz (MaxTwo)[5]. Recordings were performed inside an incubator at 36°C and 5% CO_2 using the MaxLab Live recording software (MaxWell Biosystems). Recordings started at day *in vitro* (DIV) 7 and were subsequently performed once a week over the course of 5 weeks. Each recording consisted of an activity scan to determine the electrode selection and a subsequent network recording. The activity scan consisted of 7 sparse electrode configurations (center-to-center spacing: 35 µm, every 2nd electrode), that were recorded 2 minutes each. To capture the dynamics of developing neuronal networks, electrodes displaying the highest firing rate were selected; spontaneous network activity was recorded for 15 minutes.

Cell culture and plating

Cell lines

Homogeneous neuronal cultures: Human iPSC-derived DA neurons carrying a heterozygous A53T mutation (cat. C1112, iCell DopaNeurons A53T), an isogenic control line (cat. C1087, iCell DopaNeurons) and astrocytes (cat. R1092, iCell Astrocytes) were purchased from FUJIFILM Cellular Dynamics International (FCDI, Madison, WI, United States). The A53T cell line was generated through nuclease-mediated single-nucleotide polymorphism alterations of the isogenic control line. Midbrain DA neuron differentiation from iPSCs was based on a protocol from the Lorenz Studer lab[6]. The vendor guarantees a purity of at least 70% for midbrain DA neurons and 95% for astrocytes.

The protocol to plate human neurons on HD-MEAs was previously established by our group[7]. Prior to cell plating, HD-MEAs were sterilized in 70% ethanol for 30 minutes and rinsed 3 times with sterile deionized (DI) water. To enhance cell adhesion, the electrode area was covered with 10 µL of 0.05 mg/mL poly-L-ornithine (PLO) solution (cat. A-004-C, Sigma-Aldrich, Saint Louis, MO, United States) and incubated at 37°C for 2 hours. Next, the PLO solution was aspirated, and the HD-MEA was rinsed 3 times with sterile DI water. Next, we added 10 µL of 80 µg/ml laminin (cat. L2020-1MG, Sigma-Aldrich) in plating medium (see below) directly on the electrode area and incubated the chips at 37°C for 30 minutes. The plating medium consisted of 95 mL of BrainPhys Neuronal Medium (cat. 05790, STEMCELL Technologies, Vancouver, Canada), 2 mL of iCell Neural Supplement B (cat. M1029, FCDI), 1 mL iCell Nervous System Supplement (cat. M1031, FCDI), 1 mL N-2 Supplement (100X, cat. 17502048, Gibco), 100 µL laminin (1 mg/mL, cat. L2020-1MG, Sigma-Aldrich) and 1 mL Penicillin-Streptomycin (100X, cat. 15140122, Gibco). In the meantime, the cryovials containing the DA neurons and astrocytes were thawed in a 37°C water bath for 3 minutes. The cells were then transferred to 50 mL centrifuge tubes, and 8 mL plating medium (at room temperature) were drop-wise added (numbers are indicated for 20 chips). Next, the cell suspensions were centrifuged at 380 g for 5 minutes, and the supernatant was aspirated. Cell pellets were then resuspended in plating medium and combined to achieve a final concentration of 10'000 DA neurons and 2000 astrocytes per µL. Finally, 100'000 DA neurons and 20'000 astrocytes were seeded on each HD-MEA by adding 10 µL of the prepared solution directly to the laminin droplet. Next, chips were incubated for 1 hour, and 1.5 mL of plating medium were carefully added. Chips were equipped with a lid, placed inside a 100 mm petri dish - to facilitate transport and to reduce the risk of contamination - and kept inside an incubator at 37°C and 5% CO_2. Additionally, a 35-mm petri dish, filled with DI water, was placed inside the larger petri dish to counteract evaporation. One day after the plating, we replaced 50% of the medium and resumed the normal media change protocol (one third of the medium was exchanged twice a week). Cultures were allowed to equilibrate for 3 days after the medium change to prevent effects on the recordings.

Heterogeneous neuronal cultures: We used the following human iPSC lines: The mutant line SNCA^{tri} (SFC831-03-01, https://cells.ebisc.org/STBCi024-A/, [8]) purchased from the European Bank for

induced pluripotent Stem Cells (EBiSC), and a control iPSC line (SFC840-03-01, https://hpscreg.eu/cell-line/STBCi026-B, [9]) purchased from EBiSC. To derive DA neurons from human iPSCs, an mFPP-based DA neuron differentiation protocol was used [6, 10]. Briefly, human iPSCs were disaggregated using Accutase (cat. AT104, Innovative Cell Technologies Inc., San Diego, CA, United States) for 5-10 minutes, contrifuged at 100 g for 4 minutes and plated on Matrigel-coated (cat. 354277, BD Biosciences, Franklin Lakes, New Jersey, United States) multiwells in the presence of Rock inhibitor (cat. 688000, Sigma-Aldrich) at the density of 200’000 cells/cm^2. DA neuron differentiation was induced when the cells reached a confluent state. In the floor plate induction phase, from day 0 to day 10, 100 nM StemMACS™ LDN193189 (cat.130-103-925, Miltenyi Biotec, Bergisch Gladbach, Germany) and from day 0 to day 4, 10 µM StemMACS™ SB431542 (cat. 130-106-543, Miltenyi Biotec) were added to the medium. From day 1 to day 6, the medium contains 100 ng/mL Human SHH (C24II) (cat. 130-095-727, Miltenyi Biotec), 100 ng/mL Human FGF-8b (cat. 130-095-740, Miltenyi Biotec) and 2µM StemMACS™ Purmorphamine (cat. 130-104-465, Miltenyi Biotec). 3 µM StemMACS™ CHIR99021 (cat. 130-103-926, Miltenyi Biotec) was added from day 3 until day 11. Floor plate induction medium (see below for details) was used from day 0 till day 10. On day 5 of differentiation, the floor plate induction medium was gradually shifted to the floor plate expansion medium (see below for details) to contain 25%, 50% and 75%.

Prior to cell plating, HD-MEAs were sterilized in 70% ethanol for 30 minutes and rinsed 3 times with sterile deionized (DI) water. To enhance cell adhesion, the electrode area was covered with 10 µL of 0.05 mg/mL poly-L-ornithine (PLO) solution (cat. A-004-C, Sigma-Aldrich) and incubated at 37°C overnight. Next, the PLO solution was aspirated, and the HD-MEA was rinsed 3 times with sterile DI water. We then added 10 µL of 20 µg/ml laminin (cat. 11 243 217 001, Sigma-Aldrich) directly on the electrode area and incubated the chips at 37°C for 1 hour. Human iPSCs were disaggregated using Accutase for 5-10 minutes, centrifuged at 100 g for 4 minutes and 120’000 neurons were seeded on the HD-MEA by adding 10 µL of the prepared cell solution. Next, chips were incubated for 1 hour, and 1.2 mL of plating medium were carefully added. From the day of the plating (day 11) onwards, DA neuron progenitors were induced to the DA neuron fate by shifting to DA differentiation medium supplemented with 20 ng/mL Human BDNF (cat. 130-096-286, Miltenyi Biotec), 20 ng/mL Human GDNF (cat. 130-098-449, Miltenyi Biotec), 1 ng/mL Human TGF-β3 (cat.130-094-007, Miltenyi Biotec), 200 µM ascorbic acid (cat. A4034, Sigma-Aldrich), 0.5 mM dibutyryl cAMP (cat. D0627, Sigma-Aldrich) and 10 µM DAPT (cat. 2634, Tocris Bioscience, Minneapolis, Minnesota, United States). Twice a week, 1 mL of DA differentiation medium was replaced.

The floor plate induction medium_contains KnockOut™ DMEM (cat. 10829-018, Gibco) supplemented with 15% KnockOut™ Serum Replacement (cat. 10828010, Gibco), 2 mM GlutaMAX™ Supplement (cat. 35050-038, Gibco), 1X MEM Non-Essential Amino Acids (cat. 11140-035, Gibco), 50 U/mL Penicillin-Streptomycin (PenStrep) (cat. 15140122, Gibco) and 55 µM 2-Mercaptoethanol (cat. 21985-023, Gibco).

The floor plate expansion medium_contains DMEM/F12 with HEPES buffer (cat. 11330-032, Gibco) supplemented with 1X N-2 Supplement (cat. 17502-048, Gibco), 2 mM GlutaMAX™ Supplement, 0.0016 g/mL D-Glucose and 50 U/mL PenStrep.

The DA neuron differentiation medium contains Neurobasal medium (cat. 21103-049, Gibco) supplemented with 1X B-27™ Supplement, serum free (cat. 17504-044, Gibco), 2 mM GlutaMAX™ Supplement, and 50 U/mL PenStrep.

HD-MEA datasets

Inclusion criteria: In the present study, only cultures with recordings from all recording time points were included in the classification analysis. HD-MEA recordings were excluded, if a culture showed signs of detachment, or if the HD-MEA displayed severe malfunctions.

Genotype comparison: The dataset for the genotype comparison (**Figure 3**) consisted of N=18 WT and N=19 A53T cultures, pooled across two batches with identical cell culture and recording protocols. No statistical method was used to predetermine the sample size. For the classification analysis, the dataset consisted of N=14 WT and N=15 A53T cultures. A fraction of the dataset used in this study has been generated for a previous study by our group[7], which, however, did not include a more systematic analysis of waveform/network features of spike-sorted units.

LNA treatment: The dataset of the LNA treatment (**Figure 4**) consisted of N=10 WT and N=10 A53T cultures for the statistical analysis, and N=8 WT and N=8 A53T cultures for the classification analysis.

Single-cell analysis: The dataset for the single-cell analysis (**Figure 5**) consisted of a total of 18'174 cells pooled from 31 cultures.

Clustering

Data was batch-wise transformed into z-scores to minimize inter-batch variability, and z-scores were then used to perform Uniform Manifold Approximation and Projection (UMAP) [11]. The clustering procedure differed between networks and single-cell inputs: To calculate the separability of network genotypes in the low dimensionality space, k-means clustering (k=2) was performed on the UMAP embedding. Cluster centroid positions were initialized 100 times using the k-means++ algorithm in Matlab, and the solution with the lowest within-cluster sums of point-to-centroid distances was reported (i.e., here the squared Euclidean distance). Finally, the clustering purity was computed as: $\frac{1}{N}\sum_{i=1}^{k}|c_i \cap t_j|$ where N is the number of data points, k is the number of clusters, c_i is a cluster, and t_j is the classification with the maximum count for cluster c_i. Single-cell clustering was performed as previously described [12] by applying the Louvain community detection algorithm [13] on the UMAP graph obtained from single-cell waveform or the combination of single-cell waveforms and activity features.

Age prediction

The prediction of culture age was performed by training random forest regression models (Matlab function *fitrensemble*) on features from individual time points, and results were obtained using leave-one-out cross-validation. The assessment of the LNA treatment on the development was performed using random forest regression models trained on all untreated cultures of the same genotype.

Feature inference

Single-cell features

Waveform features:

1. *Half width (HLFW)* was defined as the width of the trough at half the trough amplitude value V_U^{trough} of one unit *U*.
2. *Asymmetry (ASYM)* was defined as the ratio of the difference and the sum of the peaks after (V_U^{peak2}) and before (V_U^{peak1}) the trough of unit *U*:
$$ASYM_U = \frac{V_U^{peak2} - V_U^{peak1}}{V_U^{peak2} + V_U^{peak1}}$$
3. *Trough-to-peak ratio (T2PR)* was defined as the absolute value of the ratio of the trough V_U^{trough} and the second peak V_U^{peak2} of unit *U:*
$$T2PR = \left|\frac{V_U^{trough}}{V_U^{peak2}}\right|$$
4. *Trough-to-peak delay (T2PD)* was defined as the time difference between the occurrence of the trough t_U^{trough} and the second peak t_U^{peak2} of one unit *U*:
$$T2PD = t_U^{peak2} - t_U^{trough}$$
5. *Peak area under the curve (AUCP)* was defined as the integral of the waveform WF_u between the zero crossings before (z_U^1) and after (z_U^2) the respective peak V_U^{peak1} or V_U^{peak2} of one unit *U*:
$$AUCP = \int_{z_U^1}^{z_U^2} WF_u$$
6. *Trough area under the curve (AUCT)* was defined as the integral of the waveform WF_u between the zero crossings before (z_U^1) and after (z_U^2) the trough V_U^{trough} of one unit *U*:
$$AUCT = \int_{z_U^1}^{z_U^2} WF_u$$
7. *Rise (RISE)* was defined as the slew rate from V_U^{trough} to V_U^{peak2} (10% to 90%) of one unit *U*:
$$RISE = \frac{V_U^{peak2} - V_U^{trough}}{t_U^{peak2} - t_U^{trough}}$$
8. *Decay (DECAY)* was defined as the slew rate from V_U^{peak2} to the resting potential V_U^{rest} (10% to 90%) of one unit *U*:
$$DECAY = \frac{V_U^{rest} - V_U^{peak2}}{t_U^{rest} - t_U^{peak2}}$$

Spike-time features:

9. *Mean interspike interval (MIS)* was defined as the average time between spiking events $ISI_i = t_{i+1}^{sp} - t_i^{sp}$ of one unit *U* over a defined number of spikes *N*:
$$MIS_U = \frac{1}{N-1}\sum_{i=1}^{N-1} ISI_i$$
10. *Interspike interval variance (VIS)* was defined as the variance of interspike intervals ISI_i of one unit *U* over a defined number *N* of ISIs:
$$VIS_U = \frac{1}{N}\sum_{i=1}^{N}(ISI_i - ISIM)^2$$
11. *Interspike interval coefficient of variation (CVI)* was defined as the ratio of the standard deviation to the mean of the interspike intervals ISI_i of one unit *U*:
$$CVI_U = \frac{\sqrt{ISIV_U}}{ISIM_U}$$
12. *Partial autocorrelation function (PAF)* was defined as the partial autocorrelation of lag 1 for all ISI_t of one unit *U*:

$$PAF = corr(ISI_{t+1}, ISI_t)$$

Network features

Burst features

1. *Mean interburst interval (MIB)* was defined as the average time from the end of one burst to the beginning of the next burst $IBI_i = t_{i+1}^{start} - t_i^{end}$ across all *N* bursts of one recording:
$$MIB = \frac{1}{N-1}\sum_{i=1}^{N-1} IBI_i$$
2. *Interburst interval variance (VIB)* was defined as the variance across all *N* IBIs of one recording:
$$VIB = \frac{1}{N}\sum_{i=1}^{N}(IBI_i - IBIM)^2$$
3. *Mean burst duration (MBD)* was defined as the average time from beginning t_i^{start} to the end t_i^{end} of a burst across all *N* bursts of one recording:
$$MBD = \frac{1}{N}\sum_{i=1}^{N} t_i^{end} - t_i^{start}$$
4. *Burst duration variance (VBD) was* defined as the variance across all *N* BDs of one recording:
$$VBD = \frac{1}{N}\sum_{i=1}^{N}(BD_i - MBD)^2$$
5. *Intra-burst firing rate (INTRABF)* was defined as the number of spikes n_B^{sp} during the total bursting time T_B of one recording:
$$INTRABF = \frac{n_B^{sp}}{T_B}$$
6. *Inter-burst firing rate (INTERBF)* was defined as the number of spikes n_{NB}^{sp} during the total non-bursting time T_{NB} of one recording:
$$INTERBF = \frac{n_{NB}^{sp}}{T_{NB}}$$
7. *Burst rise time (BRT)* was defined as the average time from the beginning t_i^{start} to the peak t_i^{peak} of a burst across all *N* bursts of a recording:
$$BRT = \frac{1}{N}\sum_{i=1}^{N} t_i^{peak} - t_i^{start}$$
8. *Burst rise velocity (BRV)* was defined as the average slew rate from the coactivity at the beginning C_i^{start} to the coactivity at the peak C_i^{peak} of a burst (10% to 90%) across all *N* bursts of a recording:
$$BRV = \frac{1}{N}\sum_{i=1}^{N}\frac{C_i^{peak} - C_i^{start}}{t_i^{peak} - t_i^{start}}$$
9. *Burst fall time (BFT)* was defined as the average time from the peak t_i^{peak} to the end t_i^{end} of a burst across all *N* bursts of a recording:
$$BFT = \frac{1}{N}\sum_{i=1}^{N} t_i^{end} - t_i^{peak}$$
10. *Burst fall velocity (BFV)* was defined as the average slew rate from the coactivity at the peak C_i^{peak} to the coactivity at the end C_i^{end} of a burst (10% to 90%) across all *N* bursts of a recording:
$$BFV = \frac{1}{N}\sum_{i=1}^{N}\frac{C_i^{end} - C_i^{peak}}{t_i^{end} - t_i^{peak}}$$

Graph features

All graph features were calculated using the Brain Connectivity Toolbox [14].

Time-series features

1. *Regularity frequency (RF)* was defined as the frequency with the highest magnitude of the Fourier-transformed activity (Act)*:*

$$RF = argmax\left(\hat{f}(Act)\right)$$

2. *Regularity magnitude (RM)* was defined as the magnitude of the peak frequency of the Fourier-transformed activity:

$$RM = max\left(\hat{f}(Act)\right)$$

3. *Resonance fit (RFIT)* was defined as the exponential decay constant *d* of the fit through log10-transformed magnitudes *Y* of the regularity frequency harmonics *X*. The exponential model to fit is of the form:

$$Y = a \times e^{d \times X}$$

All other time-series features (*catch22*) were calculated using the toolbox published in [3]. Time-series features were calculated for each individual unit and the whole network from the binned activity (bin size: 100 ms).

HTRF assay

The Homogeneous Time Resolved Fluorescence assay (6FNSYPEG, Cisbio Bioassays, Codolet, France) was performed according to the manufacturer's instructions, and fluorescence emission at the acceptor (665nm) and donor wavelength (620 nm) were measured in a microplate reader (PHERAstar FSX, BMG LABTECH, Ortenberg, Germany). Total protein concentration was determined using the Pierce™ BCA Protein Assay Kit (23225, ThermoFisher). The ratios of acceptor and donor emission signals were calculated for each individual well and normalized by the total protein concentration. For each condition, three cultures (N=3) and three technical replicates were measured.

Immunocytochemistry

Cells were fixed using 8% paraformaldehyde solution (15714S, Electron Microscopy Sciences, Hatfield, USA) and blocked for 1 hour at room temperature (RT) in a blocking buffer containing 10% normal donkey serum (017-000-001, Jackson ImmunoResearch, West Grove, USA), 1% bovine serum albumin (BSA) (05482, Sigma-Aldrich), and 0.2% Triton X (93443, Sigma-Aldrich) in PBS (AM9625, ThermoFisher Scientific). Primary antibodies (**Table S9**) were diluted in blocking buffer and incubated overnight at 4°C. Samples were then washed three times with 1% BSA in PBS and incubated with the secondary antibody (**Table S9**) diluted in blocking buffer for 1 hour at RT. After three additional washes with PBS, DAPI was added for 2 min at RT (1:10000).

Image analysis

Images were acquired using the Opera Phenix Plus High-Content Screening System (HH14001000, PerkinElmer, Waltham, MA, USA), and the Harmony analysis software was used for quantification. Samples were analyzed by imaging six evenly spaced fields, each consisting of 3x3 images, resulting in 54 total images per sample at 40x magnification. Images were acquired as z-stacks, flat-field corrected, and converted to a 2D image using maximum intensity projection. Somatic quantification of α-synuclein (α-syn) and phospho-α-synuclein (p-syn) was performed by finding TH+ (avg. intensity > 50) nuclei (DAPI mask) and averaging the intensity of the target channel (α-syn or p-syn) in the selected area.
Statistical analysis was performed in GraphPad Prism 8 using an ordinary two-way ANOVA (factors: genotype and treatment) and the Tukey-Kramer test to compare all pairs of means, which accounts for multiple comparisons.

Supplemental references

1. Wang, X., A. Wirth, and L. Wang. *Structure-Based Statistical Features and Multivariate Time Series Clustering*. in *Seventh IEEE International Conference on Data Mining (ICDM 2007)*. 2007.
2. Mietus, J.E., et al., *The pNNx files: re-examining a widely used heart rate variability measure.* Heart, 2002. **88**(4): p. 378-380.
3. Lubba, C.H., et al., *catch22: CAnonical Time-series CHaracteristics.* Data Mining and Knowledge Discovery, 2019. **33**(6): p. 1821-1852.
4. Fulcher, B.D. and N.S. Jones, *hctsa: A Computational Framework for Automated Time-Series Phenotyping Using Massive Feature Extraction.* Cell Systems, 2017. **5**(5): p. 527-531.e3.
5. Müller, J., et al., *High-resolution CMOS MEA platform to study neurons at subcellular, cellular, and network levels.* Lab Chip, 2015. **15**(13): p. 2767-80.
6. Kriks, S., et al., *Dopamine neurons derived from human ES cells efficiently engraft in animal models of Parkinson's disease.* Nature, 2011. **480**(7378): p. 547-551.
7. Ronchi, S., et al., *Electrophysiological Phenotype Characterization of Human iPSC-Derived Neuronal Cell Lines by Means of High-Density Microelectrode Arrays.* Advanced Biology, 2021. **n/a**(n/a): p. 2000223.
8. Haenseler, W., et al., *Excess α-synuclein compromises phagocytosis in iPSC-derived macrophages.* Sci Rep, 2017. **7**(1): p. 9003.
9. Fernandes, Hugo J.R., et al., *ER Stress and Autophagic Perturbations Lead to Elevated Extracellular α-Synuclein in GBA-N370S Parkinson's iPSC-Derived Dopamine Neurons.* Stem Cell Reports, 2016. **6**(3): p. 342-356.
10. Fedele, S., et al., *Expansion of human midbrain floor plate progenitors from induced pluripotent stem cells increases dopaminergic neuron differentiation potential.* Scientific Reports, 2017. **7**(1): p. 6036.
11. McInnes, L., J. Healy, and J. Melville, *Umap: Uniform manifold approximation and projection for dimension reduction.* arXiv preprint arXiv:1802.03426, 2018.
12. Lee, E.K., et al., *Non-linear dimensionality reduction on extracellular waveforms reveals cell type diversity in premotor cortex.* eLife, 2021. **10**: p. e67490.
13. Blondel, V.D., et al., *Fast unfolding of communities in large networks.* Journal of statistical mechanics: theory and experiment, 2008. **2008**(10): p. P10008.
14. Rubinov, M. and O. Sporns, *Complex network measures of brain connectivity: Uses and interpretations.* NeuroImage, 2010. **52**(3): p. 1059-1069.
