# Supplementary material for: DeePhys: A machine learning–assisted platform for electrophysiological phenotyping of human neuronal networks
Source: Stem Cell Reports. 2024 Jan 25;19(2):285–98. doi: 10.1016/j.stemcr.2023.12.008 (PMC10874850; doi:10.1016/j.stemcr.2023.12.008)
Supplement: Document S2. Article plus supplemental information [file mmc2.pdf]

Stem Cell Reports
Resource
OPEN ACCESS

DeePhys: A machine learning–assisted platform for electrophysiological phenotyping of human neuronal networks

Philipp Hornauer,[1,*] Gustavo Prack,[1] Nadia Anastasi,[2] Silvia Ronchi,[1] Taehoon Kim,[1] Christian Donner,[3] Michele Fiscella,[1,4] Karsten Borgwardt,[1,5] Verdon Taylor,[6] Ravi Jagasia,[2] Damian Roqueiro,[1,2] Andreas Hierlemann,[1] and Manuel Schröter[1]

[1]Department of Biosystems Science and Engineering, ETH Zürich, 4056 Basel, Switzerland
[2]Roche Pharma Research and Early Development, Neuroscience and Rare Diseases, Roche Innovation Center Basel, F. Hoffmann-La Roche, 4070 Basel, Switzerland
[3]Swiss Data Science Center, ETH Zürich, 8092 Zürich, Switzerland
[4]MaxWell Biosystems AG, 8047 Zürich, Switzerland
[5]Swiss Institute of Bioinformatics, 1015 Lausanne, Switzerland
[6]Department of Biomedicine, University of Basel, 4031 Basel, Switzerland
*Correspondence: philipp.hornauer@bsse.ethz.ch
https://doi.org/10.1016/j.stemcr.2023.12.008

SUMMARY

Reproducible functional assays to study *in vitro* neuronal networks represent an important cornerstone in the quest to develop physiologically relevant cellular models of human diseases. Here, we introduce *DeePhys*, a MATLAB-based analysis tool for data-driven functional phenotyping of *in vitro* neuronal cultures recorded by high-density microelectrode arrays. *DeePhys* is a modular workflow that offers a range of techniques to extract features from spike-sorted data, allowing for the examination of functional phenotypes both at the individual cell and network levels, as well as across development. In addition, *DeePhys* incorporates the capability to integrate novel features and to use machine-learning-assisted approaches, which facilitates a comprehensive evaluation of pharmacological interventions. To illustrate its practical application, we apply *DeePhys* to human induced pluripotent stem cell–derived dopaminergic neurons obtained from both patients and healthy individuals and showcase how *DeePhys* enables phenotypic screenings.

INTRODUCTION

Neurological disorders are a leading cause of disability in aging societies (Feigin et al., 2020). However, despite considerable efforts, our understanding of fundamental pathomechanisms has remained incomplete. Moreover, many therapeutic approaches failed in clinical trials, and treatment options for patients remain limited. To close the apparent translational gap, there is an urgent need for new preclinical assays that enable researchers to investigate disease mechanisms in human tissue at scale and to evaluate drug candidates more effectively.

With the advent of human induced pluripotent stem cell (iPSC) technology, it is now possible to generate neural cells in a reproducible and scalable manner from any patient or healthy individual. Human iPSCs hold great promise to uncover some of the mechanisms and developmental pathways that give rise to neurological disorders *in vitro* (Dolmetsch and Geschwind, 2011). However, in contrast to genomic or proteomic analysis readouts, there is currently no established framework for a standardized functional characterization of human neurons.

Functional assays obtained with high-density microelectrode arrays (HD-MEAs) have recently gained momentum (Abbott et al., 2020; Müller et al., 2015), since they allow for the capture of the electrical activity of neurons at scale (several hundreds of neurons per HD-MEA), across extended development time (several months), and at high temporal (10–20 kHz sampling rate) and spatial resolution (subcellular details). Moreover, new multiwell HD-MEA system designs provide the throughput needed for high-content screenings.

Although some large-scale electrophysiological systems to screen human neurons *in vitro* have become available (e.g., the 48-well plates by Axion Biosystems), suitable tools to analyze the rich extracellular data of today's HD-MEAs (e.g., the 6-well plates by MaxWell Biosystems) have lagged behind. Most currently available toolboxes provide means to study either individual cells (Lee et al., 2021; Petersen et al., 2021) or network activity (Mahmud and Vassanelli, 2019), but they are often limited to certain aspects of the MEA data; for a review on currently available toolboxes, see Unakafova and Gail (2019).

Here, we introduce *DeePhys*, a novel analysis workbench that provides an integrated approach to extract and compare single-neuron and network-level features for functional phenotyping of human neurons. We outline the different processing modules of *DeePhys* and apply those in proof-of-concept experiments to iPSC-derived neuronal cultures obtained from healthy controls and patients with Parkinson disease (PD). While the primary application example of *DeePhys* is the functional phenotyping of iPSC-derived human dopaminergic (DA) neuron

This is an open access article under the CC BY license (http://creativecommons.org/licenses/by/4.0/).

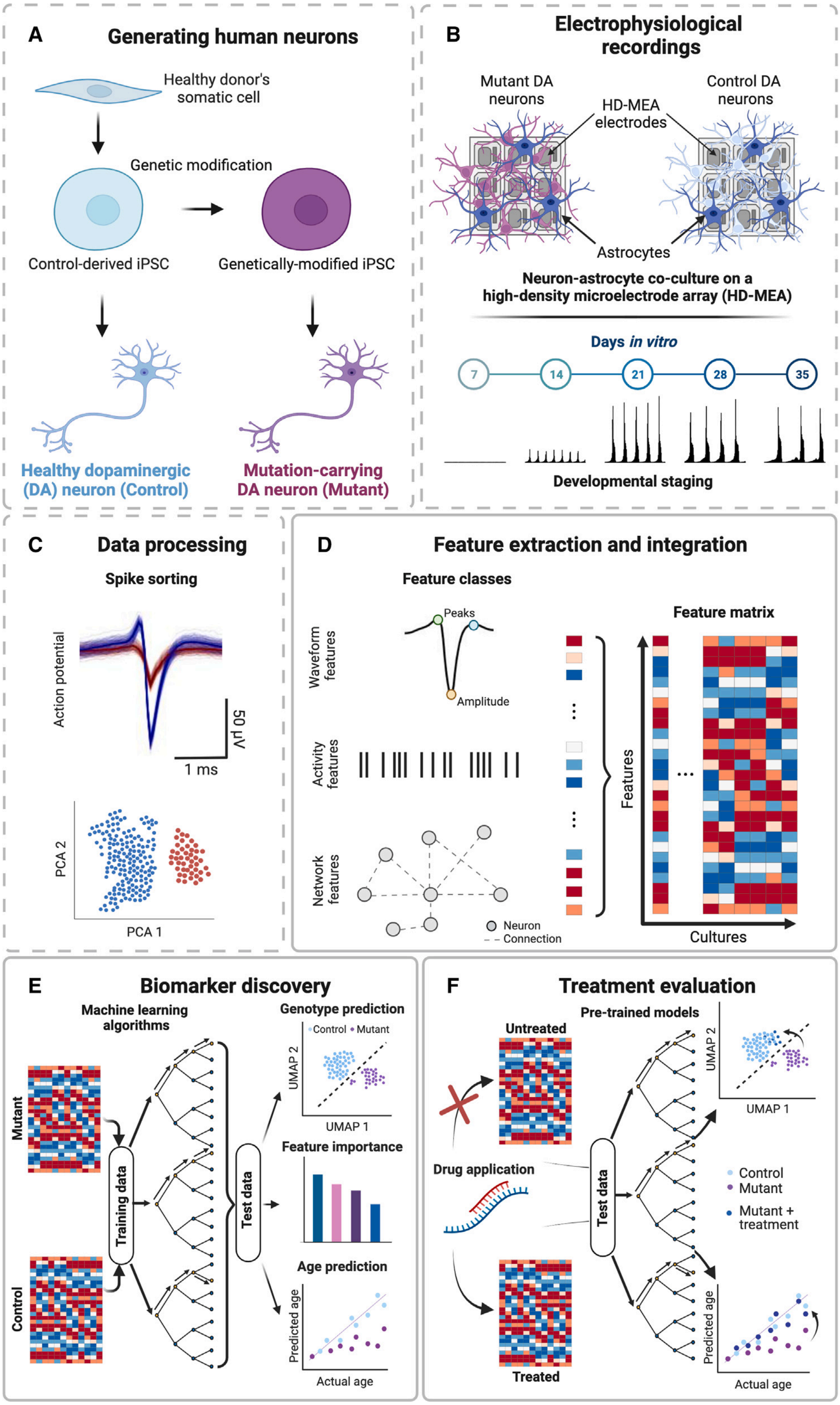

(legend on next page)

cultures harboring the A53T point mutation, we also probe how results can be generalized to other lines.

We first demonstrate that *DeePhys* uncovers reliable features to predict healthy and mutant DA neuron lines and their developmental stage. Moreover, our results indicate that the high-dimensional feature space allows for evaluating treatment interventions—here, exemplified by the application of locked nucleic acid (LNA)–mediated downregulation of α-syn expression. Finally, we show how *DeePhys* can be used to identify the phenotypes of more heterogeneous neuronal cultures and how acute drug challenges can be used to refine these results. We expect that the obtained insights will add an important layer to the characterization of human cellular models of neurological diseases *in vitro*.

RESULTS

DeePhys processing modules

DeePhys is composed of four analysis modules. First is a preprocessing module, which allows for quality control of the spike-sorted input data and the extraction of a large range of extracellular features (Figure 2). Second is a feature-integration and phenotyping module that combines the inferred extracellular features at individual time points or across development to derive robust electrophysiological phenotypes (Figure 3). This module relies on machine learning algorithms to probe the reliability of the inferred phenotypes and identifies the most predictive features, which can be used to find potential biomarkers. The third module provides means to assess the impact of acute or chronic pharmacological interventions on the inferred phenotypes (Figure 4). Finally, the fourth module enables feature-based single-cell clustering for analyses at the level of putative cell types (Figure 5). A schematic overview of the *DeePhys* pipeline is given in Figure 1, and examples of HD-MEA inferred single-neuron and network-level features are shown in Figure 2 (see Table S1 for a full list). The main functions are listed in Table S2. The MATLAB code to run *DeePhys* is open source and available on a public code repository (see Experimental procedures), where we also provide online tutorials and test data.

Inferring extracellular electrophysiological features

We previously demonstrated the feasibility of recording from human neurons using HD-MEAs and of profiling them using standard spike-train statistics (Ronchi et al., 2021). Here, we significantly expanded the range of features and derived >50 quantitative measures from spike-sorted HD-MEA recordings. Following a quality control step to assess the spike-sorting results, we inferred two main classes of features: single-cell metrics and network-level metrics (Figure 2). Briefly, the single-cell metrics consisted of eight action potential waveform metrics (later referred to as waveform features; Figures 2A and 2B) and four metrics that describe spike-train dynamics (spike time features). There is a large body of evidence that waveform and spike time metrics are indicative of putative cell types (Trainito et al., 2019) and their laminar location *in vivo* (Lee et al., 2021).

At the network level, we quantified functional-connectivity features (graph features) and the intrinsic network-burst activity (burst features; Figures 2D and 2E). Previous studies have linked differences in these features to synaptic functioning and, more generally, network maturation (Wagenaar et al., 2006). Finally, we also derived time series features, which were inferred from the binned spiking activity of individual units as well as the whole network (Figures 2C and 2F). These features were obtained in a previous study (Lubba et al., 2019) and have been used for time series classification tasks (Fulcher and Jones, 2017). We hypothesized that applying and combining single-cell and network-level features would facilitate the functional phenotyping of neuronal networks.

Characterizing human iPSC-derived midbrain DA neuronal networks

As a proof of principle for the *DeePhys* toolbox, we used two human cell lines of commercially available, purified, and fully differentiated midbrain DA neurons. The protocol to differentiate these DA neurons from iPSCs was based on work by the Studer lab (Kriks et al., 2011). The two lines comprised a healthy control line and an isogenic mutant line, with the A53T site-specific mutation introduced into the *SNCA* gene. The A53T variant has one of the strongest effects on the initiation and spreading of α-syn aggregations (Flagmeier et al., 2016) and results in an autosomal dominant form of familial PD (Polymeropoulos et al., 1997). Both lines were cocultured with human iPSC-derived astrocytes and plated on HD-MEAs, as described previously (Ronchi et al., 2021). Weekly HD-MEA recordings of the emerging neuronal activity started 7 days after

Figure 1. Schematic of the *DeePhys* analysis pipeline

(A and B) Schematic outlining the generation of neurons from human iPSCs (A), and the plating and recording of human neurons on HD-MEAs (B).

(C–F) The *DeePhys* pipeline starts with data that has been spike sorted (C) and consists of several modules: the HD-MEA feature extraction and integration (D), the feature evaluation (E), and the assessment of treatment interventions (F).

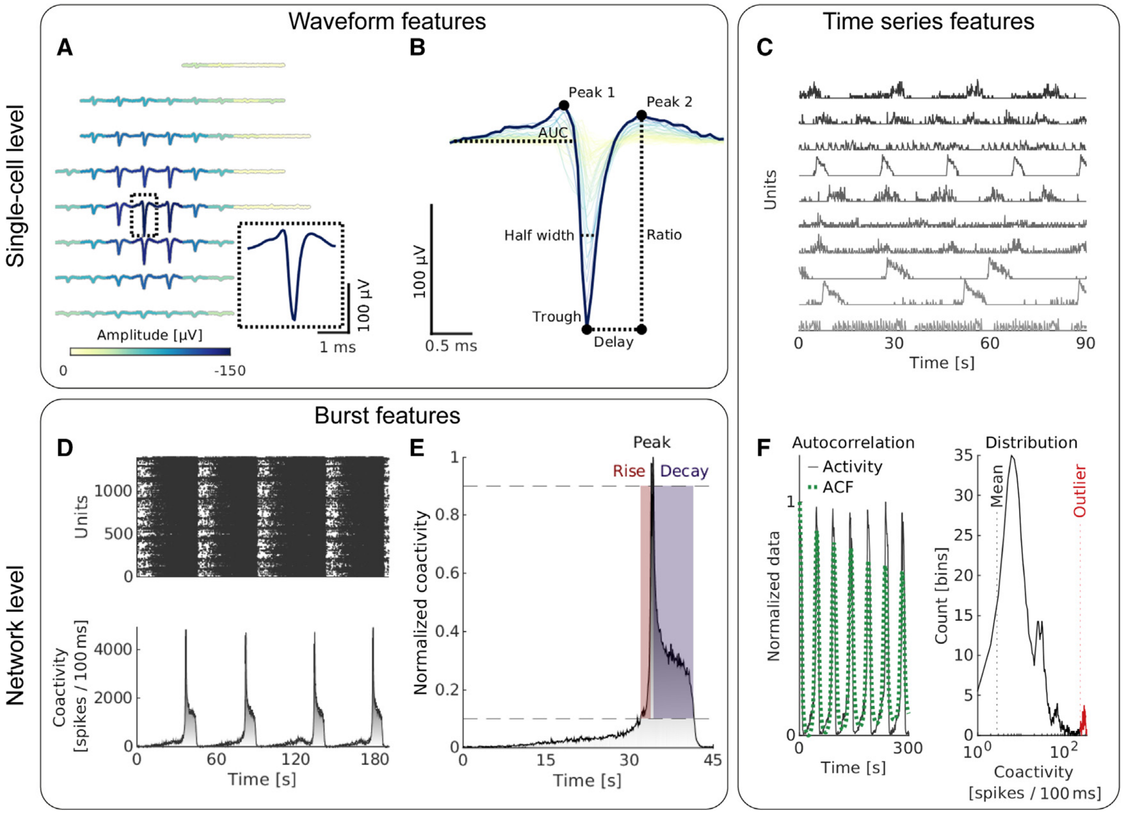

Figure 2. Quantifying neuronal activity on HD-MEAs

Example features describing neuronal activity at the single-cell and network level.

(A) The spike-triggered electrical footprint (EF) of a single unit on the HD-MEA.

(B) The largest negative amplitude waveform of an EF was used to infer waveform features. The coloring indicates the respective trough amplitudes.

(C) Binned spike trains of individual units were used for the time series feature extraction.

(D) Raster plot with activity recorded from a DA neuronal culture at week 5. Dots represent spike-sorted action potentials. (Bottom) The corresponding binned coactivity of all of the units.

(E) Magnification of a single network burst, highlighting its rise and decay phase.

(F) Examples of time series features, such as the autocorrelation function (ACF) and the distribution of the binned coactivity, as inferred from single-cell (C) and network (D) activities.

the plating. Figure 2B shows representative network-activity plots for both WT and A53T DA neuron cultures.

Using immunocytochemical (ICC) analysis (N = 3 cultures per cell line), we confirmed the presence of DA neurons and astrocytes in control experiments by staining for tyrosine hydroxylase (TH), microtubule-associated protein 2 (MAP2), and glial fibrillary acidic protein (GFAP) at day *in vitro* 21 (Figures 2A and S1). Both wild-type (WT) and A53T cultures showed robust outgrowth and neuritic arborization of TH^+ neurons and a wide coverage of $GFAP^+$ astrocytic processes. Quantifications of TH^+ and $MAP2^+$ nuclei revealed subtle differences in the composition of both lines (Table S3). A comparison of α-syn levels between genotypes is provided in the section Assessment of pharmacological perturbations.

DeePhys allows accurate predictions of DA neuron culture type and age

Following the quality control and feature extraction steps, we trained random forest (RF) classifiers for each metric. The resulting matrix ($N_{cultures} \times N_{recordings}$) was then normalized and used as input to predict the culture type (WT versus A53T); leave-one-out cross-validation (CV) was applied to obtain accuracy values for each feature. In addition, we calculated the permutation predictor importance to infer the relative predictor importance at each recording time point (i.e., the week *in vitro*). Statistical differences in the development of features were assessed by linear mixed-effects models (LMMs). Table 1 provides an overview of the features used throughout the paper.

Table 1. Feature names and descriptions

Feature	Feature class	Description
MIB	Burst	Mean interburst interval
MFT	Burst	Mean time from the peak to the end of a burst
GEC	Graph	Global efficiency calculated on cross-correlogram (CCG)-inferred connectivity graph
DEC	Graph	Network density based on CCG graph
CVI	Spike time	Coefficient of variation of the interspike interval
MIS	Spike time	Mean interspike interval
RF	Time series	Peak frequency of Fourier power spectrum
RM	Time series	Magnitude of the corresponding RF feature
EAF	Time series	First 1/e crossing of autocorrelation function
AMI	Time series	Automutual information
SFR	Time series	Proportion of slower timescale fluctuations that scale with linearly rescaled range fits
LPF	Time series	Total power in the lowest fifth of frequencies in the Fourier power spectrum
MEF	Time series	Mean error from a rolling 3-sample mean forecasting
PAM	Time series	Longest period of consecutive values above the mean
EFD	Time series	Exponential fit to successive distances in two-dimensional embedding space
CCD	Time series	Change in correlation length after iterative differencing
TCT	Time series	Trace of covariance of transition matrix
SES	Time series	Shannon entropy
CFS	Time series	Centroid of the Fourier power spectrum
SFD	Time series	Proportion of slower timescale fluctuations that scale with detrended fluctuation analysis
MD5	Time series	Mode of *Z* scored distribution (5-bin histogram)
MD10	Time series	Mode of *Z* scored distribution (10-bin histogram)
FMA	Time series	First minimum of autocorrelation function
FMI	Time series	First minimum of the automutual information function
TRS	Time series	Time-reversibility statistic
TEA	Time series	Time intervals between successive extreme events above the mean
RFT	Time series	Exponential fit of successive peak frequencies in the Fourier power spectrum
PDE	Time series	Proportion of successive differences exceeding 0.04 SD

Subset of *DeePhys* features and their respective abbreviations as used throughout the paper. Time series features that were inferred at the single-cell or the network level are denoted by a leading s or n, respectively (e.g., sRM refers to the single-cell regularity magnitude). See Table S1 for a complete list of features.

We found that WT and A53T DA neuron cultures showed marked differences across single-cell and network-level metrics (Figure 3). Single-cell features differed consistently between genotypes at the activity level, particularly metrics that describe the temporal regularity of DA neuron firing. The single-cell regularity frequency (accuracy: 0.96, $p < 0.01$, LMM) and regularity magnitude (accuracy: 0.92, $p > 0.05$, LMM) were the two most predictive features among the time series feature group (Figure 3D). Spike time features, such as the coefficient of variation of the interspike interval (CVI, accuracy: 0.92, $p > 0.05$, LMM), were also highly predictive (Figure 3D). Waveform features were less predictive for the classification of cell lines (maximum accuracy value of 0.68).

Network-level features also showed clearly distinct phenotypic profiles for WT and A53T cultures (Figure 3C, lower panel), which became apparent early during development. Although A53T cultures featured high levels of correlated spontaneous activity 1 week after plating, no bursts were detected in WT cultures at that time. The time series feature group was most predictive at the network level (e.g., periodicity measure, accuracy: 1.00, $p < 0.01$, LMM). Various network-level burst metrics were also highly predictive, reaching accuracy values of up to 0.92 (e.g., the mean interburst interval [MIB], $p > 0.05$, LMM). The waveform and graph-feature groups showed the lowest accuracy across development (accuracy <0.58 and <0.60; Figure 3F). The network-feature group displayed a reduction in accuracy in week 2, consistent with a crossover of some developmental feature trajectories around that time. The aggregation of features across

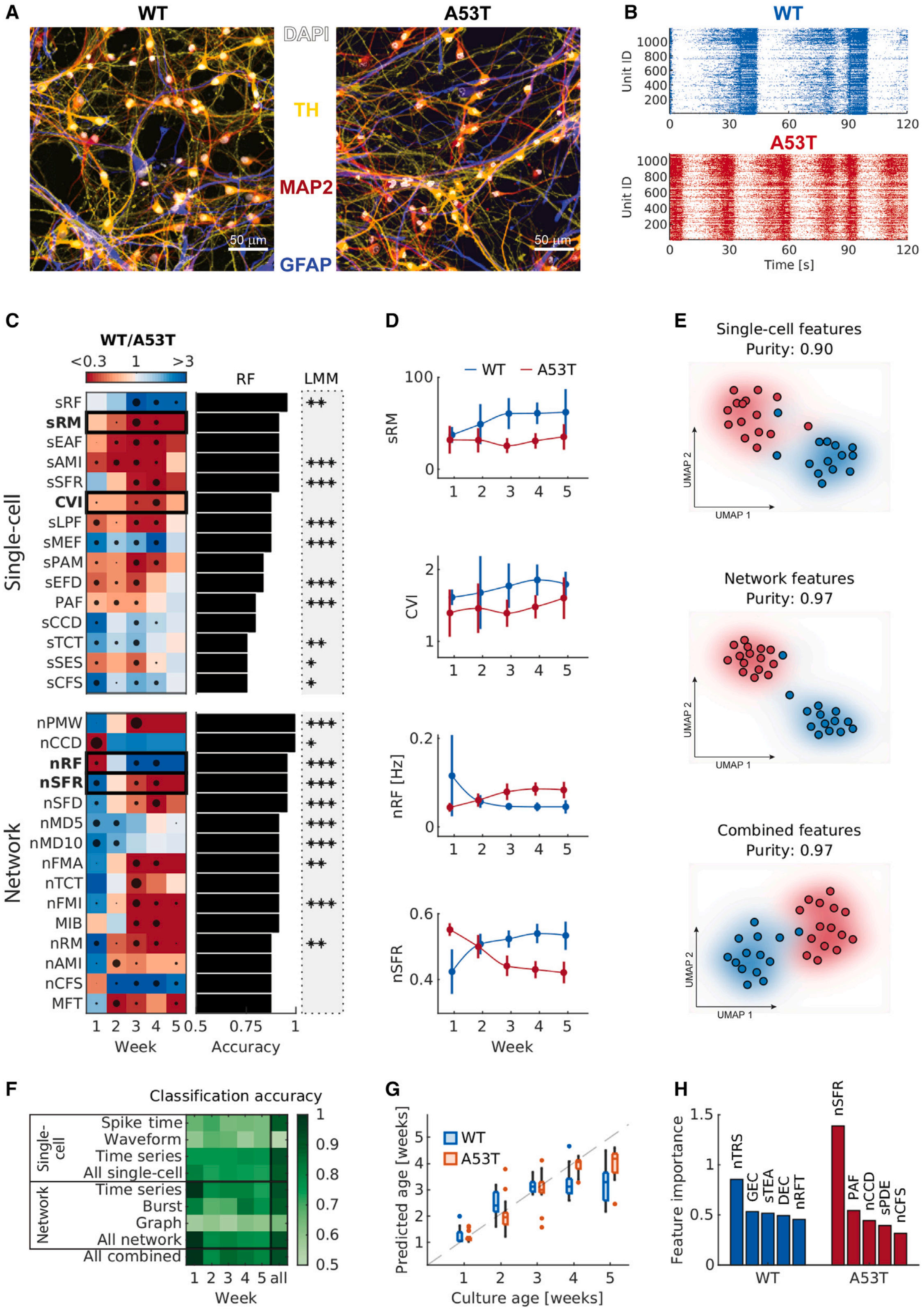

(legend on next page)

development improved the classification performance (Figure 3F).

Uniform manifold approximation and projection (UMAP) analyses of single-cell or network-level features, or a combination of both feature classes, also indicated robust differences between WT and A53T cultures (Figure 3E). Visual inspection allowed a clear distinction of both lines in the two-dimensional (2D) UMAP space. This result was corroborated by high cluster purity values (separability of clusters) for both network-level (0.97) and single-cell features (0.90); combining network and single-cell features did not further improve the separability (0.97).

Finally, we used RF regression models to probe whether features inferred by the *DeePhys* pipeline would let us predict the age of DA neuron cultures (Figure 3G). We found that age predictions remained accurate until week 3 for WT cultures (mean average error [MAE] = 2.4 days). After 3 weeks *in vitro*, the predicted age plateaued (MAE = 9.0 days). The age of A53T cultures was accurately predicted until week 4 (MAE = 2.3 days), but then underestimated at week 5 (MAE = 4.3 days). We found that the features driving the age predictions differed significantly between WT and A53T cultures (Figure 3H). Together, our results show that the *DeePhys* pipeline allowed for robust functional phenotyping of WT/A53T DA cultures based on features extracted from spike-sorted HD-MEA recordings.

Assessment of pharmacological perturbations

Next, we used *DeePhys* to map out the effects of a pharmacological intervention on WT/A53T functional phenotypes. Specifically, we studied the alterations following chronic application of a LNA to reduce α-syn expression. Although reducing α-syn has been discussed as a treatment option for PD (Fields et al., 2019), the main goal of the current experiment was to monitor the effect of such a reduction on the electrophysiological profiles obtained by *DeePhys*.

We confirmed the efficacy of the LNA construct by measuring α-syn levels in WT/A53T cultures that were either untreated, treated with a nontargeted LNA (ntLNA), or treated with an *SNCA*-targeting LNA. This control experiment was performed on coverslips at week 3 using a homogeneous time resolved fluorescence (HTRF) assay (N = 3 cultures per condition; Table S4). Results indicated a significant effect of the LNA treatment on total α-syn levels (two-factor ANOVA, $p < 0.001$), but no significant difference between genotypes ($p = 0.820$). Post hoc multiple comparisons tests (Tukey-Kramer test) showed a significant decrease in α-syn in LNA-treated WT and A53T cultures compared to untreated controls (both $p < 0.001$; Figure 4B); α-syn levels in cultures treated with ntLNA did not differ significantly from levels obtained in untreated controls ($p > 0.999$). ICC analysis (N = 3 cultures per condition) of somatic α-syn levels confirmed these results (two-factor ANOVA, treatment: $p < 0.001$, genotype: $p = 0.116$; Table S5). Phosphorylated α-syn (*p*-syn) levels, however, were significantly increased in A53T DA neurons (two-factor ANOVA, $p = 0.001$; Table S6).

We then applied the *DeePhys* pipeline and asked whether downregulating α-syn via LNA treatment altered the electrical activity of DA cultures (Figure 4C). We focused our analysis on the 10 single-cell and network-level features with the highest predictive power in distinguishing between WT and A53T (see Figure 3C). For this analysis, we pooled untreated and ntLNA-treated cultures to increase statistical power (see Figure S2 for a comparison of ntLNA-treated and untreated culture profiles). Our results indicate that LNA-mediated α-syn downregulation led to distinct alterations, such as shorter but more frequent bursts. Moreover, while the network regularity frequency (nRF) typically decreased throughout development in WT

Figure 3. A53T DA neuron cultures exhibit age-dependent alterations at the single-cell and network level

(A) ICC staining of WT (left) and A53T (right) human iPSC-derived DA neurons, cocultured with human astrocytes at week 3. See also Figure S1.

(B) Example raster plot with activity recorded from a WT and an A53T DA neuronal culture at week 5.

(C) Heatmaps indicating differences between genotypes at a given recording time point (top: single-cell features, bottom: network features). The horizontal bar plots display the accuracies of RF classification models, trained with the respective features as input. The size of the black dots within each panel of the heatmap indicates the relative predictor importance at the respective time point. Asterisks indicate the significance levels of the corresponding LMMs (*$p < 0.05$, **$p < 0.01$, ***$p < 0.001$; $N_{WT} = 18$, $N_{A53T} = 19$ cultures). See Table 1 for more detailed feature descriptions.

(D) Example developmental trajectories of 4 highly predictive features (mean ± SD values; $N_{WT} = 18$, $N_{A53T} = 19$ cultures).

(E) UMAP dimensionality reduction, based on either single-cell or network features, or a combination of both feature classes, demonstrates the separability of WT and A53T cultures. The cluster purity values quantify the result of k-means clustering analyses (k = 2).

(F) Heatmap depicting the RF classification accuracy by culture age and input feature group ($N_{WT} = 14$, $N_{A53T} = 15$ cultures).

(G) Age prediction using RF regression analysis (boxes show the median, lower, and upper quartiles; whiskers indicate the nonoutlier minimum and maximum values; dots indicate outliers).

(H) Bar graph depicting the features with the highest predictor importance inferred from the RF age prediction. See Table 1 for more detailed feature descriptions.

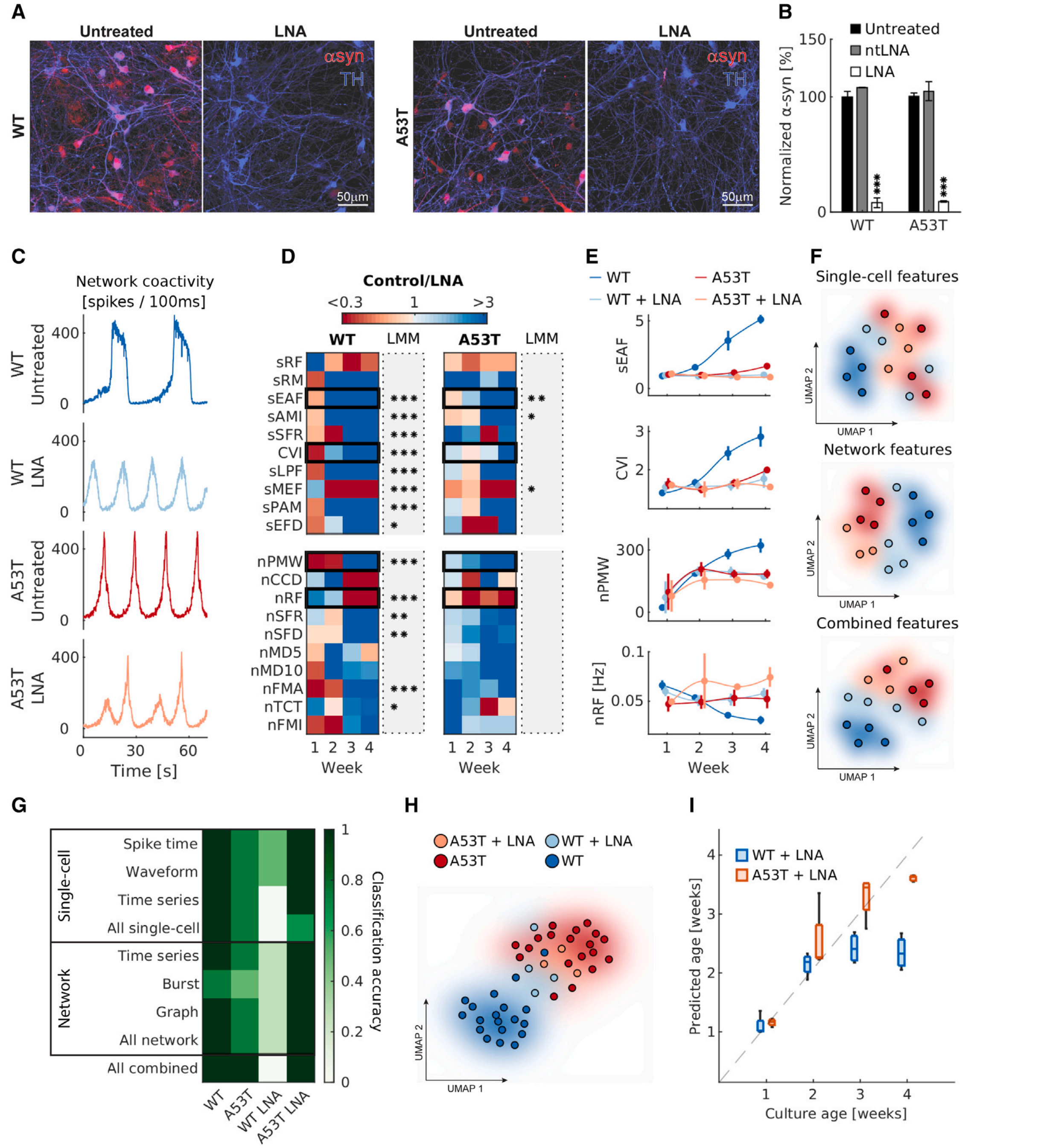

Figure 4. Downregulation of α-syn alters electrophysiological phenotypes and culture development

(A) Representative staining for α-syn and TH of WT/A53T DA neuronal cultures at week 3.

(B) Quantification of α-syn levels using an HTRF assay. Values are normalized by untreated WT levels (Tukey-Kramer test, ***$p < 0.001$; N = 3 cultures with 3 technical replicates).

(C) Representative coactivity plot of LNA-treated and untreated WT/A53T DA neuronal cultures at week 5.

(D) Heatmaps depicting the relative feature differences of the 10 most predictive single-cell (top) and network features (bottom) as shown in Figure 3C. Asterisks indicate the significance of the respective LMMs (*$p < 0.05$, **$p < 0.01$, ***$p < 0.001$; N = 20 cultures).

(E) Exemplary developmental trajectories of 4 highly predictive features (mean ± SD values).

(F) UMAP dimensionality reduction, based on either single-cell or network features, or a combination of both feature classes.

(legend continued on next page)

cultures, LNA treatment reversed this trend (Figure 4E, lowest panel). A53T cultures exhibited a similar development, as LNA treatment increased the nRF metric. Most metrics showed similar trends in both lines, but LNA-induced differences were predominantly significant in WT cultures (Figure 4D). UMAP analyses visualized the robustness of the LNA effect because LNA-treated WT cultures mostly colocalized with A53T cultures across feature classes (Figure 4F).

We then applied the RF classifier trained on the cultures of our previous experiment (Figure 3) to the cultures of the LNA experiment. Results for predicting the phenotype of untreated WT and A53T cultures indicated that most feature groups generalized very well (Figure 4G), and even perfect classification was achieved by combining all of the feature classes ("all combined"). This validation allowed us to assess the effect of the LNA treatment by calculating the classification accuracy of LNA-treated WT and A53T cultures. The accuracy of the prediction of LNA-treated WT cultures was low across all of the feature groups, but remained high for LNA-treated A53T cultures (Figure 4G, WT LNA+A53T LNA). This observation is consistent with the UMAP results (Figure 4H) because LNA-treated WT cultures colocalized visually with untreated A53T cultures from the first experiment (see results from Figure 3).

Finally, we assessed the impact of α-syn downregulation on overall culture development by predicting the age of LNA-treated cultures using the RF regression models trained on their respective controls. Results indicated that the development of LNA-treated WT cultures plateaued earlier (after 2 weeks *in vitro*), whereas A53T cultures remained largely unaffected by the LNA treatment (Figure 4I).

Applying *DeePhys* to heterogeneous human DA neuron cultures

Many of the current neuron differentiation protocols give rise to heterogeneous cultures (i.e., a diverse set of neuron types per culture), which may increase the variability between cell lines and affect functional phenotyping (Volpato et al., 2018).

Here, we set out to probe whether *DeePhys* could be used to identify electrophysiological phenotypes at the cellular level and to infer putative cell clusters. We therefore recorded from two heterogeneous lines obtained from differentiated iPSC-derived midbrain floor plate progenitors: a cell line obtained from a healthy control subject and another from a PD patient with the *SNCA* triplication (SNCA). Cultures were prepared using a previously published protocol with a proportion of DA neurons of approximately 8% at week 3 (Fedele et al., 2017).

Results obtained with *DeePhys* indicated clear differences between homogeneous and heterogeneous cell lines, as well as between healthy controls and PD-associated mutations (A53T, SNCA; Figure S3). Given these differences, we next asked whether single-cell HD-MEA features could be used to parse out the different cellular compositions of homogeneous or heterogeneous cultures. We performed single-cell clustering on heterogeneous control cultures (Louvain clustering), using either action potential waveforms (Figure 5A) or a combination of waveforms and activity features (Figure 5B). An RF classifier was then trained on these clusters and used to quantify the importance of individual features (Figures 5A and 5B, panels at right) and to infer putative cell type compositions of the cultures (Figures 5C and 5D). We found that all of the clusters were present among the spike-sorted units of the heterogeneous cell lines, and one dominating cluster consistently made up >70% of all of the units in the homogeneous lines, irrespective of the genotype or treatment condition. These results indicate that *DeePhys* enabled reliable cell-cluster assignments despite pronounced differences at the network level (Figures 4C and S3).

Finally, we probed whether an acute pharmacological perturbation could be used to further refine the single-cell clustering. Previous research has demonstrated that the dopamine receptor D2 agonist quinpirole (QP) reduces DA neuron firing rates (FRs), so we expected cell cluster–specific responses after QP addition (Dagra et al., 2021). We therefore performed Louvain clustering on the UMAP graph generated from heterogeneous, untreated WT baseline recordings and mapped the FR change after QP (10 μM) treatment (Figure 5E; an FR decrease is marked with a black dot). Units in clusters 3 and 5 predominantly decreased their FR (60% of the units in these clusters), whereas in all of the other clusters such a decrease was only observed for approximately 30% of the units (Figure 5F). Some of these clusters persisted when analyzing the QP response, indicating a correlation between baseline activity and QP response (Figure S4). Moreover, results indicated that the obtained QP response

(G) Heatmap depicting the RF classification accuracy of control cultures ($N_{WT} = 4$, $N_{A53T} = 5$) and LNA-treated cultures ($N_{WT} = 4$, $N_{A53T} = 3$) by input feature group. The RF classifier was trained on all of the cultures of the experiment displayed in Figure 3.
(H) Projection of LNA-treated cultures into the UMAP space of untreated cultures.
(I) Age prediction of LNA-treated cultures using an RF regression model trained on untreated cultures (boxes visualize the median, lower, and upper quartiles; whiskers indicate nonoutlier minimum and maximum values; dots indicate outliers).

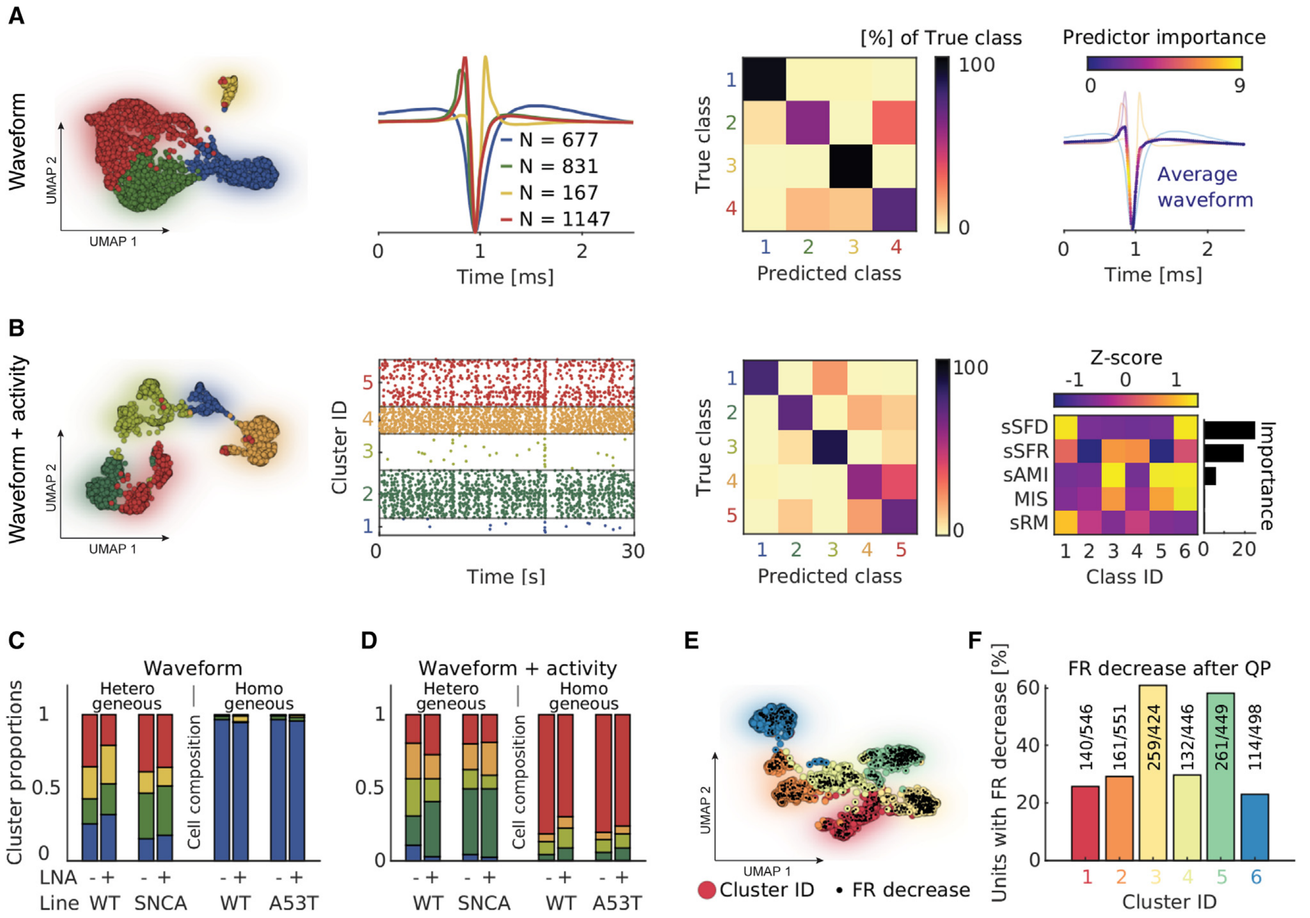

Figure 5. Characterizing electrophysiological phenotypes at the single-cell level

Inferring reliable single-cell clusters in heterogeneous cultures (N = 5 cultures) based on action potential waveforms (A) or a combination of action potential waveforms and spike activity (B). From left to right: the UMAP embeddings (colors indicate the cluster ID from Louvain clustering); the average waveforms (A) and representative spike trains (B) of the corresponding clusters; the confusion matrices of RF classifiers trained on the inferred clusters; and the predictor importance values of waveform- and activity-based classifiers.

(C) Predicted cluster compositions using the classifier from (A).

(D) Predicted cluster compositions using the classifier from (B).

(E) UMAP plot of the single-cell baseline activity; colors indicate the cluster ID from Louvain clustering. Black dots indicate a unit with a reduction in FR upon QP addition (N = 2,914 units from 5 cultures).

(F) Ratio of units that display a FR decrease upon QP addition.

clusters may facilitate the phenotyping of heterogeneous cell lines (Figure S4).

DISCUSSION

The main goal of this study was to introduce and validate *DeePhys*, a new open source analysis pipeline to extract multiparametric information from spike-sorted electrophysiological recordings. The *DeePhys* pipeline is modular and easily scalable, it requires only minimal manual intervention, and it can be used as a screening tool to investigate different human cellular models. We applied *DeePhys* to purified and heterogeneous human iPSC-derived DA neuronal cultures, maintained over several weeks on HD-MEAs, and demonstrated its utility in systematically studying neuronal maturation and pharmacological perturbations.

For the first experiment, we used the *DeePhys* pipeline to identify descriptive phenotypic differences between A53T mutant and isogenic control DA neuron cultures across development. We found that both cell lines can be reliably classified according to their electrophysiological phenotypes, with network-level metrics representing the more informative features. Moreover, changes related to the regularity of neuronal activity were reliably detected in both

single-cell and network features. A53T cultures showed an earlier onset of spontaneous synchronized activity and smaller bursts at a higher rate later in development. The differences in burst dynamics could provide a link to a previous study, which found that mutant α-syn interferes with vesicle recycling and the regulation of the recycling pool (Xu et al., 2016). Both processes are essential in maintaining synaptic activity over prolonged periods. Alterations in the functionality of α-syn due to the A53T mutation may therefore alter the duration and frequency of network bursts. Also, mitochondrial defects and bioenergetic deficits, caused by the A53T mutation, may reduce the length of high-activity periods because the energy demand of prolonged spiking cannot be met (Ryan et al., 2013; Zambon et al., 2019).

Next, we showed how *DeePhys* can be used to assess the impact of chronic pharmacological interventions on the functional phenotypes. The strongly decreased α-syn levels in DA neurons after LNA treatment resulted in an electrophysiological phenotype similar to that of A53T mutant cultures. This finding was confirmed by applying a pretrained RF model on LNA-treated cultures, which classified all LNA-treated cultures as A53T. However, most phenotypical features, such as an increased burst rate, were even more pronounced for the LNA condition. This result proved true regardless of the genotype, because WT and A53T cultures were affected similarly by the LNA-induced reduction in α-syn levels. α-Syn has been reported to be involved in the maintenance of the synaptic vesicle (SV) pool size through vesicle recycling and inhibition of inter-SV trafficking (Scott and Roy, 2012; Sun et al., 2019). In addition, α-syn was shown to cluster SVs and to attenuate the release of neurotransmitters (Wang et al., 2014). A reduction in α-syn levels may, therefore, restrict the SV pool size before bursts and the recycling rate during network bursts, which could result in shorter, low-frequency bursts.

Furthermore, we demonstrated how *DeePhys* can be used to bridge the gap between single-cell and network phenotypes by extracting patterns from the action potential waveform shape and activity of individual cells. We found that distinct unit clusters could be detected for iPSC-derived heterogeneous neuronal lines (WT, *SNCA* triplication), whereas one cluster was consistently dominant for the DA neuron–enriched homogeneous lines. Interestingly, the putative cell cluster composition was consistent across cell lines and treatment conditions, despite pronounced differences in their network activity. Although this finding may indicate that the predominant cluster corresponds to the DA neuron population of the more homogeneous cultures, this will have to be probed in future studies with ground-truth data. Such validation seems necessary, as the spike waveform of a unit varies not only by its cell type but also by the location and the distance and angle of the cell to the respective HD-MEA electrodes.

Lastly, we showed how *DeePhys* can be used in combination with acute pharmacological perturbations (QP) to detect differential responses of individual unit clusters. In line with previous research (Dagra et al., 2021), we found clusters that predominantly reduced in FR after QP application. In addition, we found that baseline activity clusters could be partially mapped to the respective QP responses, and that the resulting clusters could be used to characterize the cell line–specific QP response across the whole culture. In future studies, this approach could be expanded by incorporating more perturbation responses or other modalities, such as RNA sequencing data, to further validate the inferred clusters.

Our results show that *DeePhys* provides an easy-to-use, scalable, quantitative analysis platform for functional phenotype screening and for addressing important biomedical questions in the development of new treatments. Its compatibility with SpikeInterface (Buccino et al., 2020) allows *DeePhys* to be used with most popular spike-sorting algorithms, and its modular organization facilitates the integration of new input formats and the addition of other feature groups (e.g., local field potential data). We are confident that *DeePhys* and the analysis approach presented here have great potential to add to a better functional characterization of a wide range of cellular models of neurological diseases.

EXPERIMENTAL PROCEDURES

Resource availability

Corresponding author

Further information and requests for resources and reagents should be directed to and will be fulfilled by the corresponding author, Philipp Hornauer (philipp.hornauer@bsse.ethz.ch).

Materials availability

This study did not generate new unique reagents.

Data and code availability

The code to run *DeePhys* and to reproduce the figures is available at https://github.com/hornauerp/DeePhys.git. The raw datasets have not been deposited in a public repository due to the excessive file size (>5 TB) but are available from the corresponding author upon reasonable request. The preprocessed datasets are available at https://doi.org/10.5281/zenodo.7876371.

Cell lines

Homogeneous cultures

Fully differentiated human iPSC-derived DA neurons carrying a heterozygous A53T mutation (C1112, FUJIFILM Cellular Dynamics International, Madison, WI) and an isogenic control line (C1087) were cocultured with astrocytes (R1092) as previously described (Ronchi et al., 2021).

Heterogeneous cultures

Human iPSC lines from apparently healthy controls and PD patients carrying a triplication of the *SNCA* gene were differentiated using a recently established protocol (Fedele et al., 2017). On day 20 of differentiation, cultures were dissociated, and 120,000 cells were plated onto each HD-MEA.

All medium formulations and methods for generation, plating, and culture of iPSC-derived DA neurons, ICC (see Table S9 for a list of antibodies), and image analysis are detailed in the supplemental experimental procedures.

HD-MEA recordings

Neuronal cultures were recorded for 15 min weekly, as previously described (Ronchi et al., 2021), using the HD-MEAs MaxOne and MaxTwo (MaxWell Biosystems, Zurich, Switzerland). The used HD-MEAs feature 26,400 electrodes with a 17.5-μm pitch, 1,024 readout channels, and a 20-kHz (MaxOne)/10-kHz (MaxTwo) sampling rate (Müller et al., 2015). Electrode selection was performed based on an activity scan, and the electrodes with the highest FR were selected for the network scan. Spike sorting was performed using Kilosort 2.5 (Stringer et al., 2019) using parameters tailored to the dataset at hand (Table S7). The spike-sorted data underwent quality control, considering the overall activity of units (>0.01 Hz), the refractory period violations (<2%), and irregularities in the waveform shape. The experiments involving LNA and QP are detailed in the supplemental experimental procedures.

Feature extraction

Single-cell features

Single-cell features were extracted from all of the units and then averaged to obtain one representative value for each culture (Figures 3 and 4). The action-potential waveform features were derived from the spike-triggered multielectrode waveform signal (template), as generated during the spike sorting (Figure 2A). Single-cell waveform features were extracted from the electrode with the largest negative waveform amplitude (Figure 2B).

The activity features included spike time features, which were inferred from the spike times of individual units. Time series features (Figure 2C) were inferred from the binned activity (bin size: 100 ms). Most time series features were adapted from a recent publication on time series classification (*catch22*, version 0.4.0; Lubba et al., 2019).

Network features

The network features describe the activity of the entire network after aggregating the activity of all of the spike-sorted units of a culture (Figures 2D and 2E).

To infer burst features, we detected network activity bursts using a previously introduced method (Bakkum et al., 2014). Since cultures were tracked across development, we adapted the algorithm to account for changes in overall activity.

Graph features were inferred from functional connectivity graphs that were calculated using either a cross-correlogram-based approach (English et al., 2017) or the spike time tiling coefficient (Cutts and Eglen, 2014).

Network time series features were calculated from the binned activity (bin size: 100 ms) across all of the spike-sorted units.

Statistical analysis

We applied LMMs models of the form $Y \sim 1 + \text{Genotype} \times \text{Time} + (1|\text{Subject})$ to compute statistical significance in the developmental trajectories of individual features. We concatenated all of the feature values across recording time points for each culture. The restricted maximum likelihood (REML) estimation was used as a fitting method (MATLAB function: *lme = fitlme(input_table, formula, 'FitMethod','REML', 'DummyVarCoding', 'effects')*). The Satterthwaite approximation was applied to compute approximate degrees of freedom (MATLAB function: *anova(lme,'DFMethod','satterthwaite')*). The resulting p values were adjusted for the number of comparisons/features using the Bonferroni correction.

Machine learning methods

The input matrix for the RF classifier was obtained by concatenating individual features ($N_{cultures} \times N_{recordings}$) or all of the features of a feature group across the selected recording time points ($N_{cultures} \times (N_{recordings} \times N_{features})$). The training data for the classification was batchwise transformed into *Z* scores to minimize interbatch variability; test data were transformed using parameters derived only from the training data. The model was implemented using the MATLAB function *fitcensemble and templateTree* learners. Accuracy values were obtained using a leave-one-out CV for network predictions (Figures 3 and 4) and a 5-fold CV for single-cell predictions (Figure 5). Hyperparameter optimization was performed on the training set using a 5-fold nested CV and 100 iterations of Bayesian optimization (Table S8). RF was selected for the analysis because it provides the option to infer predictor importance values (MATLAB function: *oobPermutedPredictorImportance*). The implementations of the clustering and the age regression analysis are detailed in the supplemental experimental procedures.

HTRF assay

The HTRF assay (6FNSYPEG, Cisbio Bioassays, Codolet, France) was performed according to the manufacturer's instructions; a detailed protocol is provided in the supplemental experimental procedures.

SUPPLEMENTAL INFORMATION

Supplemental information can be found online at https://doi.org/10.1016/j.stemcr.2023.12.008.

ACKNOWLEDGMENTS

This work was supported by the European Research Council Advanced Grant 694829 "neuroXscales" and the corresponding proof-of-concept grant 875609 "HD-Neu-Screen". Additionally, the study received funds by the two Cantons of Basel through a Personalized Medicine project (PMB-01-18) granted by ETH Zurich, the Innosuisse Project 25933.2 PFLS-LS, the Swiss National Science Foundation under contract 205320_188910/1, and a Swiss Data Science Center project grant (C18-10). Figure 1 was created using BioRender.com. We thank Zahra Ehsaei and Hiap Chon How for their advice on the experimental procedures.

AUTHOR CONTRIBUTIONS

Conceptualization: P.H., M.S., A.H., M.F., and S.R. Methodology: P.H., M.S., and D.R. Investigation: P.H., G.P., M.S., N.A., M.F., and S.R. Software: P.H. and M.S. Formal analysis: P.H. Writing – original draft: P.H. Writing – review & editing: P.H., M.S., D.R., A.H., K.B., C.D., T.K., R.J., and V.T. Funding acquisition: M.S., A.H., K.B., and V.T. Resources: A.H., V.T., and R.J. Supervision: M.S., A.H., V.T., and K.B. Project administration: M.S. and A.H. Funding acquisition: M.S., A.H., V.T., and K.B.

DECLARATION OF INTERESTS

M.F. is a cofounder of MaxWell Biosystems AG, which commercializes HD-MEA technology. The other authors declare no competing interests.

Received: June 17, 2022
Revised: December 18, 2023
Accepted: December 20, 2023
Published: January 25, 2024

REFERENCES

Abbott, J., Ye, T., Krenek, K., Gertner, R.S., Ban, S., Kim, Y., Qin, L., Wu, W., Park, H., and Ham, D. (2020). A nanoelectrode array for obtaining intracellular recordings from thousands of connected neurons. Nat. Biomed. Eng. *4*, 232–241.

Bakkum, D.J., Radivojevic, M., Frey, U., Franke, F., Hierlemann, A., and Takahashi, H. (2014). Parameters for burst detection. Front. Comput. Neurosci. *7*, 193.

Buccino, A.P., Hurwitz, C.L., Garcia, S., Magland, J., Siegle, J.H., Hurwitz, R., and Hennig, M.H. (2020). SpikeInterface, a unified framework for spike sorting. Elife *9*, e61834.

Cutts, C.S., and Eglen, S.J. (2014). Detecting Pairwise Correlations in Spike Trains: An Objective Comparison of Methods and Application to the Study of Retinal Waves. J. Neurosci. *34*, 14288–14303.

Dagra, A., Miller, D.R., Lin, M., Gopinath, A., Shaerzadeh, F., Harris, S., Sorrentino, Z.A., Støier, J.F., Velasco, S., Azar, J., et al. (2021). α-Synuclein-induced dysregulation of neuronal activity contributes to murine dopamine neuron vulnerability. NPJ Parkinsons Dis. *7*, 76.

Dolmetsch, R., and Geschwind, D.H. (2011). The Human Brain in a Dish: The Promise of iPSC-Derived Neurons. Cell *145*, 831–834.

English, D.F., McKenzie, S., Evans, T., Kim, K., Yoon, E., and Buzsáki, G. (2017). Pyramidal Cell-Interneuron Circuit Architecture and Dynamics in Hippocampal Networks. Neuron *96*, 505–520.e7.

Fedele, S., Collo, G., Behr, K., Bischofberger, J., Müller, S., Kunath, T., Christensen, K., Gündner, A.L., Graf, M., Jagasia, R., and Taylor, V. (2017). Expansion of human midbrain floor plate progenitors from induced pluripotent stem cells increases dopaminergic neuron differentiation potential. Sci. Rep. *7*, 6036.

Feigin, V.L., Vos, T., Nichols, E., Owolabi, M.O., Carroll, W.M., Dichgans, M., Deuschl, G., Parmar, P., Brainin, M., and Murray, C. (2020). The global burden of neurological disorders: translating evidence into policy. Lancet Neurol. *19*, 255–265.

Fields, C.R., Bengoa-Vergniory, N., and Wade-Martins, R. (2019). Targeting Alpha-Synuclein as a Therapy for Parkinson's Disease. Front. Mol. Neurosci. *12*, 299.

Flagmeier, P., Meisl, G., Vendruscolo, M., Knowles, T.P.J., Dobson, C.M., Buell, A.K., and Galvagnion, C. (2016). Mutations associated with familial Parkinson's disease alter the initiation and amplification steps of α-synuclein aggregation. Proc. Natl. Acad. Sci. USA *113*, 10328–10333.

Fulcher, B.D., and Jones, N.S. (2017). hctsa: A Computational Framework for Automated Time-Series Phenotyping Using Massive Feature Extraction. Cell Syst. *5*, 527–531.e3.

Kriks, S., Shim, J.-W., Piao, J., Ganat, Y.M., Wakeman, D.R., Xie, Z., Carrillo-Reid, L., Auyeung, G., Antonacci, C., Buch, A., et al. (2011). Dopamine neurons derived from human ES cells efficiently engraft in animal models of Parkinson's disease. Nature *480*, 547–551.

Lee, E.K., Balasubramanian, H., Tsolias, A., Anakwe, S.U., Medalla, M., Shenoy, K.V., and Chandrasekaran, C. (2021). Non-linear dimensionality reduction on extracellular waveforms reveals cell type diversity in premotor cortex. Elife *10*, e67490.

Lubba, C.H., Sethi, S.S., Knaute, P., Schultz, S.R., Fulcher, B.D., and Jones, N.S. (2019). catch22: CAnonical Time-series CHaracteristics. Data Min. Knowl. Discov. *33*, 1821–1852.

Mahmud, M., and Vassanelli, S. (2019). Open-Source Tools for Processing and Analysis of In Vitro Extracellular Neuronal Signals. In In Vitro Neuronal Networks: From Culturing Methods to Neuro-Technological Applications, M. Chiappalone, V. Pasquale, and M. Frega, eds. (Springer International Publishing), pp. 233–250.

Müller, J., Ballini, M., Livi, P., Chen, Y., Radivojevic, M., Shadmani, A., Viswam, V., Jones, I.L., Fiscella, M., Diggelmann, R., et al. (2015). High-resolution CMOS MEA platform to study neurons at subcellular, cellular, and network levels. Lab Chip *15*, 2767–2780.

Petersen, P.C., Siegle, J.H., Steinmetz, N.A., Mahallati, S., and Buzsáki, G. (2021). CellExplorer: A framework for visualizing and characterizing single neurons. Neuron *109*, 3594–3608.e2.

Polymeropoulos, M.H., Lavedan, C., Leroy, E., Ide, S.E., Dehejia, A., Dutra, A., Pike, B., Root, H., Rubenstein, J., Boyer, R., et al. (1997). Mutation in the alpha-synuclein gene identified in families with Parkinson's disease. Science *276*, 2045–2047.

Ronchi, S., Buccino, A.P., Prack, G., Kumar, S.S., Schröter, M., Fiscella, M., and Hierlemann, A. (2021). Electrophysiological Phenotype Characterization of Human iPSC-Derived Neuronal Cell Lines by Means of High-Density Microelectrode Arrays. Adv. Biology *5*, 2000223.

Ryan, S.D., Dolatabadi, N., Chan, S.F., Zhang, X., Akhtar, M.W., Parker, J., Soldner, F., Sunico, C.R., Nagar, S., Talantova, M., et al. (2013). Isogenic human iPSC Parkinson's model shows nitrosative stress-induced dysfunction in MEF2-PGC1α transcription. Cell *155*, 1351–1364.

Scott, D., and Roy, S. (2012). α-Synuclein inhibits intersynaptic vesicle mobility and maintains recycling-pool homeostasis. J. Neurosci. *32*, 10129–10135.

Stringer, C., Pachitariu, M., Steinmetz, N., Reddy, C.B., Carandini, M., and Harris, K.D. (2019). Spontaneous behaviors drive multidimensional, brainwide activity. Science *364*, 255.

Sun, J., Wang, L., Bao, H., Premi, S., Das, U., Chapman, E.R., and Roy, S. (2019). Functional cooperation of α-synuclein and VAMP2 in synaptic vesicle recycling. Proc. Natl. Acad. Sci. USA *116*, 11113–11115.

Trainito, C., von Nicolai, C., Miller, E.K., and Siegel, M. (2019). Extracellular Spike Waveform Dissociates Four Functionally Distinct Cell Classes in Primate Cortex. Curr. Biol. *29*, 2973–2982.e5.

Unakafova, V.A., and Gail, A. (2019). Comparing Open-Source Toolboxes for Processing and Analysis of Spike and Local Field Potentials Data. Front. Neuroinform. *13*, 57.

Volpato, V., Smith, J., Sandor, C., Ried, J.S., Baud, A., Handel, A., Newey, S.E., Wessely, F., Attar, M., Whiteley, E., et al. (2018). Reproducibility of Molecular Phenotypes after Long-Term Differentiation to Human iPSC-Derived Neurons: A Multi-Site Omics Study. Stem Cell Rep. *11*, 897–911.

Wagenaar, D.A., Pine, J., and Potter, S.M. (2006). An extremely rich repertoire of bursting patterns during the development of cortical cultures. BMC Neurosci. *7*, 11.

Wang, L., Das, U., Scott, D.A., Tang, Y., McLean, P.J., and Roy, S. (2014). α-synuclein multimers cluster synaptic vesicles and attenuate recycling. Curr. Biol. *24*, 2319–2326.

Xu, J., Wu, X.-S., Sheng, J., Zhang, Z., Yue, H.-Y., Sun, L., Sgobio, C., Lin, X., Peng, S., Jin, Y., et al. (2016). α-Synuclein Mutation Inhibits Endocytosis at Mammalian Central Nerve Terminals. J. Neurosci. *36*, 4408–4414.

Zambon, F., Cherubini, M., Fernandes, H.J.R., Lang, C., Ryan, B.J., Volpato, V., Bengoa-Vergniory, N., Vingill, S., Attar, M., Booth, H.D.E., et al. (2019). Cellular α-synuclein pathology is associated with bioenergetic dysfunction in Parkinson's iPSC-derived dopamine neurons. Hum. Mol. Genet. *28*, 2001–2013.

Stem Cell Reports, Volume *19*

Supplemental Information

DeePhys: A machine learning–assisted platform for electrophysiological phenotyping of human neuronal networks

Philipp Hornauer, Gustavo Prack, Nadia Anastasi, Silvia Ronchi, Taehoon Kim, Christian Donner, Michele Fiscella, Karsten Borgwardt, Verdon Taylor, Ravi Jagasia, Damian Roqueiro, Andreas Hierlemann, and Manuel Schröter

Supplemental items

Supplemental figures

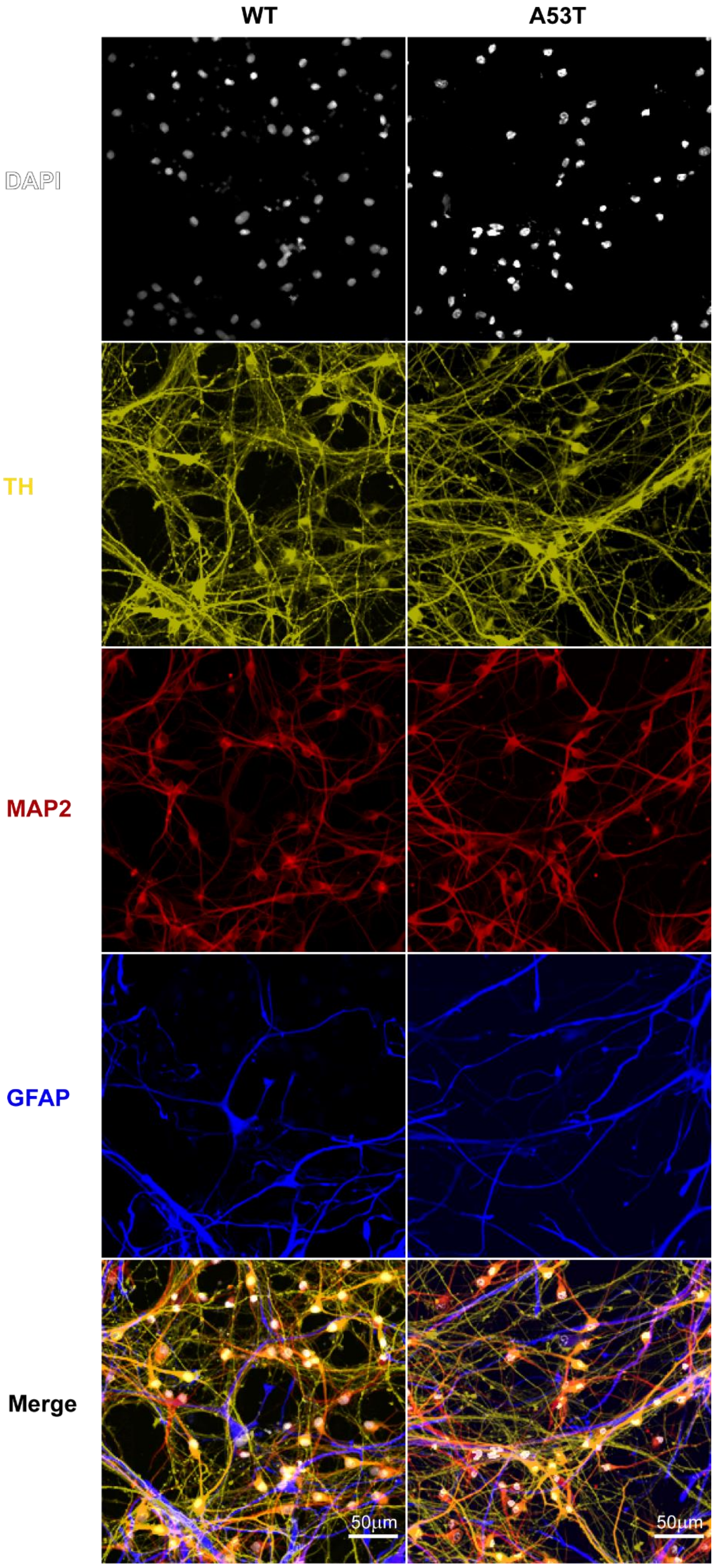

Figure S1. Immuno-cytochemical stainings of WT and A53T co-cultures. DA neuron-astrocyte co-cultures of both genotypes expressed MAP2+ (red) and TH+ (yellow) and formed networks in a similar manner (DIV 21). Stainings with GFAP (blue) indicated a successful integration of astrocytes into the culture. Related to **Figure 3A**.

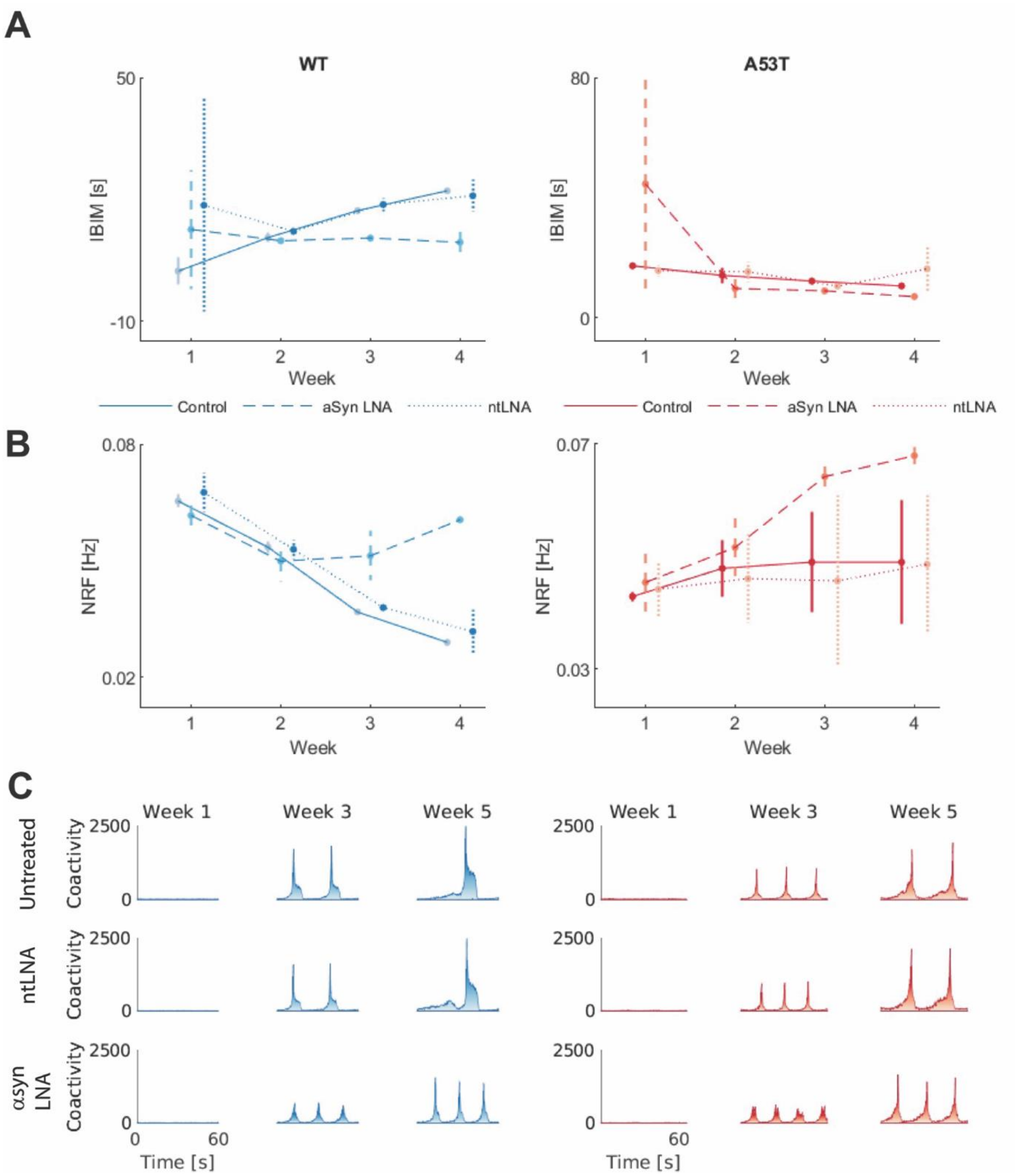

Figure S2. Similar developmental trajectories of untreated and non-targeted LNA-treated cultures. We found similar developmental trajectories in cultures treated with non-targeted LNA (ntLNA; dotted lines) and untreated control cultures (solid lines). Panels **(A)** and **(B)** underline this similarity and show two of the most distinctive features for the two genotypes - the mean interburst interval (IBIM; in panel **A**) and the network regularity frequency (NRF, in panel **B**). The anti-α-synuclein LNA (asyn LNA; dashed lines) treatment, however, had a clear effect on spontaneous activity (e.g., longer interburst intervals). The network coactivity plots in panel **(C)** further underscore this observation: While burst shapes and time intervals were very similar in untreated and ntLNA-treated cultures, αsyn LNA-treated cultures displayed shorter bursts with, on average, smaller amplitudes. Related to **Figure 4**.

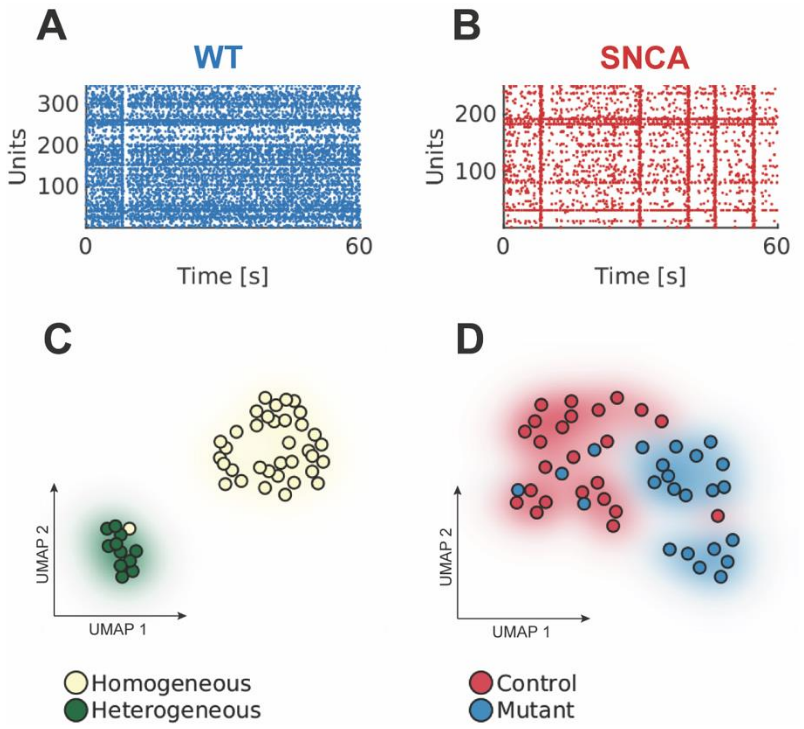

Figure S3. Robust differences between cell lines and cultures with different cellular compositions.

Representative spike raster plots of a heterogeneous midbrain DA WT/control culture **(A)** and a heterogeneous SNCA/mutant culture **(B)**. **(C)** Clustering analysis allowed for a clear separation into homogeneous and heterogeneous midbrain DA cultures (N=36 heterogeneous cultures and N=11 homogeneous cultures). **(D)** Control and mutant lines, here pooled across heterogeneous and homogenous cultures, can also be successfully separated in different clusters (N=24 control and N=23 mutant networks/cultures; the input data was normalized for homogeneous and heterogeneous cultures separately for this analysis). Related to **Figure 5**.

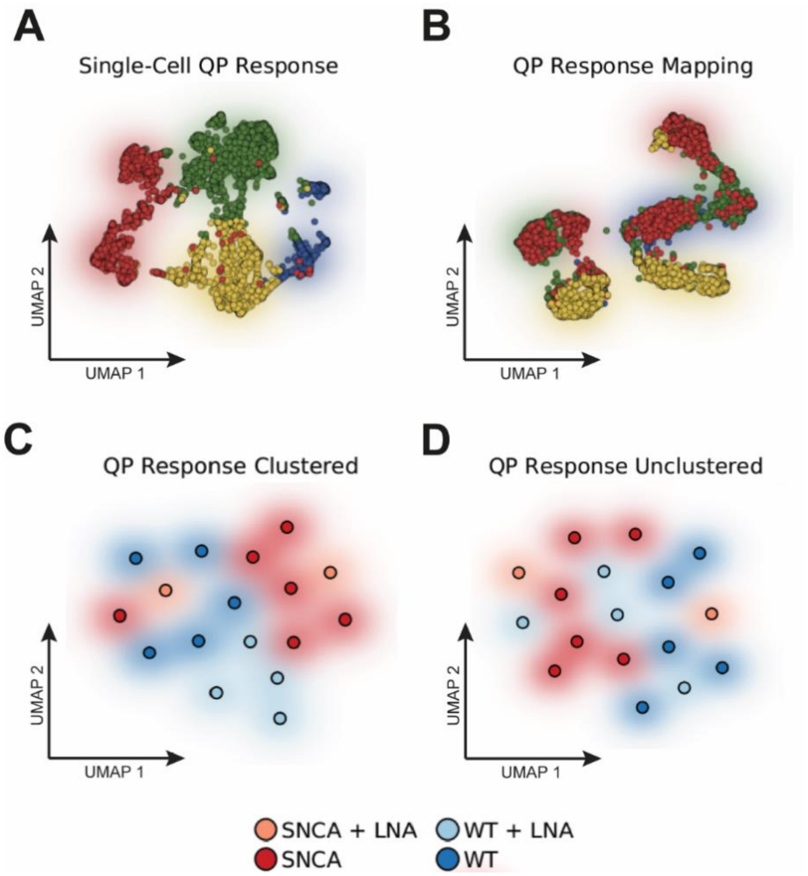

Figure S4. Integration of Quinpirole response modes facilitates phenotyping of heterogeneous cell lines.

(A) Results of a UMAP embedding performed on the single-cell responses after application of the selective D_2 and D_3 receptor agonist Quinpirole (QP). Each dot indicates one cell/response obtained from heterogeneous DA midbrain cultures (in total N=4932 units). Following the UMAP reduction, Louvain clustering was performed: each color (red, green, blue, and yellow) indicates a separate response cluster. **(B)** UMAP embedding based on the baseline activity; colors according to the clusters inferred in **(A)**. **(C)** UMAP embedding of the QP response after inferring single-cell features for each of the single-cell clusters from **(A)** separately (N=17 cultures). **(D)** UMAP embedding of the same QP response without using single-cell clustering, i.e., single-cell features were averaged across the whole network. Related to **Figure 5E-F**.

Supplemental tables

Table S1. Full feature list inferred by *DeePhys*.

**Feature name**	**Feature description**
**Single-cell features**	**Inferred from individual spike-sorted units**
**Waveform features**	**Features inferred from the action potential waveform shape**
AUCP1	Calculates the area under the curve of the action potential waveform peak 1
AUCP2	Calculates the area under the curve of the action potential waveform peak 2
AUCT	Infers the area under the curve of the action potential waveform trough
RISE	Calculates the slope from the action potential waveform trough to peak 2
DECAY	Calculates the slope from the action potential waveform peak 2 back to baseline
ASYM	Calculates the waveform asymmetry, i.e., the ratio of peak 2 and peak 1
T2PR	Calculates the trough-to-peak 2 ratio of the action potential waveform
T2PD	Calculates the trough-to-peak 2 delay of the action potential waveform
**Spike-time features**	**Features inferred from the spike-times of individual units**
MIS	Calculates the mean interspike interval of a spike train
VIS	Calculates the variance of the interspike interval of a spike train
CVI	Calculates the coefficient of variation of the interspike interval
PAF	Infers the partial autocorrelation function of a spike train
**Network features**	**Features inferred across the neuronal network**
**Burst features**	**Inferred from the network burst activity**
MIB	Infers the mean interburst interval, i.e., the interval times between bursts
VIB	Estimates the variance of the interburst intervals
MBD	Calculates the mean burst duration, i.e., the time from the start to the end of bursts
VBD	Measures the variance of the burst durations
MRT	Calculates the mean burst rise time, i.e., the time from 10% to 90% of the maximum coactivity (peak) of a burst
MFT	Calculates the mean burst fall time, i.e., the time from 90% to 10% of the maximum coactivity (peak) of a burst
MRV	Measures the mean burst rise velocity, i.e., the slope from 10% to 90% of the maximum coactivity (peak) of a burst
MFV	Calculates the mean fall velocity, i.e., the slope from 90% to 10% of the maximum coactivity (peak) of a burst
INTRABF	Gives the intraburst firing rate
INTERBF	Infers the interburst firing rate
**Graph features**	**Features inferred from functional connectivity**
DENS	Estimates the graph density, i.e., a measure of how many edges are present compared to the total number of possible edges
RE	Calculates the Rent exponent, i.e., a parameter indicating the scaling relationship between the size of a network and its average degree
ASRT	Estimates the assortativity, i.e., the degree to which nodes tend to be connected to nodes with similar degrees
GE	Calculates the global efficiency; the average inverse shortest path length in a network, it measures how efficiently information can flow in a graph

**Time-series features**	**Inferred from the binned activity of individual units or the whole network**
RF	Calculates the regularity frequency, i.e., the peak frequency of the Fourier power spectrum of a signal
RM	Calculates the regularity magnitude as the magnitude of the RF
RFIT	Estimates the regularity fit, i.e., the exponential fit of consecutive peaks in the Fourier power spectrum
EAF	Calculates the first 1 / e (Euler's number) crossing of the autocorrelation function
AMI	Estimates the auto-mutual information, i.e., the mutual information between a signal and its time-delayed version
SFR	Calculates the proportion of slower timescale fluctuations that scale with linearly rescaled range fits
SFD	Calculates the proportion of slower timescale fluctuations that scale with the Detrended Fluctuation Analysis
LPF	Estimates the total power in the lowest fifth of the frequencies in the Fourier power spectrum
MEF	Infers the mean error from a rolling 3-sample mean forecasting
PAM	Measures the longest period of consecutive values above the mean
EFD	Returns the exponential fit on the sequence of successive Euclidean distances between points in a 2D time-delay embedding space
CCD	Measures the change in correlation length, i.e., the distance over which the time series is correlated with itself, after iterative differencing
TCT	Calculates the trace of covariance of the transition matrix, i.e., the transition probabilities between different states in the time series
SES	Infers the Shannon entropy of two successive local motifs
CFS	Calculates the centroid of the Fourier power spectrum
PMW	Calculates the periodicity measure of [1]
MD5	Calculates the mode of *z*-scored distribution (5-bin histogram)
MD10	Calculates the mode of *z*-scored distribution (10-bin histogram)
FMA	Infers the first minimum of the autocorrelation function
FMI	Gives the first minimum of the automutual information function
TRS	Measures the time-reversal asymmetry, i.e., it quantifies whether the time series behaves differently when its direction is reversed in time
TEA	Gives the average of time intervals between successive extreme events above the mean
TEB	Gives the average of time intervals between successive extreme events below the mean
PDE	Calculates the proportion of successive differences exceeding 0.04 SD [2]
LSD	Infers the longest period of successive incremental decreases

Descriptions of the catch22 time series features (indicated in italics) were adapted from the original papers, which also contain a more detailed explanation [3, 4].

Table S2. List of main functions of *DeePhys*.

Module/Function	Description
**Preprocessing**	
generate_sorting_path_list	Generates a list of paths containing the sorting results (only works if all spike sortings follow the same path logic)
remove_low_unit_recording	Removes the MEArecording objects with too few units after QC
split_sortings	Splits sorting by time and generates new sorting files (useful for analyzing concatenated recordings)
recording_array_from_single_files	Loads individual MEArecording objects into one array
**Feature extraction and Quality control**	
generate_MEArecordings_from_sorting_list	Generates MEArecording objects from a spike sorting list
Class Unit	Instantiates an object that contains information about a single unit and a reference to its corresponding MEArecording
.inferActivityFeatures	Calculates activity features of individual units
Class MEArecording	Instantiates an object that contains all metadata and network feature values
.returnDefaultParams	Returns a structure containing the default parameter values
.performAnalyses	Runs quality control and feature extraction based on the *params* structure provided
.aggregateSingleCellFeatures	Returns a table containing the specified unit features (averages) across the culture
.calculateClusterSingleCellFeatures	Calculates the unit features for each single-cell cluster
.concatenateClusteredFeatures	Returns a table containing the unit features averages across single-cell clusters
.getRecordingFeatures	Returns a table containing the features representative of the whole culture (unit and network features)
.getUnitFeatures	Returns a table containing the unit features for each individual unit
.PlotNetworkScatter	Plots a scatter plot of the network activity
.PlotNetworkScatterHistogram	Same as PlotNetworkScatter, but with a corresponding histogram
.PlotBurstCheck	Plots network coactivity and burst starts and ends as inferred by the burst detection algorithm
.PlotCCG	Plots the crosscorrelogram between two units
.PlotCommunity	Plots connectivity matrix of an inferred graph after maximizing the modularity
.PlotUnitClusterActivity	Plots scatter plots sorted by unit cluster IDs
**Feature integration and phenotyping**	
Class RecordingGroup	Instantiates an object that contains analyses of a group of MEArecordings
.runMLM	Runs linear mixed-effects models to assess differences between conditions statistically
.aggregateSparseFeatureTable	Returns a table containing the features of cultures across development (allows missing values)
.aggregateCultureFeatureTables	Returns a table containing the features of cultures across development
.prepareInputMatrix	Returns input table for machine learning methods

.reduceDimensionality	Performs the selected dimensionality reduction method on the single-cell or network level
.predictAge	Performs random forest regression to predict the age of a culture
.classifyByFeatureGroups	Performs classification between conditions based on the selected feature groups
.assessClassifier	Assesses the performance of the classifier
.classifyByFeatureGroupsAndGroupingVar	Trains classifiers for each feature group and each condition individually
.regressionByFeatureGroups	Performs random forest regression to predict any numerical metadata information (e.g., drug concentration)
.combineMetadataIndices	Returns group indices corresponding to the selected metadata information
.returnFeatureNames	Returns names of features that have been extracted
.clusterByFeatures	Cluster cultures or units using the selected clustering algorithm
.calculateClusterPurity	Calculates the cluster purity of the selected clustering result (requires ground truth)
.plot_feature_trajectories	Plots a line plot of feature values of distinct conditions (e.g., cell lines) across development
.plot_feature_heatmap	Plots a heatmap color coding the relative feature values between two conditions
.plot_regression_results	Plots box plot of regression results
**Assessment of (pharmacological) interventions**	
.applyClassifier	Applies a pretrained classifier to a new group of recordings
.assessAppliedClassifier	Assesses the performance of the pretrained classifier on the new test data
**Single-cell analyses**	
.assignUnitClusterIdx	Permanently assigns a cluster ID to a unit (required for subsequent single-cell analyses)
.removeUnitsByCluster	Removes units belonging to a selected cluster
.classifyClusteredUnits	Trains a classifier based on the results of a single-cell clustering
.applyClusteredUnitClassifier	Applies pretrained classifier on other cultures
.plot_true_clusters	Plots single-cell dimensionality reduction colored by the selected metadata information (e.g., cell line)
.plot_cluster_outlines	Plots dimensionality reduction scatter plot and displays cluster outlines
.plot_single_cluster	Plots dimensionality reduction of a single selected cluster
.plot_cluster_waveforms	Plots waveforms of units belonging to the inferred clusters
.plot_cluster_densities	Plots a heatmap of single-cell cluster densities after dimensionality reduction
.plot_cluster_shifts	Plots differences between cluster densities of different conditions (e.g., cell lines)
.plot_cluster_proportions	Plots a cumulative bar plot of the cluster proportions in distinct conditions (e.g., cell lines)
.plot_unit_cluster_features	Plots a line plot of mean feature values of single-cell clusters
.plot_unit_cluster_heatmap	Plots a heatmap coding the relative feature values of clusters

Table S3. Ratio of TH+/MAP2+ neurons.

Genotype	Treatment	TH+/MAP2+ ratio [%]												Mean ± SD
WT	Untreated	36.9	64.1	57.0	33.6	56.6	62.7	36.5	53.4	62.0	40.6	59.0	70.5	49.16 ± 13.94
		34.8	41.3	58.9	21.3	30.7	41.2	30.1	65.9	60.8	33.8	70.1	57.9	
		41.5	70.8	57.8	42.4	64.9	65.9	41.1	51.9	40.7	32.8	45.7	33.7	
	ntLNA	47.8	51.2	35.2	61.2	55.0	42.8	53.9	57.4	36.0	66.7	61.1	49.3	49.42 ± 9.62
		57.8	42.2	58.8	50.0	47.7	43.2	39.5	56.6	59.7	30.3	58.5	63.0	
		32.8	56.8	52.7	37.6	58.3	43.3	42.0	52.1	45.1	33.6	45.1	55.0	
	LNA	27.2	59.0	54.1	38.7	62.9	58.5	32.9	50.8	58.0	30.1	56.5	54.9	43.81 ± 13.29
		34.6	49.8	56.7	23.4	34.4	40.6	22.6	41.6	34.1	30.6	68.0	58.8	
		51.4	46.8	43.3	40.0	51.1	51.1	30.7	54.0	56.1	18.5	30.9	25.1	
A53T	Untreated	39.3	27.6	28.0	54.0	27.6	28.0	59.4	40.7	38.2	47.4	50.2	39.8	43.93 ± 11.33
		51.9	44.4	52.3	58.0	69.4	52.9	38.9	30.7	33.8	50.2	37.3	27.3	
		51.4	41.8	26.4	53.3	61.8	38.2	56.6	40.8	38.5	59.1	45.6	40.6	
	ntLNA	59.9	42.6	23.3	50.0	44.2	25.0	45.8	42.9	38.6	48.7	58.2	39.0	42.10 ± 13.91
		54.8	56.2	38.6	41.6	47.2	42.3	33.5	28.3	4.44	35.1	39.3	23.1	
		50.5	34.2	23.1	62.2	58.8	24.2	53.5	57.3	27.5	64.6	59.5	37.4	
	LNA	30.4	16.1	21.6	37.9	19.2	35.4	29.8	27.6	31.7	40.5	29.6	37.0	31.65 ± 8.99
		32.6	25.6	17.6	15.3	24.3	22.0	31.3	28.2	21.7	40.5	33.3	34.7	
		39.1	46.6	30.9	54.1	38.7	31.4	31.3	49.8	36.2	38.5	34.8	24.1	

ANOVA table	SS	DF	MS	F (DFn, DFd)	p value
Interaction	0.046	2	0.023	F (2, 210) = 1.6	p=0.208
Mutation	0.366	1	0.366	F (1, 210) = 25.4	p<0.001
Treatment	0.343	2	0.171	F (2, 210) = 11.9	p<0.001
Residual	3.031	210	0.014		

Tukey-Kramer test	Adjusted p value	Tukey-Kramer test	Adjusted p value
WT:Untreated vs. WT:ntLNA	>0.999	WT:ntLNA vs. A53T:LNA	<0.001
WT:Untreated vs. WT:LNA	0.607	WT:LNA7 vs. A53T:Untreated	>0.999
WT:Untreated vs. A53T:Untreated	0.643	WT:LNA vs. A53T:ntLNA	0.999
WT:Untreated vs. A53T:ntLNA	0.184	WT:LNA vs. A53T:LNA	<0.001
WT:Untreated vs. A53T:LNA	<0.001	A53T:Untreated vs. A53T:ntLNA	0.999
WT:ntLNA vs. WT:LNA	0.530	A53T:Untreated vs. A53T:LNA	<0.001
WT:ntLNA vs. A53T:Untreated	0.566	A53T:ntLNA vs. A53T:LNA	0.004
WT:ntLNA vs. A53T:ntLNA	0.146		

Ratio of $TH^+/MAP2^+$ cells per imaged field. For each condition, six cultures were analyzed by quantifying the intensity of six fields, each consisting of 3x3 images. Related to **Figure 2A**.

Table S4. Quantification of total α-synuclein levels by Homogeneous Time Resolved Fluorescence (HTRF) assay.

Genotype	Treatment	HTRF [intensity]									Mean ± SD
WT	Untreated	63.8	64.6	65.7	70.2	70.1	70.4	64.2	65.3	65.5	66.6 ± 2.8
	ntLNA	74.3	74.9	73.4	70.7	71.1	70.3	71.1	71.2	71.2	72.0 ± 1.7
	LNA	5.4	5.6	5.5	5.5	5.6	5.5	5.2	5.3	5.1	5.4 ± 0.2
A53T	Untreated	74.3	72.9	72.6	63.9	66	60.8	63.7	63.8	64.5	66.9 ± 4.9
	ntLNA	71.3	71.9	72.5	67.9	67.2	68.7	39.8*	39.5*	40.0*	69.9 ± 2.3
	LNA	5.5	5.9	5.8	6.5	6.3	6.4	6.5	6.7	6.4	6.2 ± 0.4

ANOVA table	SS	DF	MS	F (DFn, DFd)	p value
Interaction	6.2	2	3.1	F (2, 11) = 0.4	p=0.705
Mutation	0.5	1	0.5	F (1, 11) = 0.05	p=0.820
Treatment	15282.7	2	7641.3	F (2, 11) = 885.3	p<0.001
Residual	94.9	11	8.6		

Tukey-Kramer test	Adjusted p value	Tukey-Kramer test	Adjusted p value
WT:Untreated vs. WT:ntLNA	0.293	WT:ntLNA vs. A53T:LNA	<0.001
WT:Untreated vs. WT:LNA	<0.001	WT:LNA7 vs. A53T:Untreated	<0.001
WT:Untreated vs. A53T:Untreated	>0.999	WT:LNA vs. A53T:ntLNA	<0.001
WT:Untreated vs. A53T:ntLNA	0.819	WT:LNA vs. A53T:LNA	0.999
WT:Untreated vs. A53T:LNA	<0.001	A53T:Untreated vs. A53T:ntLNA	0.868
WT:ntLNA vs. WT:LNA	<0.001	A53T:Untreated vs. A53T:LNA	<0.001
WT:ntLNA vs. A53T:Untreated	0.345	A53T:ntLNA vs. A53T:LNA	<0.001
WT:ntLNA vs. A53T:ntLNA	0.964		

Quantification of total α-synuclein levels by a Homogeneous Time Resolved Fluorescence (HTRF) assay. For each condition, three biological replicates (different cultures), each with three technical replicates were analyzed. The samples indicated with asterisks were considered outliers and excluded from the analysis. Related to **Figure 4B**.

Table S5. Quantification of somatic α-synuclein levels by immunocytochemical staining analysis.

Genotype	Treatment	Somatic α-synuclein levels [intensity]									Mean ± SD
WT	Untreated	817.6	674.8	683.3	744.5	719.4	701.7	665	698.9	667.7	721.4 ± 57.6
		802	726.2	754	660.3	621.8	691.5	808.7	753.2	794	
	ntLNA	791.9	712.4	824.3	584.2	648.6	615.6	635.1	734.2	683.4	723.1 ± 91.3
		731.6	713.4	702.7	600.6	637.7	559.5	821	830.7	792.4	
	LNA	271	258.1	273.2	256	296.3	272.9	264.2	276	256.6	272.4 ± 13.9
		284.6	299.8	265.9	270.5	273.2	249.6	269.6	272	293	
A53T	Untreated	781.4	786.2	720	690.3	806.5	859.1	689.8	733.6	690.9	721.5 ± 75.6
		791.9	712.4	824.3	584.2	648.6	615.6	635.1	734.2	683.4	
	ntLNA	878.2	869.9	892.5	872.8	801.6	793.7	734.9	767.6	684	775.1 ± 66.9
		733.4	764.7	738.6	701.3	675.2	721.6	793.7	756.1	771.1	
	LNA	291.5	252.7	257.6	273.9	298.6	283.8	269.3	288.6	287.4	275.9 ± 12.6
		274.4	281.3	282	271.3	258.3	274.9	267.9	265.9	286.1	

ANOVA table	SS	DF	MS	F (DFn, DFd)	p value
Interaction	15148.8	2	7574.3	F (2, 102) = 2.0	p=0.134
Mutation	9292.6	1	9292.6	F (1, 102) = 2.5	p=0.116
Treatment	5117295.0	2	2558647.5	F (2, 102) = 691.9	p<0.001
Residual	377216.7	102	3698.2		

Tukey-Kramer test	Adjusted p value	Tukey-Kramer test	Adjusted p value
WT:Untreated vs. WT:ntLNA	>0.999	WT:ntLNA vs. A53T:LNA	<0.001
WT:Untreated vs. WT:LNA	<0.001	WT:LNA7 vs. A53T:Untreated	<0.001
WT:Untreated vs. A53T:Untreated	>0.999	WT:LNA vs. A53T:ntLNA	<0.001
WT:Untreated vs. A53T:ntLNA	0.132	WT:LNA vs. A53T:LNA	>0.999
WT:Untreated vs. A53T:LNA	<0.001	A53T:Untreated vs. A53T:ntLNA	0.134
WT:ntLNA vs. WT:LNA	<0.001	A53T:Untreated vs. A53T:LNA	<0.001
WT:ntLNA vs. A53T:Untreated	>0.999	A53T:ntLNA vs. A53T:LNA	<0.001
WT:ntLNA vs. A53T:ntLNA	0.163		

Quantification of somatic α-synuclein levels by immunocytochemical stainings. For each condition, three cultures were analyzed by quantifying the intensity of six fields, each consisting of 3x3 images. Related to **Figure 4**.

Table S6. Quantification of somatic phosphorylated α-synuclein levels by immunocytochemical stainings.

Genotype	Treatment	Somatic phosphorylated α-synuclein levels [intensity]									Mean ± SD
WT	Untreated	281.3	197.1	210.3	258.5	231.5	216.1	197	207.9	201.7	225 ± 22.9
		218.6	227.7	217.6	225.7	215.6	208.2	244.9	254.4	238.1	
	ntLNA	215.9	234.7	239.9	211.5	233.8	284	213	213.3	246.3	226.9 ±21.4
		211.5	203.5	248.1	201.5	196	230.3	238.9	238.9	222.4	
	LNA	156.1	157.1	155.8	161.4	160.6	160.9	160.3	157.8	157.1	159.6 ± 2.4
		163.7	161.1	160.7	160.6	157.2	157.7	161.2	164	159.6	
A53T	Untreated	239.6	247.8	265.4	233.2	245.9	290	207.7	207.5	271.3	235.1 ± 24.7
		224.5	211.1	256.9	210.3	201.9	244	227.7	219.7	226.6	
	ntLNA	234.4	238.9	260.6	249.5	226.2	293.2	249	216	275.6	246.8 ± 20.4
		238	232.4	245.3	227.2	227.5	236.3	253.5	275.5	264.1	
	LNA	159.1	175	160.4	163.2	171.1	166.2	163.9	167.3	163.8	165.2 ± 3.7
		162.9	165.7	166.9	162.6	164.5	165.4	167.1	162.6	166.5	

ANOVA table	SS	DF	MS	F (DFn, DFd)	p value
Interaction	976.7	2	488.3	F (2, 102) = 1.4	p=0.241
Mutation	3791.4	1	3791.4	F (1, 102) = 11.2	p=0.001
Treatment	121986.8	2	60993.4	F (2, 102) = 180.1	p<0.001
Residual	34547.8	102	338.7		

Tukey-Kramer test	Adjusted p value	Tukey-Kramer test	Adjusted p value
WT:Untreated vs. WT:ntLNA	0.999	WT:ntLNA vs. A53T:LNA	<0.001
WT:Untreated vs. WT:LNA	<0.001	WT:LNA7 vs. A53T:Untreated	<0.001
WT:Untreated vs. A53T:Untreated	0.587	WT:LNA vs. A53T:ntLNA	<0.001
WT:Untreated vs. A53T:ntLNA	0.008	WT:LNA vs. A53T:LNA	0.941
WT:Untreated vs. A53T:LNA	<0.001	A53T:Untreated vs. A53T:ntLNA	0.396
WT:ntLNA vs. WT:LNA	<0.001	A53T:Untreated vs. A53T:LNA	<0.001
WT:ntLNA vs. A53T:Untreated	0.764	A53T:ntLNA vs. A53T:LNA	<0.001
WT:ntLNA vs. A53T:ntLNA	0.018		

Quantification of somatic phosphorylated α-synuclein levels by immunocytochemical stainings. For each condition, three cultures were analyzed by quantifying the intensity of six fields, each consisting of 3x3 images. Related to **Figure 4**.

Table S7. Spike-sorting parameters for Kilosort 2.5.

detect_threshold	5.5	projection_threshold	[10 4]
preclust_threshold	8	car	True
minFR	0.01	minfr_goodchannels	0.01
nblocks	5	sig	20
freq_min	150	sigmaMask	30
nPCs	3	ntbuff	64
nfilt_factor	4	NT	None
do_correction	False	wave_length	61
keep_good_only	False	skip_kilosort_preprocessing	False
scaleproc	None	save_rez_to_mat	False
delete_tmp_files	True	delete_recording_dat	False
n_jobs	-1	chunk_duration	1s

Table S8. Random Forest hyperparameter optimization parameters.

Hyperparameter	Search range
MinLeafSize	range(1,max(2, floor(NumObservations/2)))
MaxNumSplits	range(1, max(2,NumObservations-1))
NumLearningCycles	range(10, 500)
SplitCriterion	gdi, deviance, twoing
NumVariablesToSample	range(1, max(2,NumPredictors))

Further information about the hyperparameters can be found in the official Matlab documentation.

Table S9. List of primary and secondary antibodies.

**Primary antibody**	**Dilution**	**Catalogue number**
mouse anti-TH	1:500	#MAB318, Sigma-Aldrich
chicken anti-MAP2	1:1000	#CH22103, Neuromics (Edina, MN, USA)
rabbit anti-GFAP	1:500	#Z0334, Agilent (Santa Clara, CA, USA)
rabbit anti-phospho-α-synuclein	1:100	Britschgi lab, Roche (Basel, Switzerland)
rabbit anti-α-synuclein	1:100	#2628, Cell Signaling Technology (Danvers, MS, USA)
**Secondary antibody**		
donkey anti-mouse 488	1:250	#A-21202, ThermoFisher
goat anti-chicken 647	1:500	#A-32933, ThermoFisher
donkey anti-rabbit 568	1:250	#A-A10042, ThermoFisher

Supplemental experimental procedures

High-density microelectrode array recordings

Electrophysiological recordings were obtained using two types of complementary-metal-oxide-semiconductor (CMOS)-based high-density microelectrode arrays (HD-MEA) by MaxWell Biosystems (MaxWell Biosystems, Zurich, Switzerland): the single-well HD-MEA "MaxOne" and the 6-well plate HD-MEA "MaxTwo". The wells of both types of HD-MEAs feature a total of 26'400 electrodes in a 120 x 220 electrode grid with a microelectrode center-to-center spacing of 17.5 µm, an overall sensing area of 3.85 × 2.10 mm^2, and allows for simultaneous recordings from up to 1024 electrodes at a sampling rate of 20 kHz (MaxOne) or 10 kHz (MaxTwo)[5]. Recordings were performed inside an incubator at 36°C and 5% CO_2 using the MaxLab Live recording software (MaxWell Biosystems). Recordings started at day *in vitro* (DIV) 7 and were subsequently performed once a week over the course of 5 weeks. Each recording consisted of an activity scan to determine the electrode selection and a subsequent network recording. The activity scan consisted of 7 sparse electrode configurations (center-to-center spacing: 35 µm, every 2nd electrode), that were recorded 2 minutes each. To capture the dynamics of developing neuronal networks, electrodes displaying the highest firing rate were selected; spontaneous network activity was recorded for 15 minutes.

Cell culture and plating

Cell lines

Homogeneous neuronal cultures: Human iPSC-derived DA neurons carrying a heterozygous A53T mutation (cat. C1112, iCell DopaNeurons A53T), an isogenic control line (cat. C1087, iCell DopaNeurons) and astrocytes (cat. R1092, iCell Astrocytes) were purchased from FUJIFILM Cellular Dynamics International (FCDI, Madison, WI, United States). The A53T cell line was generated through nuclease-mediated single-nucleotide polymorphism alterations of the isogenic control line. Midbrain DA neuron differentiation from iPSCs was based on a protocol from the Lorenz Studer lab[6]. The vendor guarantees a purity of at least 70% for midbrain DA neurons and 95% for astrocytes.

The protocol to plate human neurons on HD-MEAs was previously established by our group[7]. Prior to cell plating, HD-MEAs were sterilized in 70% ethanol for 30 minutes and rinsed 3 times with sterile deionized (DI) water. To enhance cell adhesion, the electrode area was covered with 10 µL of 0.05 mg/mL poly-L-ornithine (PLO) solution (cat. A-004-C, Sigma-Aldrich, Saint Louis, MO, United States) and incubated at 37°C for 2 hours. Next, the PLO solution was aspirated, and the HD-MEA was rinsed 3 times with sterile DI water. Next, we added 10 µL of 80 µg/ml laminin (cat. L2020-1MG, Sigma-Aldrich) in plating medium (see below) directly on the electrode area and incubated the chips at 37°C for 30 minutes. The plating medium consisted of 95 mL of BrainPhys Neuronal Medium (cat. 05790, STEMCELL Technologies, Vancouver, Canada), 2 mL of iCell Neural Supplement B (cat. M1029, FCDI), 1 mL iCell Nervous System Supplement (cat. M1031, FCDI), 1 mL N-2 Supplement (100X, cat. 17502048, Gibco), 100 µL laminin (1 mg/mL, cat. L2020-1MG, Sigma-Aldrich) and 1 mL Penicillin-Streptomycin (100X, cat. 15140122, Gibco). In the meantime, the cryovials containing the DA neurons and astrocytes were thawed in a 37°C water bath for 3 minutes. The cells were then transferred to 50 mL centrifuge tubes, and 8 mL plating medium (at room temperature) were drop-wise added (numbers are indicated for 20 chips). Next, the cell suspensions were centrifuged at 380 g for 5 minutes, and the supernatant was aspirated. Cell pellets were then resuspended in plating medium and combined to achieve a final concentration of 10'000 DA neurons and 2000 astrocytes per µL. Finally, 100'000 DA neurons and 20'000 astrocytes were seeded on each HD-MEA by adding 10 µL of the prepared solution directly to the laminin droplet. Next, chips were incubated for 1 hour, and 1.5 mL of plating medium were carefully added. Chips were equipped with a lid, placed inside a 100 mm petri dish - to facilitate transport and to reduce the risk of contamination - and kept inside an incubator at 37°C and 5% CO_2. Additionally, a 35-mm petri dish, filled with DI water, was placed inside the larger petri dish to counteract evaporation. One day after the plating, we replaced 50% of the medium and resumed the normal media change protocol (one third of the medium was exchanged twice a week). Cultures were allowed to equilibrate for 3 days after the medium change to prevent effects on the recordings.

Heterogeneous neuronal cultures: We used the following human iPSC lines: The mutant line SNCA^{tri} (SFC831-03-01, https://cells.ebisc.org/STBCi024-A/, [8]) purchased from the European Bank for

induced pluripotent Stem Cells (EBiSC), and a control iPSC line (SFC840-03-01, https://hpscreg.eu/cell-line/STBCi026-B, [9]) purchased from EBiSC. To derive DA neurons from human iPSCs, an mFPP-based DA neuron differentiation protocol was used [6, 10]. Briefly, human iPSCs were disaggregated using Accutase (cat. AT104, Innovative Cell Technologies Inc., San Diego, CA, United States) for 5-10 minutes, contrifuged at 100 g for 4 minutes and plated on Matrigel-coated (cat. 354277, BD Biosciences, Franklin Lakes, New Jersey, United States) multiwells in the presence of Rock inhibitor (cat. 688000, Sigma-Aldrich) at the density of 200’000 cells/cm^2. DA neuron differentiation was induced when the cells reached a confluent state. In the floor plate induction phase, from day 0 to day 10, 100 nM StemMACS™ LDN193189 (cat.130-103-925, Miltenyi Biotec, Bergisch Gladbach, Germany) and from day 0 to day 4, 10 µM StemMACS™ SB431542 (cat. 130-106-543, Miltenyi Biotec) were added to the medium. From day 1 to day 6, the medium contains 100 ng/mL Human SHH (C24II) (cat. 130-095-727, Miltenyi Biotec), 100 ng/mL Human FGF-8b (cat. 130-095-740, Miltenyi Biotec) and 2µM StemMACS™ Purmorphamine (cat. 130-104-465, Miltenyi Biotec). 3 µM StemMACS™ CHIR99021 (cat. 130-103-926, Miltenyi Biotec) was added from day 3 until day 11. Floor plate induction medium (see below for details) was used from day 0 till day 10. On day 5 of differentiation, the floor plate induction medium was gradually shifted to the floor plate expansion medium (see below for details) to contain 25%, 50% and 75%.

Prior to cell plating, HD-MEAs were sterilized in 70% ethanol for 30 minutes and rinsed 3 times with sterile deionized (DI) water. To enhance cell adhesion, the electrode area was covered with 10 µL of 0.05 mg/mL poly-L-ornithine (PLO) solution (cat. A-004-C, Sigma-Aldrich) and incubated at 37°C overnight. Next, the PLO solution was aspirated, and the HD-MEA was rinsed 3 times with sterile DI water. We then added 10 µL of 20 µg/ml laminin (cat. 11 243 217 001, Sigma-Aldrich) directly on the electrode area and incubated the chips at 37°C for 1 hour. Human iPSCs were disaggregated using Accutase for 5-10 minutes, centrifuged at 100 g for 4 minutes and 120’000 neurons were seeded on the HD-MEA by adding 10 µL of the prepared cell solution. Next, chips were incubated for 1 hour, and 1.2 mL of plating medium were carefully added. From the day of the plating (day 11) onwards, DA neuron progenitors were induced to the DA neuron fate by shifting to DA differentiation medium supplemented with 20 ng/mL Human BDNF (cat. 130-096-286, Miltenyi Biotec), 20 ng/mL Human GDNF (cat. 130-098-449, Miltenyi Biotec), 1 ng/mL Human TGF-β3 (cat.130-094-007, Miltenyi Biotec), 200 µM ascorbic acid (cat. A4034, Sigma-Aldrich), 0.5 mM dibutyryl cAMP (cat. D0627, Sigma-Aldrich) and 10 µM DAPT (cat. 2634, Tocris Bioscience, Minneapolis, Minnesota, United States). Twice a week, 1 mL of DA differentiation medium was replaced.

The floor plate induction medium_contains KnockOut™ DMEM (cat. 10829-018, Gibco) supplemented with 15% KnockOut™ Serum Replacement (cat. 10828010, Gibco), 2 mM GlutaMAX™ Supplement (cat. 35050-038, Gibco), 1X MEM Non-Essential Amino Acids (cat. 11140-035, Gibco), 50 U/mL Penicillin-Streptomycin (PenStrep) (cat. 15140122, Gibco) and 55 µM 2-Mercaptoethanol (cat. 21985-023, Gibco).

The floor plate expansion medium_contains DMEM/F12 with HEPES buffer (cat. 11330-032, Gibco) supplemented with 1X N-2 Supplement (cat. 17502-048, Gibco), 2 mM GlutaMAX™ Supplement, 0.0016 g/mL D-Glucose and 50 U/mL PenStrep.

The DA neuron differentiation medium contains Neurobasal medium (cat. 21103-049, Gibco) supplemented with 1X B-27™ Supplement, serum free (cat. 17504-044, Gibco), 2 mM GlutaMAX™ Supplement, and 50 U/mL PenStrep.

HD-MEA datasets

Inclusion criteria: In the present study, only cultures with recordings from all recording time points were included in the classification analysis. HD-MEA recordings were excluded, if a culture showed signs of detachment, or if the HD-MEA displayed severe malfunctions.

Genotype comparison: The dataset for the genotype comparison (**Figure 3**) consisted of N=18 WT and N=19 A53T cultures, pooled across two batches with identical cell culture and recording protocols. No statistical method was used to predetermine the sample size. For the classification analysis, the dataset consisted of N=14 WT and N=15 A53T cultures. A fraction of the dataset used in this study has been generated for a previous study by our group[7], which, however, did not include a more systematic analysis of waveform/network features of spike-sorted units.

LNA treatment: The dataset of the LNA treatment (**Figure 4**) consisted of N=10 WT and N=10 A53T cultures for the statistical analysis, and N=8 WT and N=8 A53T cultures for the classification analysis.

Single-cell analysis: The dataset for the single-cell analysis (**Figure 5**) consisted of a total of 18'174 cells pooled from 31 cultures.

Clustering

Data was batch-wise transformed into z-scores to minimize inter-batch variability, and z-scores were then used to perform Uniform Manifold Approximation and Projection (UMAP) [11]. The clustering procedure differed between networks and single-cell inputs: To calculate the separability of network genotypes in the low dimensionality space, k-means clustering (k=2) was performed on the UMAP embedding. Cluster centroid positions were initialized 100 times using the k-means++ algorithm in Matlab, and the solution with the lowest within-cluster sums of point-to-centroid distances was reported (i.e., here the squared Euclidean distance). Finally, the clustering purity was computed as: $\frac{1}{N}\sum_{i=1}^{k} |c_i \cap t_j|$ where N is the number of data points, k is the number of clusters, c_i is a cluster, and t_j is the classification with the maximum count for cluster c_i. Single-cell clustering was performed as previously described [12] by applying the Louvain community detection algorithm [13] on the UMAP graph obtained from single-cell waveform or the combination of single-cell waveforms and activity features.

Age prediction

The prediction of culture age was performed by training random forest regression models (Matlab function *fitrensemble*) on features from individual time points, and results were obtained using leave-one-out cross-validation. The assessment of the LNA treatment on the development was performed using random forest regression models trained on all untreated cultures of the same genotype.

Feature inference

Single-cell features

Waveform features:

1. *Half width (HLFW)* was defined as the width of the trough at half the trough amplitude value V_U^{trough} of one unit *U*.
2. *Asymmetry (ASYM)* was defined as the ratio of the difference and the sum of the peaks after (V_U^{peak2}) and before (V_U^{peak1}) the trough of unit *U*:
$$ASYM_U = \frac{V_U^{peak2} - V_U^{peak1}}{V_U^{peak2} + V_U^{peak1}}$$
3. *Trough-to-peak ratio (T2PR)* was defined as the absolute value of the ratio of the trough V_U^{trough} and the second peak V_U^{peak2} of unit *U:*
$$T2PR = \left|\frac{V_U^{trough}}{V_U^{peak2}}\right|$$
4. *Trough-to-peak delay (T2PD)* was defined as the time difference between the occurrence of the trough t_U^{trough} and the second peak t_U^{peak2} of one unit *U*:
$$T2PD = t_U^{peak2} - t_U^{trough}$$
5. *Peak area under the curve (AUCP)* was defined as the integral of the waveform WF_u between the zero crossings before (z_U^1) and after (z_U^2) the respective peak V_U^{peak1} or V_U^{peak2} of one unit *U*:
$$AUCP = \int_{z_U^1}^{z_U^2} WF_u$$
6. *Trough area under the curve (AUCT)* was defined as the integral of the waveform WF_u between the zero crossings before (z_U^1) and after (z_U^2) the trough V_U^{trough} of one unit *U*:
$$AUCT = \int_{z_U^1}^{z_U^2} WF_u$$
7. *Rise (RISE)* was defined as the slew rate from V_U^{trough} to V_U^{peak2} (10% to 90%) of one unit *U*:
$$RISE = \frac{V_U^{peak2} - V_U^{trough}}{t_U^{peak2} - t_U^{trough}}$$
8. *Decay (DECAY)* was defined as the slew rate from V_U^{peak2} to the resting potential V_U^{rest} (10% to 90%) of one unit *U*:
$$DECAY = \frac{V_U^{rest} - V_U^{peak2}}{t_U^{rest} - t_U^{peak2}}$$

Spike-time features:

9. *Mean interspike interval (MIS)* was defined as the average time between spiking events $ISI_i = t_{i+1}^{sp} - t_i^{sp}$ of one unit *U* over a defined number of spikes *N*:
$$MIS_U = \frac{1}{N-1}\sum_{i=1}^{N-1} ISI_i$$
10. *Interspike interval variance (VIS)* was defined as the variance of interspike intervals ISI_i of one unit *U* over a defined number *N* of ISIs:
$$VIS_U = \frac{1}{N}\sum_{i=1}^{N}(ISI_i - ISIM)^2$$
11. *Interspike interval coefficient of variation (CVI)* was defined as the ratio of the standard deviation to the mean of the interspike intervals ISI_i of one unit *U*:
$$CVI_U = \frac{\sqrt{ISIV_U}}{ISIM_U}$$
12. *Partial autocorrelation function (PAF)* was defined as the partial autocorrelation of lag 1 for all ISI_t of one unit *U*:

$$PAF = corr(ISI_{t+1}, ISI_t)$$

Network features

Burst features

1. *Mean interburst interval (MIB)* was defined as the average time from the end of one burst to the beginning of the next burst $IBI_i = t_{i+1}^{start} - t_i^{end}$ across all *N* bursts of one recording:

$$MIB = \frac{1}{N-1}\sum_{i=1}^{N-1} IBI_i$$

2. *Interburst interval variance (VIB)* was defined as the variance across all *N* IBIs of one recording:

$$VIB = \frac{1}{N}\sum_{i=1}^{N}(IBI_i - IBIM)^2$$

3. *Mean burst duration (MBD)* was defined as the average time from beginning t_i^{start} to the end t_i^{end} of a burst across all *N* bursts of one recording:

$$MBD = \frac{1}{N}\sum_{i=1}^{N} t_i^{end} - t_i^{start}$$

4. *Burst duration variance (VBD) was* defined as the variance across all *N* BDs of one recording:

$$VBD = \frac{1}{N}\sum_{i=1}^{N}(BD_i - MBD)^2$$

5. *Intra-burst firing rate (INTRABF)* was defined as the number of spikes n_B^{sp} during the total bursting time T_B of one recording:

$$INTRABF = \frac{n_B^{sp}}{T_B}$$

6. *Inter-burst firing rate (INTERBF)* was defined as the number of spikes n_{NB}^{sp} during the total non-bursting time T_{NB} of one recording:

$$INTERBF = \frac{n_{NB}^{sp}}{T_{NB}}$$

7. *Burst rise time (BRT)* was defined as the average time from the beginning t_i^{start} to the peak t_i^{peak} of a burst across all *N* bursts of a recording:

$$BRT = \frac{1}{N}\sum_{i=1}^{N} t_i^{peak} - t_i^{start}$$

8. *Burst rise velocity (BRV)* was defined as the average slew rate from the coactivity at the beginning C_i^{start} to the coactivity at the peak $C_i^{peak}$of a burst (10% to 90%) across all *N* bursts of a recording:

$$BRV = \frac{1}{N}\sum_{i=1}^{N}\frac{C_i^{peak} - C_i^{start}}{t_i^{peak} - t_i^{start}}$$

9. *Burst fall time (BFT)* was defined as the average time from the peak t_i^{peak} to the end $t_i^{end}$of a burst across all *N* bursts of a recording:

$$BFT = \frac{1}{N}\sum_{i=1}^{N} t_i^{end} - t_i^{peak}$$

10. *Burst fall velocity (BFV)* was defined as the average slew rate from the coactivity at the peak C_i^{peak} to the coactivity at the end C_i^{end} of a burst (10% to 90%) across all *N* bursts of a recording:

$$BFV = \frac{1}{N}\sum_{i=1}^{N}\frac{C_i^{end} - C_i^{peak}}{t_i^{end} - t_i^{peak}}$$

Graph features

All graph features were calculated using the Brain Connectivity Toolbox [14].

Time-series features

1. *Regularity frequency (RF)* was defined as the frequency with the highest magnitude of the Fourier-transformed activity (Act)*:*
$$RF = argmax(\hat{f}(Act))$$
2. *Regularity magnitude (RM)* was defined as the magnitude of the peak frequency of the Fourier-transformed activity:
$$RM = max(\hat{f}(Act))$$
3. *Resonance fit (RFIT)* was defined as the exponential decay constant *d* of the fit through log10-transformed magnitudes *Y* of the regularity frequency harmonics *X*. The exponential model to fit is of the form:
$$Y = a \times e^{d \times X}$$

All other time-series features (*catch22*) were calculated using the toolbox published in [3]. Time-series features were calculated for each individual unit and the whole network from the binned activity (bin size: 100 ms).

HTRF assay

The Homogeneous Time Resolved Fluorescence assay (6FNSYPEG, Cisbio Bioassays, Codolet, France) was performed according to the manufacturer's instructions, and fluorescence emission at the acceptor (665nm) and donor wavelength (620 nm) were measured in a microplate reader (PHERAstar FSX, BMG LABTECH, Ortenberg, Germany). Total protein concentration was determined using the Pierce™ BCA Protein Assay Kit (23225, ThermoFisher). The ratios of acceptor and donor emission signals were calculated for each individual well and normalized by the total protein concentration. For each condition, three cultures (N=3) and three technical replicates were measured.

Immunocytochemistry

Cells were fixed using 8% paraformaldehyde solution (15714S, Electron Microscopy Sciences, Hatfield, USA) and blocked for 1 hour at room temperature (RT) in a blocking buffer containing 10% normal donkey serum (017-000-001, Jackson ImmunoResearch, West Grove, USA), 1% bovine serum albumin (BSA) (05482, Sigma-Aldrich), and 0.2% Triton X (93443, Sigma-Aldrich) in PBS (AM9625, ThermoFisher Scientific). Primary antibodies (**Table S9**) were diluted in blocking buffer and incubated overnight at 4°C. Samples were then washed three times with 1% BSA in PBS and incubated with the secondary antibody (**Table S9**) diluted in blocking buffer for 1 hour at RT. After three additional washes with PBS, DAPI was added for 2 min at RT (1:10000).

Image analysis

Images were acquired using the Opera Phenix Plus High-Content Screening System (HH14001000, PerkinElmer, Waltham, MA, USA), and the Harmony analysis software was used for quantification. Samples were analyzed by imaging six evenly spaced fields, each consisting of 3x3 images, resulting in 54 total images per sample at 40x magnification. Images were acquired as z-stacks, flat-field corrected, and converted to a 2D image using maximum intensity projection. Somatic quantification of α-synuclein (α-syn) and phospho-α-synuclein (p-syn) was performed by finding TH+ (avg. intensity > 50) nuclei (DAPI mask) and averaging the intensity of the target channel (α-syn or p-syn) in the selected area.
Statistical analysis was performed in GraphPad Prism 8 using an ordinary two-way ANOVA (factors: genotype and treatment) and the Tukey-Kramer test to compare all pairs of means, which accounts for multiple comparisons.

Supplemental references

1. Wang, X., A. Wirth, and L. Wang. *Structure-Based Statistical Features and Multivariate Time Series Clustering*. in *Seventh IEEE International Conference on Data Mining (ICDM 2007)*. 2007.
2. Mietus, J.E., et al., *The pNNx files: re-examining a widely used heart rate variability measure.* Heart, 2002. **88**(4): p. 378-380.
3. Lubba, C.H., et al., *catch22: CAnonical Time-series CHaracteristics.* Data Mining and Knowledge Discovery, 2019. **33**(6): p. 1821-1852.
4. Fulcher, B.D. and N.S. Jones, *hctsa: A Computational Framework for Automated Time-Series Phenotyping Using Massive Feature Extraction.* Cell Systems, 2017. **5**(5): p. 527-531.e3.
5. Müller, J., et al., *High-resolution CMOS MEA platform to study neurons at subcellular, cellular, and network levels.* Lab Chip, 2015. **15**(13): p. 2767-80.
6. Kriks, S., et al., *Dopamine neurons derived from human ES cells efficiently engraft in animal models of Parkinson’s disease.* Nature, 2011. **480**(7378): p. 547-551.
7. Ronchi, S., et al., *Electrophysiological Phenotype Characterization of Human iPSC-Derived Neuronal Cell Lines by Means of High-Density Microelectrode Arrays.* Advanced Biology, 2021. **n/a**(n/a): p. 2000223.
8. Haenseler, W., et al., *Excess α-synuclein compromises phagocytosis in iPSC-derived macrophages.* Sci Rep, 2017. **7**(1): p. 9003.
9. Fernandes, Hugo J.R., et al., *ER Stress and Autophagic Perturbations Lead to Elevated Extracellular α-Synuclein in GBA-N370S Parkinson's iPSC-Derived Dopamine Neurons.* Stem Cell Reports, 2016. **6**(3): p. 342-356.
10. Fedele, S., et al., *Expansion of human midbrain floor plate progenitors from induced pluripotent stem cells increases dopaminergic neuron differentiation potential.* Scientific Reports, 2017. **7**(1): p. 6036.
11. McInnes, L., J. Healy, and J. Melville, *Umap: Uniform manifold approximation and projection for dimension reduction.* arXiv preprint arXiv:1802.03426, 2018.
12. Lee, E.K., et al., *Non-linear dimensionality reduction on extracellular waveforms reveals cell type diversity in premotor cortex.* eLife, 2021. **10**: p. e67490.
13. Blondel, V.D., et al., *Fast unfolding of communities in large networks.* Journal of statistical mechanics: theory and experiment, 2008. **2008**(10): p. P10008.
14. Rubinov, M. and O. Sporns, *Complex network measures of brain connectivity: Uses and interpretations.* NeuroImage, 2010. **52**(3): p. 1059-1069.
